# Cytotoxic CD4^+^ T cells induce age-associated myelopoiesis through CCL5–CCR5 signaling

**DOI:** 10.1038/s43587-026-01209-9

**Published:** 2026-08-26

**Authors:** Enrique Gabandé-Rodríguez, Gonzalo Soto-Heredero, Elisa Carrasco, Carlos Anerillas, José Ignacio Escrig-Larena, Jorge Martínez-Cano, Sandra Delgado-Pulido, Isaac Francos-Quijorna, Gabriela Desdín-Micó, Tatiana Smirnova, Manuel M. Gómez de las Heras, Álvaro Fernández-Almeida, Alice Leandri, Andrea Cugurra, Eva María Blanco, Elia Winand-Osete, Pablo Ramírez-Ruiz de Erenchun, Virginia Zorita, Camilla Bean, Valentin Basset, Françoise Solly, Amanda Garrido, Rafael de Cabo, Santos Mañes, Manolis Pasparakis, Matteo Iannacone, César Cobaleda, Olaia Naveiras, Myriam Gorospe, María Mittelbrunn

**Affiliations:** 1 Tissue and Organ Homeostasis Program, Cell–Cell Communication and Inflammation Unit, Centro de Biología Molecular Severo Ochoa (Consejo Superior de Investigaciones Científicas-Universidad Autónoma de Madrid), Madrid, Spain; 2 Department of Molecular Biology, Universidad Autónoma de Madrid (UAM), Madrid, Spain; 3 Division of Immunology, Transplantation, and Infectious Diseases, IRCCS San Raffaele Scientific Institute, Milan, Italy; 4 Vita-Salute San Raffaele University, Milan, Italy; 5 Department of Biology, Universidad Autónoma de Madrid, Madrid, Spain; 6 Instituto Universitario de Biología Molecular-IUBM (Universidad Autónoma de Madrid), Madrid, Spain; 7 Instituto Ramón y Cajal de Investigación Sanitaria (IRYCIS), Madrid, Spain; 8 Laboratory of Genetics and Genomics, National Institute on Aging Intramural Research Program, National Institutes of Health, Baltimore, MD USA; 9 Interactions with the Environment Program, Immune System Development and function Unit, Centro de Biología Molecular Severo Ochoa (Consejo Superior de Investigaciones Científicas-Universidad Autónoma de Madrid), Madrid, Spain; 10 Laboratory of Regenerative Hematopoiesis, Department of Biomedical Sciences, University of Lausanne, Lausanne, Switzerland; 11 Hematology Service and Hematology Translational Research Unit, Departments of Oncology and Laboratory Medicine, Lausanne University Hospital and University of Lausanne, Lausanne, Switzerland; 12 Service and Central Laboratory of Hematology, Lausanne University Hospital (CHUV), Lausanne, Switzerland; 13 Department of Biology, University of Padua, Padua, Italy; 14 Centro Nacional de Investigaciones Cardiovasculares (CNIC), Madrid, Spain; 15 Departmental Faculty of Medicine, UniCamillus–Saint Camillus International University of Health and Medical Sciences, Rome, Italy; 16 Veneto Institute of Molecular Medicine, Padua, Italy; 17 Translational Gerontology Branch, National Institute on Aging Intramural Research Program, National Institutes of Health, Baltimore, MD USA; 18 Department of Immunology and Oncology, Centro Nacional de Biotecnología, Consejo Superior de Investigaciones Científicas (CSIC), Madrid, Spain; 19 Institute of Genetics, Centre for Molecular Medicine (CMMC) and Cologne Excellence Cluster on Cellular Stress Responses in Aging-Associated Diseases (CECAD), University of Cologne, Cologne, Germany

**Keywords:** Lymphocytes, Physiology

## Abstract

Age-associated hematopoietic skewing results in an increase in the neutrophil-to-lymphocyte ratio, which serves as a strong predictor of all-cause mortality in older adults, although its causes are incompletely understood. Here we show that cytotoxic CD4^+^ T lymphocytes accumulate in the bone marrow of mice during aging and induce myelopoiesis, increasing the neutrophil-to-lymphocyte ratio. T cell receptor-dependent induction of mitochondrial stress and activation of STING upregulates the chemokine CCL5 in CD4^+^ T lymphocytes. During aging, hematopoietic stem cells and downstream myeloid progenitors upregulate CCR5, the primary receptor for CCL5. Genetic ablation of *Ccr5* in hematopoietic progenitors mitigates T cell-induced myeloid skewing and neutrophil expansion. Pharmacological blockade of CCR5 using the Food and Drug Administration-approved drug maraviroc normalizes myelopoiesis, reduces circulating and tissue-infiltrating neutrophils, and improves multiple aging-related biomarkers and functional outcomes in aged mice. Together, these findings demonstrate a T cell–bone marrow axis that exacerbates age-associated decline and highlight CCR5 inhibition as a potential geroprotective strategy.

## Abstract (web summary)

Gabandé-Rodríguez, Soto-Heredero and colleagues identify an accumulation of cytotoxic CD4^+^ T cells producing CCL5 in the bone marrow during aging, linked to age-associated myelopoiesis. CCR5 blockade reverses this axis and improves healthspan in aged mice.

## Main

Age-related hematopoietic skewing favors myeloid over lymphoid differentiation, resulting in an elevated neutrophil-to-lymphocyte ratio (NLR) in the circulation^1^. Recent findings suggest that rebalancing of hematopoiesis may be a strategy to modify age-associated immune dysfunction. Accordingly, depletion of myeloid-biased hematopoietic stem cells (HSCs) can rejuvenate the immune system in old mice^2^. Myeloid-biased hematopoiesis also characterizes human aging^3^. Remarkably, a high NLR serves as a strong predictor of mortality in virtually all age-associated human diseases^4–7^. Moreover, abrogation of myelopoiesis by administering the vitamin A metabolite 4-oxo-retinoic acid preserves long-term cardiac function in patients with myocardial infarction^8^. Therefore, understanding the mechanisms driving enhanced myelopoiesis and a heightened NLR during aging may critically change the way in which multiple human age-associated diseases are managed.

Recent reports indicate that T cells infiltrate the bone marrow (BM) under acute psychological stress, prolonged starvation or autoimmune disease, eventually leading to increased myelopoiesis and circulatory neutrophils^9–12^. Here we investigate whether T cells drive age-related changes in hematopoiesis in mice, and assess pharmacological strategies to restore hematopoietic homeostasis, normalize the NLR and improve health status in aged mice.

## Results

### CD4^+^ T cells induce age-associated myelopoiesis

**Fig. 1:**
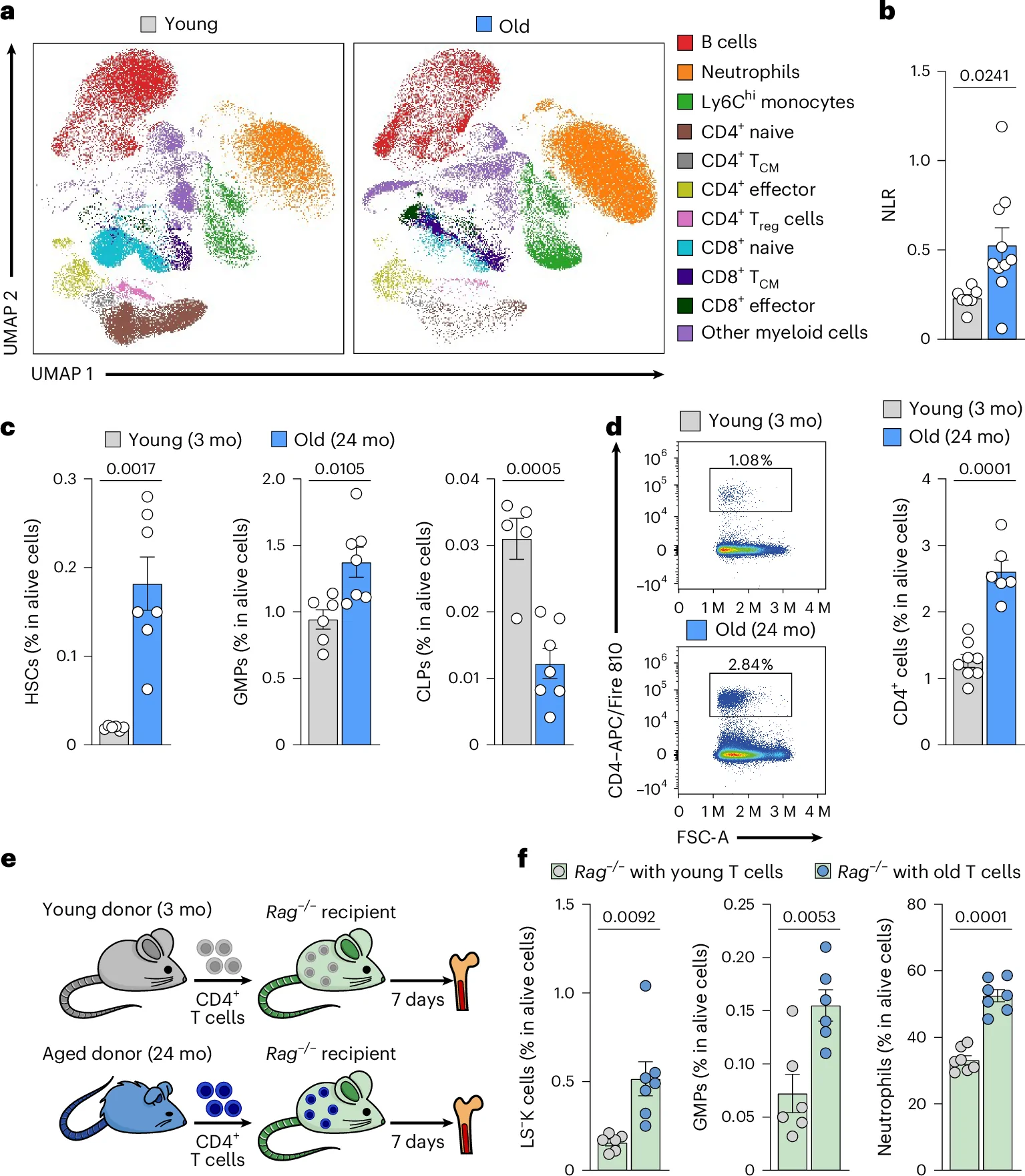
Aging-associated CD4^+^ T cells drive myeloid skewing in mice. **a**, UMAP with Cluster-X overlay showing the distribution of clusters of immune cells identified by multiparametric spectral flow cytometry in the circulation of representative young (3 months old (mo)) and old (24 mo) mice. **b**, Quantification of NLR in the circulation of young (3 mo) and old (24 mo) mice (*n* = 7 young and *n* = 10 old mice). **c**, Percentage of HSCs, GMPs and CLPs in the BM from young (3 mo) and old (24 mo) mice assessed by flow cytometry (*n* = 6 young (HSCs and GMPs), *n* = 5 young (CLPs) and *n* = 7 old mice). **d**, Representative flow cytometry plots and quantification of the percentage of CD4^+^ cells in the BM of young (3 mo) and old (24 mo) mice (*n* = 8 young and *n* = 6 old mice). **e**, Schematic diagram depicting the adoptive transfer of CD4^+^ cells isolated from young (3 mo) or old (24 mo) into young *Rag1*^*−/−*^ recipients. **f**, Quantification of the percentage of LS^*−*^K, GMPs and neutrophils in the BM of recipient animals 7 days after the adoptive transfer (*n* = 6 young (LS^−^K and GMPs), *n* = 7 young (neutrophils), *n* = 6 old (GMPs) and *n* = 7 old (LS^−^K and neutrophils) mice). Data are presented as mean ± s.e.m. Exact *P* values are shown above bar plots. Statistics and remaining *P* values are shown in the Source Data. FSC-A, forward scatter area; M, millions. Source Data

**Extended Data Fig. 1:**
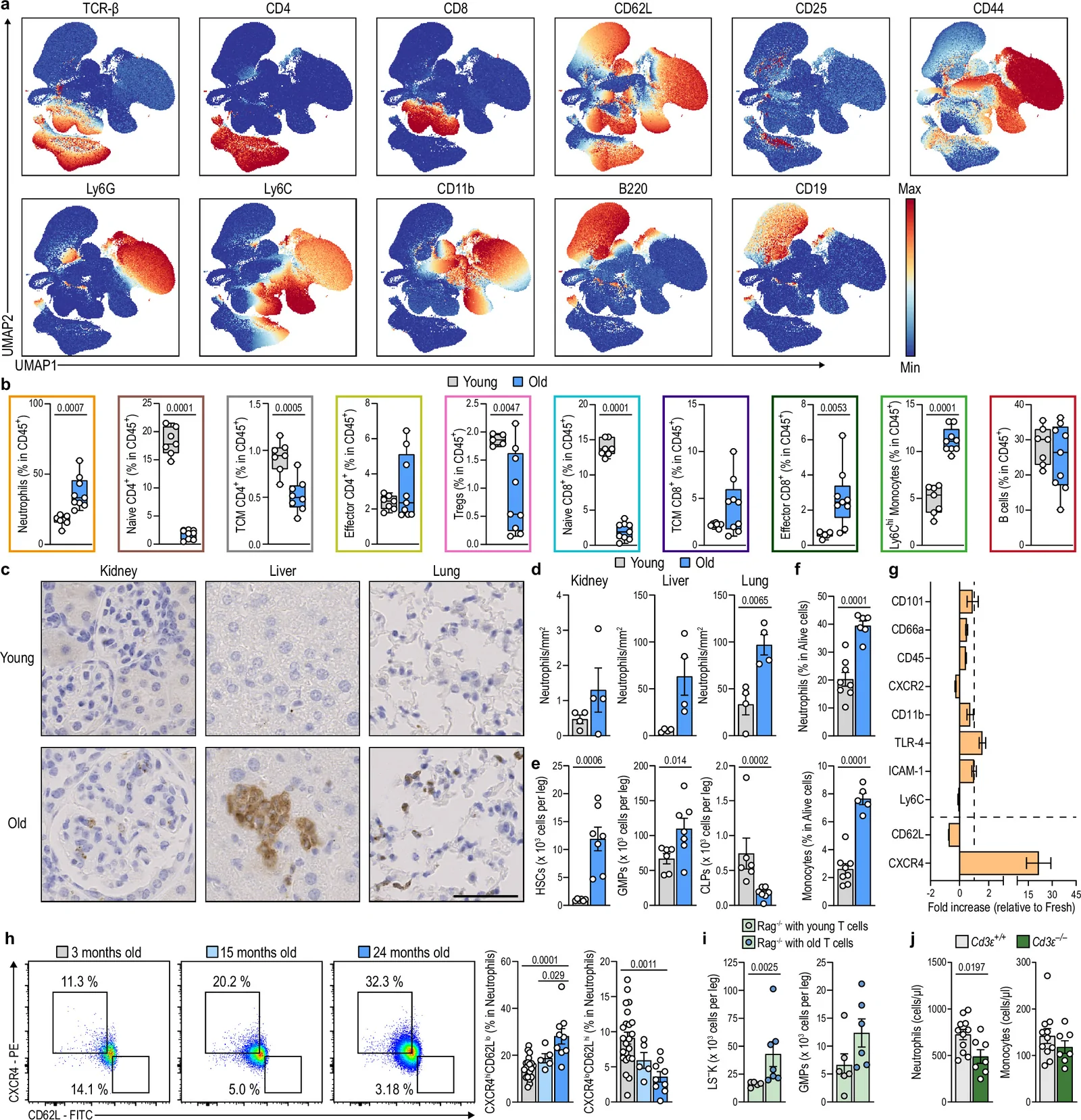
Enhanced myelopoiesis and accumulation of neutrophils in tissues from aged mice. **a**, UMAP overlay representation of the expression levels of markers used to identify the different populations of blood cells shown in Fig. 1a and Extended Data Fig. 7c. **b**, Boxplots comparing the percentage of the different clusters in blood from young (3 mo) and old (24 mo) mice (n = 5 (Tregs) or 6 (CD8^+^ TEM) or 7 (rest) young mice and 8 (CD4^+^ TCM) or 9 (rest) old mice. **c**,**d**, Representative immunohistochemistry images (**c**) and quantification (**d**) of MPO-positive neutrophils in kidney, liver, and lung sections from young (3 mo) and old (24 mo) mice (n = 4 young and 4 old mice). Scale bar: 50 μm. **e**, Quantification of the number of HSCs, GMPs and CLPs per leg in the BM from young (3 mo) and old (24 mo) mice assessed by flow cytometry (n = 5 (CLPs) or 6 (HSCs and GMPs) young mice and 7 old mice). **f**, Percentage of neutrophils and Ly6C^hi^ monocytes in the BM from young (3 mo) and old (24 mo) mice assessed by flow cytometry (n = 8 young and 5 (Ly6C^hi^ monocytes) or 6 (neutrophils) old mice). **g**, Representative dot plots and quantification of CXCR4^hi^ CD62L^lo^ and CXCR4^lo^ CD62L^hi^ neutrophils in the circulation of young (3 mo) middle-aged (15 mo) and old (24 mo) mice (n = 23 (3mo), 5 (15 mo) and 9 (24mo) mice). **h**, Expression of different surface markers showing an upregulation of pro-inflammatory markers in CXCR4^hi^CD62L^lo^ relative to CXCR4^lo^CD62L^hi^ neutrophils (n = 5 (CD101, CD66a), 6 (CXCR4), 7 (TLR-4), 9 (CD11b), 12 (CD62L, Ly6c, CXCR2, CD45), 16 (ICAM-1) mice). **i**, Quantification of the number of LS^−^K and GMPs in the BM of recipient animals 7 days after the adoptive transfer (n = 5 (LS^−^K) or 6 (GMPs) young and 6 (GMPs) or 7 (LS^−^Ks) old mice). **j**, Percentage of neutrophils and Ly6C^hi^ monocytes in blood from old (17 mo) T cell-deficient *Cd3e*^*−*/*−*^ and control mice (n = 11 *Cd3e*^+/+^ and 7 *Cd3e*^*−*/*−*^ mice). Data are presented as mean values ± s.e.m. Box-and-whisker plots show the median, the maximum, the minimum and the 25th and 75th percentiles. Exact *P* values are shown above bar plots. Statistics and rest of *P* values are shown in Source data. Source Data

**Extended Data Fig. 2:**
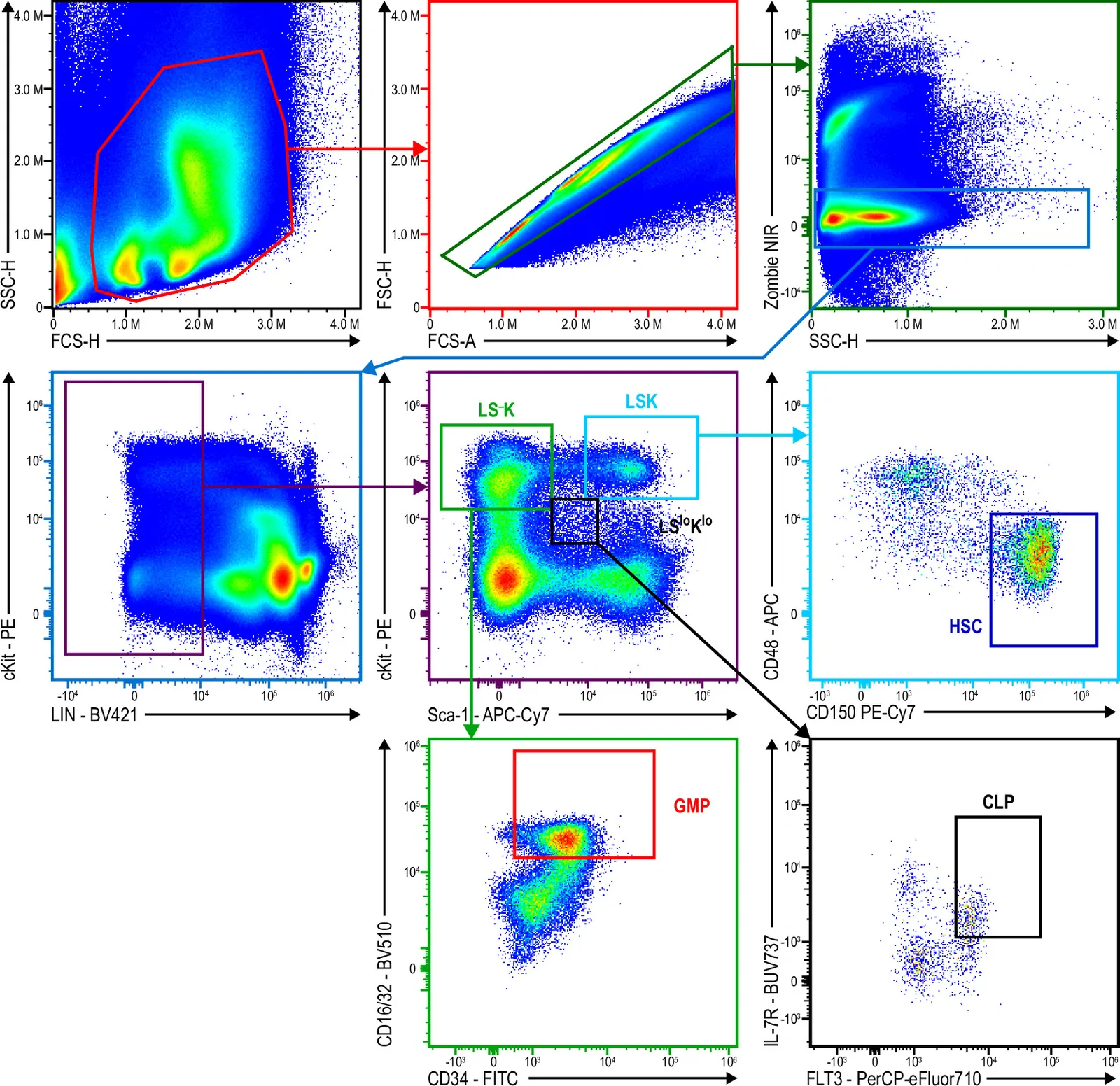
Gating strategy to identify hematopoietic precursors in the bone marrow. Gating strategy followed to identify LS^−^K (Lineage^−^Sca^−^cKit^+^) cells, GMPs (LS^−^K; CD16/32^+^CD34^+^), LSK (Lineage^−^Sca^+^cKit^+^) cells, HSCs (LSK; CD48^+^CD150^−^), LS^lo^K^lo^ (Lineage^−^Sca^lo^cKit^lo^) cells and CLPs (LS^lo^K^lo^; IL-7R^+^FLT3^+^).

We performed multiparametric spectral flow cytometry to analyze the percentage of different blood populations in old (24-month-old) versus young (3-month-old) mice. Uniform manifold approximation and projection (UMAP) representation showed increased numbers of circulating neutrophils and monocytes, together with reduced numbers of circulating lymphocytes (Fig. 1a and Extended Data Fig. 1a,b), resulting in an increased NLR (Fig. 1b) and increased infiltration in the kidney, lung and liver in old compared with young mice (Extended Data Fig. 1c,d). According to previous data, we confirmed that this rise in neutrophils correlated with a myeloid skewing of hematopoiesis during aging. Aged mice exhibited increased percentages and numbers of HSCs and granulocyte–monocyte precursors (GMPs) but decreased percentages and numbers of common lymphoid progenitors (CLPs) (Fig. 1c and Extended Data Figs. 1e and 2), together with increased frequencies of neutrophils and monocytes in the BM (Extended Data Fig. 1f). To investigate how aging shapes neutrophil phenotypes, we assessed CXCR4 and CD62L levels to distinguish fresh (CXCR4^lo^CD62L^hi^) and aged (CXCR4^hi^CD62L^lo^) neutrophils by flow cytometry. Compared with fresh neutrophils, aged neutrophils displayed an activated profile characterized by the upregulation of ICAM-1, TLR-4, CD11b, CD66a or CD101, and the downregulation of CXCR2 (Extended Data Fig. 1g). Of note, we observed a higher percentage of circulating CXCR4^hi^CD62L^lo^ neutrophils and a decreased percentage of CXCR4^lo^CD62L^hi^ naive neutrophils in old mice (Extended Data Fig. 1h).

**Extended Data Fig. 3:**
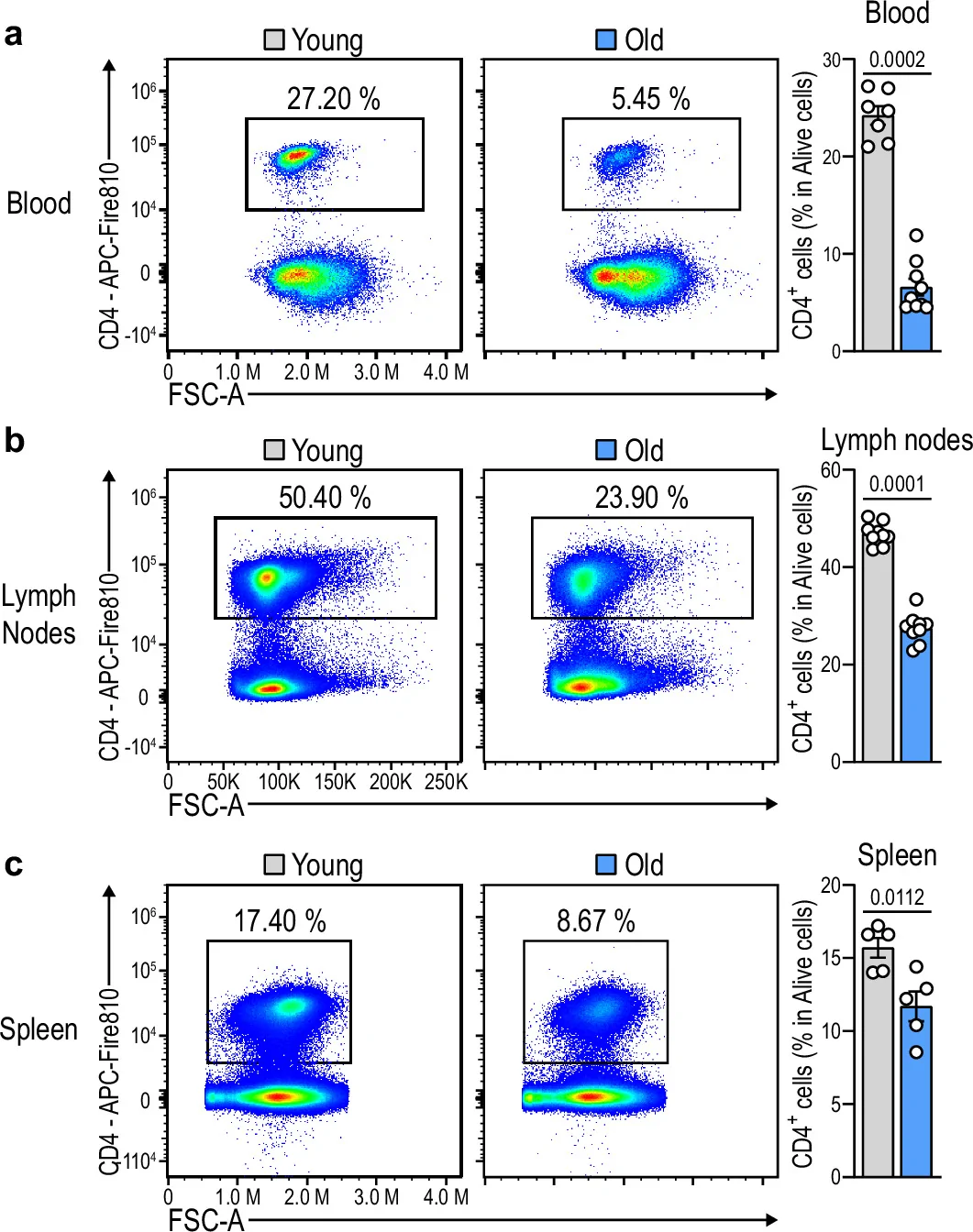
Decreased percentage of CD4^+^ T cells in the blood, lymph nodes and spleen during ageing. **a-c**, Representative flow cytometry plots (left) and quantification (right) of the percentage of CD4^+^ cells in the blood (**a**), lymph nodes (**b**) and spleen (**c**) from young (3 mo) and old (24 mo) mice (n = 5 (spleen) or 7 (blood) or 9 (lymph nodes) young mice and 5 (spleen) or 9 (blood, lymph nodes) old mice). Data are presented as mean values ± s.e.m. Exact *P* values are shown above bar plots. Statistics are shown in Source data. Source Data

Because recent evidence supports the idea that T cells infiltrate the BM and influence hematopoiesis during autoimmune conditions^11^, we wondered whether this mechanism operates in old mice. We observed that, unlike in the blood, lymph nodes and spleen, old mice exhibited an increased percentage of CD4^+^ T cells in the BM (Fig. 1d and Extended Data Fig. 3a–c). To directly dissect the capacity of aged CD4^+^ T cells to induce myelopoiesis, we performed adoptive transfer experiments of CD4^+^ T cells from young and old mice into T cell and B cell-deficient recipient (*Rag1*^*−/−*^) mice^13^ and analyzed the frequency of BM hematopoietic precursors 7 days post-transfer (Fig. 1e). Compared with the transfer of CD4^+^ T cells from young mice, infusion of CD4^+^ T cells from old mice led to enhanced frequencies of Lin^−^Sca-1^−^c-Kit^+^ (LS^−^K) cells and downstream GMPs, together with an increased frequency of BM neutrophils (gated as CD45^+^, CD11b^+^, Ly6c^int^ and Ly6G^+^) (Fig. 1f and Extended Data Fig. 1i), indicating that old CD4^+^ T cells promote myelopoiesis. Further supporting a role of T cells in the regulation of myeloid cell generation, old *Cd3e*^*−/−*^ mice lacking T cells had lower levels of circulating neutrophils and monocytes than age-matched control mice (Extended Data Fig. 1j).

Taken together, these results indicate that T cells accumulate in the BM during aging, correlating with enhanced myelopoiesis and neutrophil output.

### CD4^+^ T lymphocytes producing CCL5 accumulate in the BM with age

**Fig. 2:**
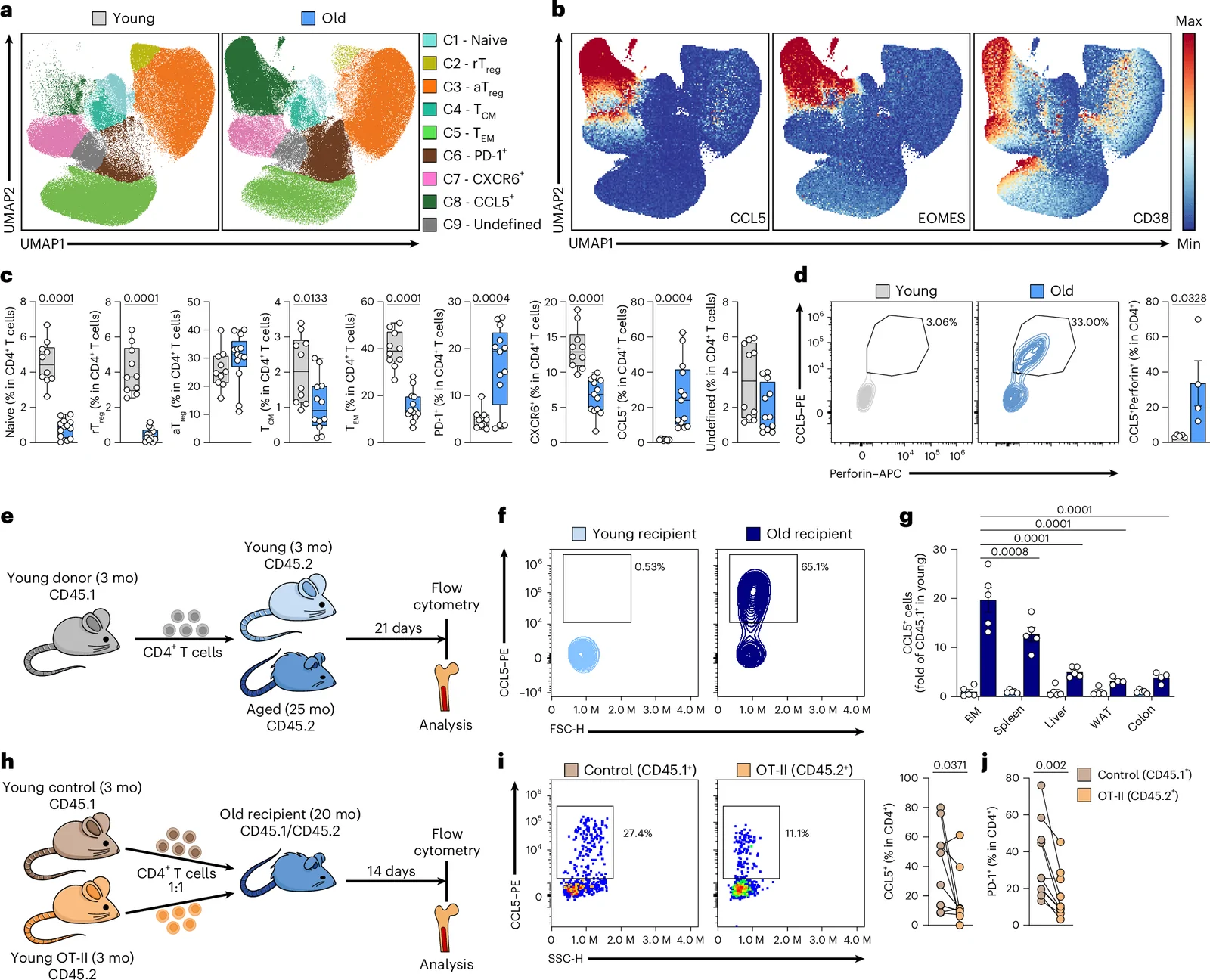
Age-dependent TCR stimulation promotes BM accumulation of cytotoxic CD4^+^ T cells. **a**, Representative UMAP with Cluster-X overlay showing the distribution of the nine clusters of CD4^+^ T cells identified by multiparametric spectral flow cytometry in the BM from young (3 mo) and old (24 mo) mice. **b**, UMAP representation of the expression levels of representative markers used to identify the CD4^+^ CTLs. **c**, Boxplots comparing the percentage of cells in each T cell subset in young (3 mo) and old (20 mo) mice (*n* = 10 young, *n* = 12 old (unidentified, T_CM_) or *n* = 13 old (naive, rT_reg_, aT_reg_, T_EM_, CXCR6^+^, CCL5^+^, PD-1^+^) mice). **d**, Representative flow cytometry plots and quantification of the percentage of CCL5^+^Perforin^+^CD4^+^ cells in the BM of young (3 mo) and old (24 mo) mice (*n* = 5 young and *n* = 4 old mice). **e**, Schematic diagram depicting the adoptive transfer of CD45.1^+^ T cells isolated from 3 mo mice into either young (3 mo) or old (24 mo) CD45.2^+^ recipients. **f**, Representative contour plots and quantification of the percentage of BM CD4^+^CD38^+^CCL5^+^ T cells in young or old recipients 21 days after the adoptive transfer of CD4^+^ T cells (*n* = 5 young (BM, spleen, liver, colon), *n* = 4 young (white adipose tissue), *n* = 5 old (BM, spleen, liver) or *n* = 4 old (white adipose tissue and colon) mice per group). **g**, Fold increase in CD4^+^CD38^+^CCL5^+^ T cells in the indicated organs from young and old recipients (*n* = 5 mice per group). **h**, Schematic diagram depicting the mixed adoptive transfer of CD45.1^+^ control and CD45.2^+^ OT-II derived cells into old (20 mo) CD45.1.2^+^ recipients. **i**, Representative dot plots and quantification of the percentage of BM CCL5^+^CD4^+^ cells in either CD45.1^+^ control or CD45.2^+^ OT-II mice 14 days after the adoptive transfer (*n* = 10 mice per group). **j**, Percentage of PD-1^+^CD4^+^ cells in CD45.1^+^ control or CD45.2^+^ OT-II transferred cells (*n* = 10 recipient mice). Data are presented as mean ± s.e.m. Box-and-whisker plots show the median, maximum, minimum and the 25th and 75th percentiles. Exact *P* values are shown above bar plots. Statistics and remaining *P* values are shown in the Source Data. FSC-H, forward scatter height; SSC-H, side scatter height; WAT, white adipose tissue. Source Data

**Extended Data Fig. 4:**
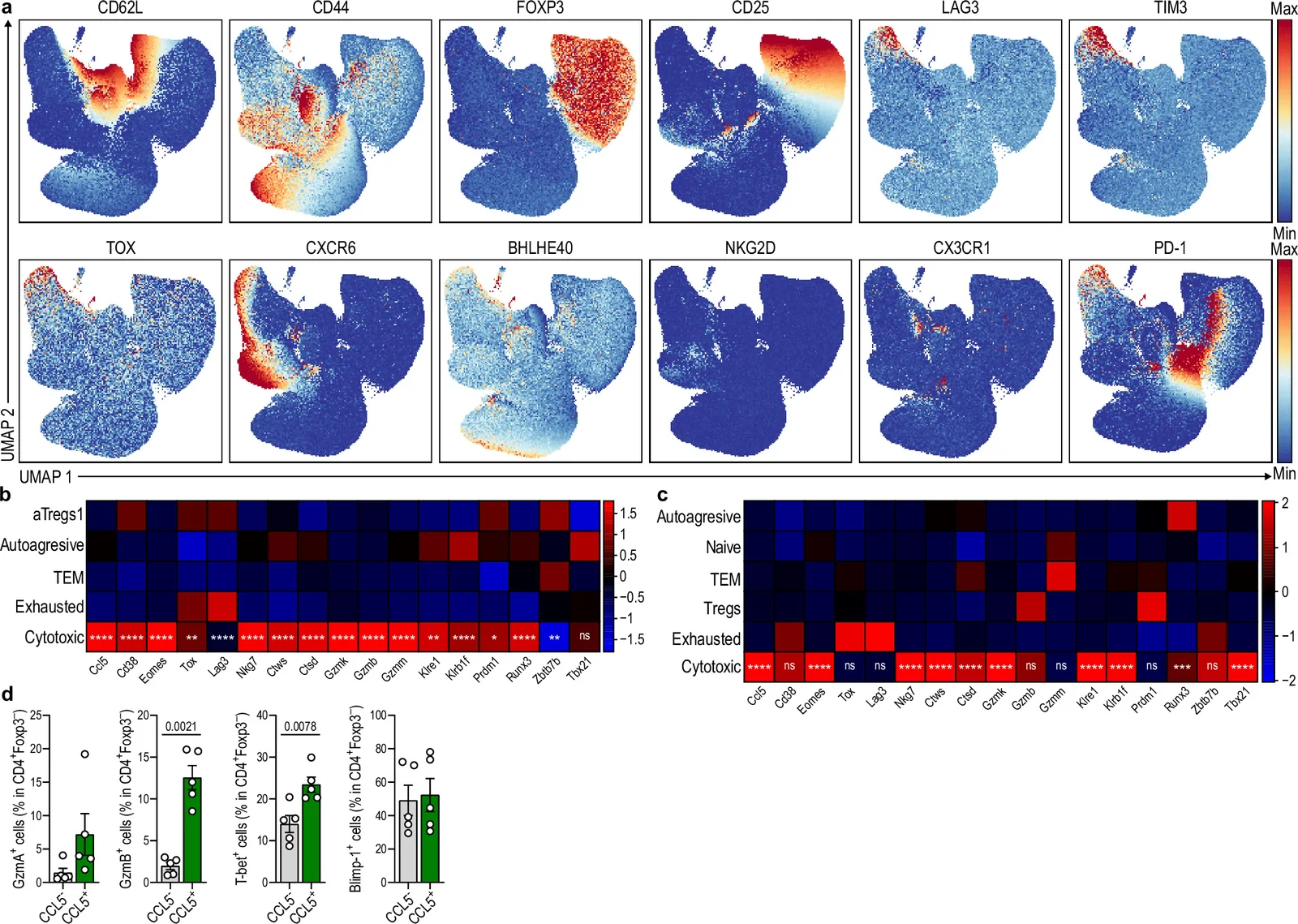
Molecular characterization of CD4^+^ CTLs. **a**, UMAP representation of the expression levels of markers used to identify the different populations of BM CD4^+^ cells shown in Fig. 2a. **b**, Heat map showing the differentially abundant RNAs across the different clusters in splenic CD4^+^ cells from a publically available scRNAseq dataset^14^. **c**, Heat map showing the differentially abundant RNAs across the different clusters in bone marrow CD4^+^ cells from a publically available scRNAseq dataset^16^. **d**, Percentage of Gzm A^+^, Gzm B^+^, T-bet^+^ and Blimp-1^+^ cells in CD4^+^ CCL5^+^ and CD4^+^ CCL5^−^ cells in the BM from old mice (n = 5 old mice). Data are presented as mean values ± s.e.m. Exact *P* values are shown above bar plots. Statistics and rest of *P* values are shown in Source data. Source Data

Based on single-cell analyses that have uncovered the heterogeneity of T cells during aging in different organs^14,15^, we implemented a panel of antibodies to explore the diversity of CD4^+^ T cells in the BM during aging using spectral flow cytometry (Fig. 2a,b and Extended Data Fig. 4a). Analysis of CD4^+^ T cells from the BM by UMAP and unbiased clustering identified nine clusters, including naive T cells (cluster 1 or ‘naive’), resting and activated T regulatory cells (clusters 2 and 3 or ‘rT_reg_’ and ‘aT_reg_’, respectively) and central memory T cells (cluster 4 or ‘T_CM_’). The remaining clusters included different subsets classically included under the term ‘effector memory’. Thus, we identified effector memory T cells (cluster 5 or ‘T_EM_’), PD-1^+^ T cells (cluster 6 or ‘PD-1^+^’), a cluster defined by the coexpression of CXCR6 and CD38 (cluster 7 or ‘CXCR6^+^’) and a CCL5^+^ cluster (cluster 8 or ‘CCL5^+^’). Finally, we identified a cluster lacking expression of most of the assessed markers (cluster 9 or ‘undefined’) (Fig. 2a,b and Extended Data Fig. 4a). Critically, the vast majority of BM CD4^+^ T cells showing a significant accumulation in old mice grouped in the CCL5^+^ cluster, accounting for >25% of the CD4^+^ cells, while it was negligible in young mice (CCL5^+^: mean young, 1.64; mean old, 26.78; *P* < 0.0001) (Fig. 2c). The CCL5^+^ cluster was characterized by expressing high levels of the activation marker CD38 and the transcription factor Eomesodermin (EOMES) together with low expression of CD44 (Fig. 2b and Extended Data Fig. 4a), resembling cytotoxic T cells (CD4^+^ CTLs)^14^. Reanalysis of publicly available single-cell RNA sequencing (scRNA-seq) datasets in spleen and BM from old mice^14,16^ showed increased levels of typical cytotoxicity and natural killer-associated transcripts such as *Nkg7*, *Ctsd*, *Ctsw*, *Gzmb*, *Gzmk*, *Gzmm*, *Klre1* and *Klrb1f*, and transcription factors like *Tbx21*, encoding T-bet, or *Runx3* in the cytotoxic cluster (Extended Data Fig. 4b,c). Positive expression of perforin, T-bet, granzyme A and granzyme B (GzmB) by flow cytometry in most BM CD4^+^ CCL5^+^ cells from old mice further confirmed the cytotoxic identity of this cluster (Fig. 2d, Extended Data Fig. 4d and Source Data).

### TCR stimulation in an aged milieu induces CCL5^+^CD4^+^ CTL differentiation

An increased abundance of CD4^+^ CTLs has been reported in aged individuals^17^ and in conditions associated with repeated T cell activation, including infection and cancer^18^. To investigate whether the differentiation of T cells toward CCL5^+^CD4^+^ CTL is triggered by exposure to an aged environment, we performed heterochronic adoptive transfer of CD45.1^+^CD4^+^ T cells isolated from young mice into either young or old CD45.2^+^ recipient mice (Fig. 2e). Analysis of T cells 21 days post-transfer showed a CD4^+^ CTL profile in the BM (Fig. 2f) and a remarkable 18.5-fold increase in old mice compared to young recipients (Fig. 2g). Of note, comparison of the percentage of CD45.1^+^CD4^+^ CTLs in different tissues including the liver, spleen, white adipose tissue and the colon, revealed that donor cells were particularly enriched in the BM during aging, suggesting that the BM is a preferential site for CD4^+^ CTL accumulation (Fig. 2g).

To assess whether the conversion to CCL5^+^CD4^+^ CTL in an aged environment was due to chronic T cell receptor (TCR) stimulation or to exposure to inflammaging-associated cytokines, we adoptively transferred CD4^+^ cells isolated from young CD45.1 and OT-II CD45.2^+^ mice into old CD45.1.2^+^ recipients in a 1:1 ratio and analyzed the differential infiltration of donor CD4^+^ cells into the BM 14 days later (Fig. 2h). CD4^+^ cells from OT-II mice are genetically engineered to express a TCR that exclusively recognizes a specific peptide of chicken ovalbumin (OVA). In the absence of this peptide, OT-II CD4^+^ cells cannot engage TCR-dependent activation while remaining susceptible to cytokine-mediated activation^19^. We detected a decrease in the percentage of both CCL5^+^ and PD-1^+^CD45.2^+^ OT-II T cells in the BM of recipient mice 14 days after the adoptive transfer (Fig. 2i,j), suggesting that TCR signaling is required for CD4^+^ CTL differentiation in an aged environment.

These results support the notion that exposure to an aged environment induces the differentiation of CD4^+^ cells into CD4^+^ CTLs, requiring TCR stimulation.

### BM CD4^+^ CTLs exhibit altered mitochondria and STING-dependent CCL5 production

**Extended Data Fig. 5:**
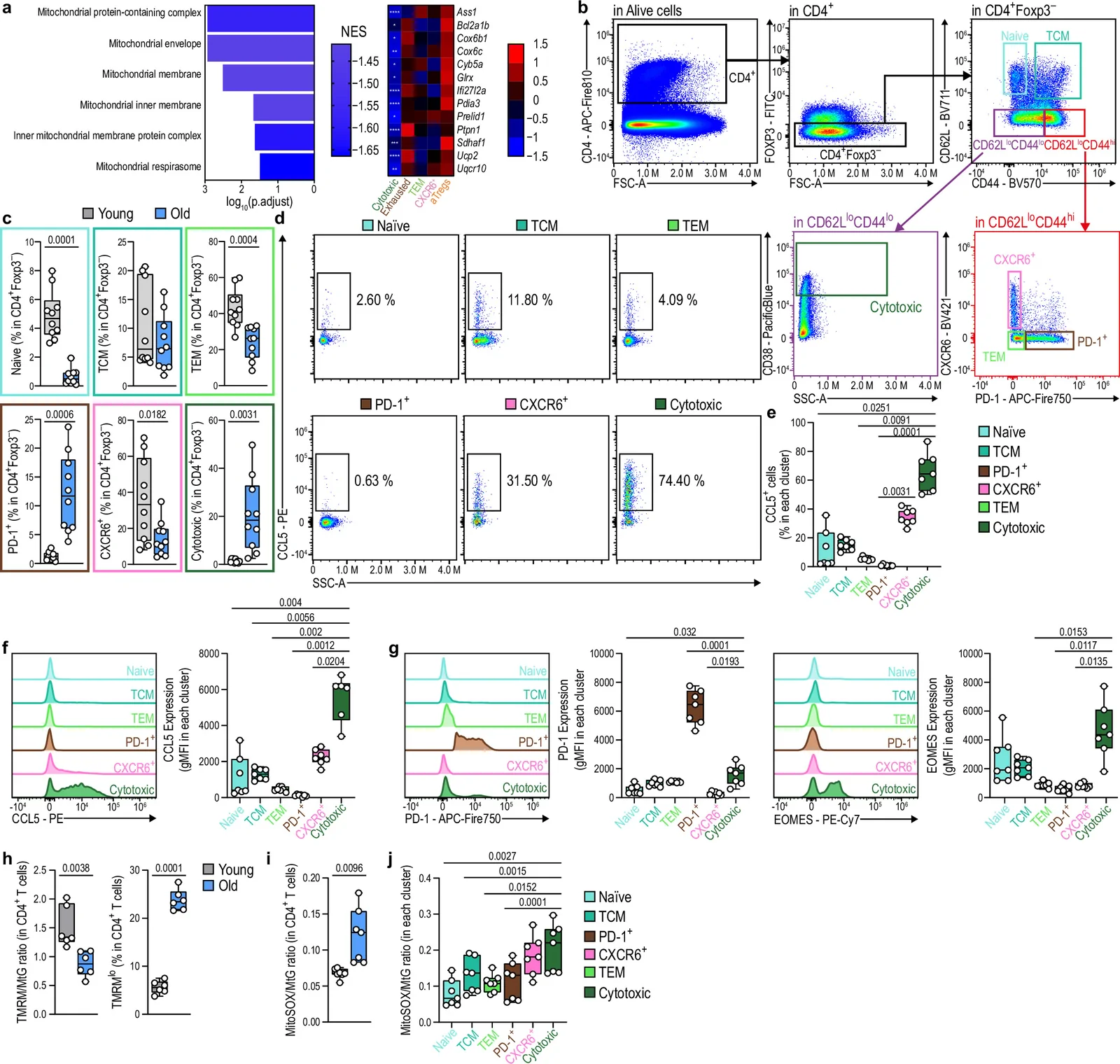
Extracellular identification and analysis of mitochondrial membrane potential in CD4^+^ CTLs from old mice. **a**, GSEA analysis of differentially represented pathways related to mitochondrial function in CD4^+^ CTLs compared with the other differentiated CD4^+^ cells (left) and heat map showing the differentially abundant RNAs across the different clusters (right)^14^. **b**, Gating strategy of the different CD4^+^ T cell clusters by extracellular-based manual gating. First, T regulatory cells were excluded by gating on CD4^+^Foxp3^−^ cells (for extracellular staining, Foxp3 was substituted by CD25). Then, naïve (CD4^+^CD62L^hi^CD44^lo^) and TCM (CD4^+^CD62L^hi^CD44^hi^) cells were identified. After that, CD44^hi^CD62L^lo^ cells or CD44^lo^CD62L^lo^ were gated. CXCR6^+^ (CD4^+^CD62L^lo^CD44^hi^PD-1^*−*^CXCR6^hi)^, PD-1^+^ (CD4^+^CD62L^lo^CD44^hi^CXCR6^−^PD-1^+^) and TEM (CD4^+^CD62L^lo^CD44^hi^CXCR6^−^PD-1^*−*^) cells were identified in the CD44^hi^CD62L^lo^ population. CD4^+^ CTLs were identified as CD4^+^CD62L^lo^CD44^lo^CD38^hi^. **c**, Quantification of the percentage of each cluster in CD4^+^Foxp3^−^ cells in BM samples from young (3 mo) and old (24 mo) mice (n = 9 (PD-1^+^) or 10 (rest) young and 10 old mice). The frequencies and percentages of cells on each cluster are similar to the observed by intracellular staining. **d**, **e**, Representative flow cytometry plots (**d**) and quantification (**e**) of CCL5^+^CD4^+^ cells in each cluster when identified by extracellular gating in BM samples from old (24 mo) mice. A high enrichment of CCL5^+^ cells is observed in the population corresponding to CD4^+^ CTLs (n = 6 (PD-1^+^) or 7 (rest) young and 7 old mice). **f**, Representative histogram and quantification showing the expression of CCL5 in the different clusters (n = 6 (CD4^+^ CTLs) or 7 young and 7 old mice). *P* values indicate comparisons between each cluster and the cytotoxic cluster. All other statistical comparisons are provided in the Source Data. **g**, Representative histogram and quantification showing the expression of PD-1 (**up**) and EOMES (**down**) in the different clusters (n = 7 young and 7 old mice). *P* values indicate comparisons between each cluster and the cytotoxic cluster. All other statistical comparisons are provided in the Source Data. **h**, **i**, Quantification of the TMRM/MtG ratio (**left**) and the percentage of TMRM^lo^ CD4^+^ cells (**right**) in the BM of young (3mo) and old (24mo) mice (n = 6 young and 6 old mice). **i**, Quantification of the MitoSOX/MtG ratio in CD4^+^ cells from the BM of young (3 mo) and old (24 mo) mice (n = 7 young and 7 old mice). **j**, Quantification of the MitoSOX/MtG ratio in the clusters identified by extracellular gating in BM from old (24mo) mice (n = 7 young and 7 old mice). Data are presented as mean values ± s.e.m. Box-and-whisker plots show the median, the maximum, the minimum and the 25th and 75th percentiles. Exact *P* values are shown above bar plots. Statistics and rest of *P* values are shown in Source data. Source Data

A decline in mitochondrial function has been associated with chronic TCR stimulation and T cell aging^20,21^. We analyzed publicly available scRNA-seq datasets^14^ applying Gene Set Enrichment Analysis and found a downregulation of mitochondrial function-related pathways in CD4^+^ CTLs (Extended Data Fig. 5e and Source Data).

**Fig. 3:**
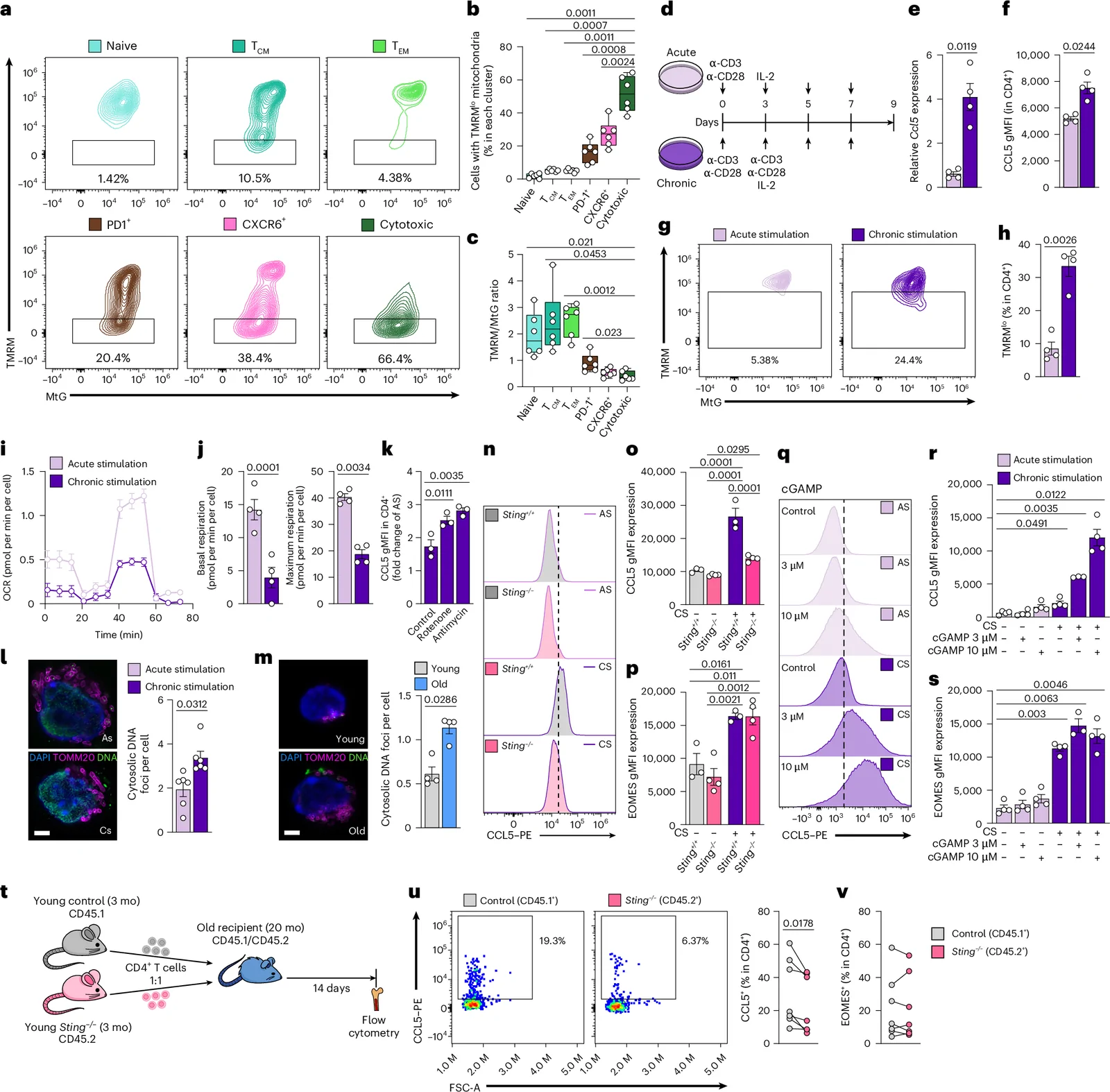
Age-associated mitochondrial stress drives CCL5 production in CD4^+^ CTLs through STING. **a**, Representative contour plots showing TMRM and MtG in the clusters identified by extracellular staining in BM CD4^+^ cells from old (24 mo) mice. **b**, Quantification of the percentage of BM T cells exhibiting TMRM^lo^ staining in the different clusters (*n* = 6 old mice). **c**, Quantification of the TMRM/MtG ratio in the different clusters (*n* = 6 old mice). **d**, Schematic diagram depicting the protocol followed to acutely (AS) and chronically stimulate (CS) isolated CD4^+^ T cells in culture by exposure to anti-CD3/anti-CD28 and IL-2. **e**,**f**, Levels of *Ccl5* mRNA (**e**) and CCL5 protein (**f**) in AS and CS T cells determined by qPCR with reverse transcription analysis and flow cytometry, respectively (*n* = 4 independent cultures). **g**,**h**, Representative contour plots showing TMRM and MtG (**g**) and quantification of the percentage of CD4^+^ cells exhibiting TMRM^lo^ staining (**h**) in AS versus CS conditions (*n* = 4 independent cultures). **i**,**j**, Mitochondrial respiration reflected by oxygen consumption rate (OCR) in AS and CS CD4^+^ T cells under basal conditions or following the addition of oligomycin, the mitochondrial uncoupler BAM15 or the complex I inhibitor rotenone (**i**), and quantification of the maximal and basal respiratory capacities (**j**) (*n* = 4 independent cultures). **k**, Quantification of the levels of CCL5 (fold of AS) in CS CD4^+^ cells treated with rotenone (0.4 µM) or antimycin A (0.4 µM) as indicated (*n* = 3 independent cultures). **l**,**m**, Representative stimulated emission depletion microscopy images (left) and quantification (right) showing DNA foci (green), TOMM20 (magenta) and nuclei (DAPI, blue) in AS and CS CD4^+^ cells (**l**) or CD4^+^ cells isolated from young (3 mo) and old (24 mo) (**m**) mice (*n* = 6 independent cultures for AS versus CS and *n* = 4 independent cultures for young versus old). Scale bar: 2 µm. **n**–**p**, Representative histogram of CCL5 levels (**n**) and quantification of CCL5 (**o**) and EOMES (**p**) gMFI in AS and CS CD4^+^ cells from control and *Sting*^*−/−*^ mice, (*n* = 3 (*Sting*^*+/+*^ CS) or *n* = 4 (remaining conditions) independent cultures). **q**–**s**, Representative histogram of CCL5 levels (**q**) and quantification of CCL5 (**r**) and EOMES (**s**) gMFI in AS and CS CD4^+^ cells treated with vehicle or cGAMP as indicated (*n* = 3 (cGAMP 3 μM CS) or *n* = 4 (remaining conditions) independent cultures). *P* values indicate comparisons between AS-non treated and all CS conditions. All other statistical comparisons are provided in the Source Data. **t**, Schematic diagram depicting the mixed adoptive transfer of CD45.1^+^ control and CD45.2^+^ *Sting*^*−/−*^-derived cells into old (24 mo) CD45.1.2^+^ recipients. **u**,**v**, Representative dot plots and quantification of the percentage of BM CCL5^+^CD4^+^ cells (**u**) or EOMES^+^CD4^+^ cells (**v**) in either CD45.1^+^ control or CD45.2^+^ *Sting*^*−/−*^ mice 21 days after the adoptive transfer (*n* = 7 recipient mice). Data are presented as mean ± s.e.m. Box-and-whisker plots show the median, the maximum, the minimum and the 25th and 75th percentiles. Exact *P* values are shown above bar plots. Statistics and remaining *P* values are shown in the Source Data. gMFI, geometric mean fluorescence intensity. Source Data

Using the MitoTracker Green probe (MtG), which stains mitochondrial mass, in combination with tetramethylrhodamine, methyl ester (TMRM), which depends on the mitochondrial membrane potential, we evaluated the mitochondrial status in the different T cell subsets in the BM. To overcome the impossibility of fixing the MtG probe, we set up a panel of antibodies against extracellular markers to identify the different clusters of BM T cells, and CD4^+^ CTLs were identified as CD44^lo^CD62L^−^CD38^hi^ (Extended Data Fig. 5b). Using this strategy, we observed similar proportions of the different clusters in the aged BM to that observed by unbiased clustering, with a remarkable accumulation of CD4^+^ CTLs (Extended Data Fig. 5c). Intracellular staining for CCL5 confirmed that the majority of CCL5^+^ cells were included in the population identified as cytotoxic by the extracellular strategy (Extended Data Fig. 5d–f and Source Data). This strategy reliably identified CD4^+^ CTLs, as evidenced by the levels of PD-1 and EOMES (Extended Data Fig. 5g and Source Data). Importantly, TMRM and MtG staining confirmed mitochondrial depolarization in CD4^+^ T cells from aged mice (Extended Data Fig. 5h). Analysis in the different clusters, identified by extracellular staining, showed that CD4^+^ CTLs exhibit increased proportions of TMRM^lo^ cells and a decreased TMRM/MtG ratio (Fig. 3a–c and Source Data). Analysis of mitochondrial reactive oxygen species using MitoSOX demonstrated increased levels in CD4^+^ cells from aged mice (Extended Data Fig. 5i), particularly in CD4^+^ CTLs (Extended Data Fig. 5j).

**Extended Data Fig. 6:**
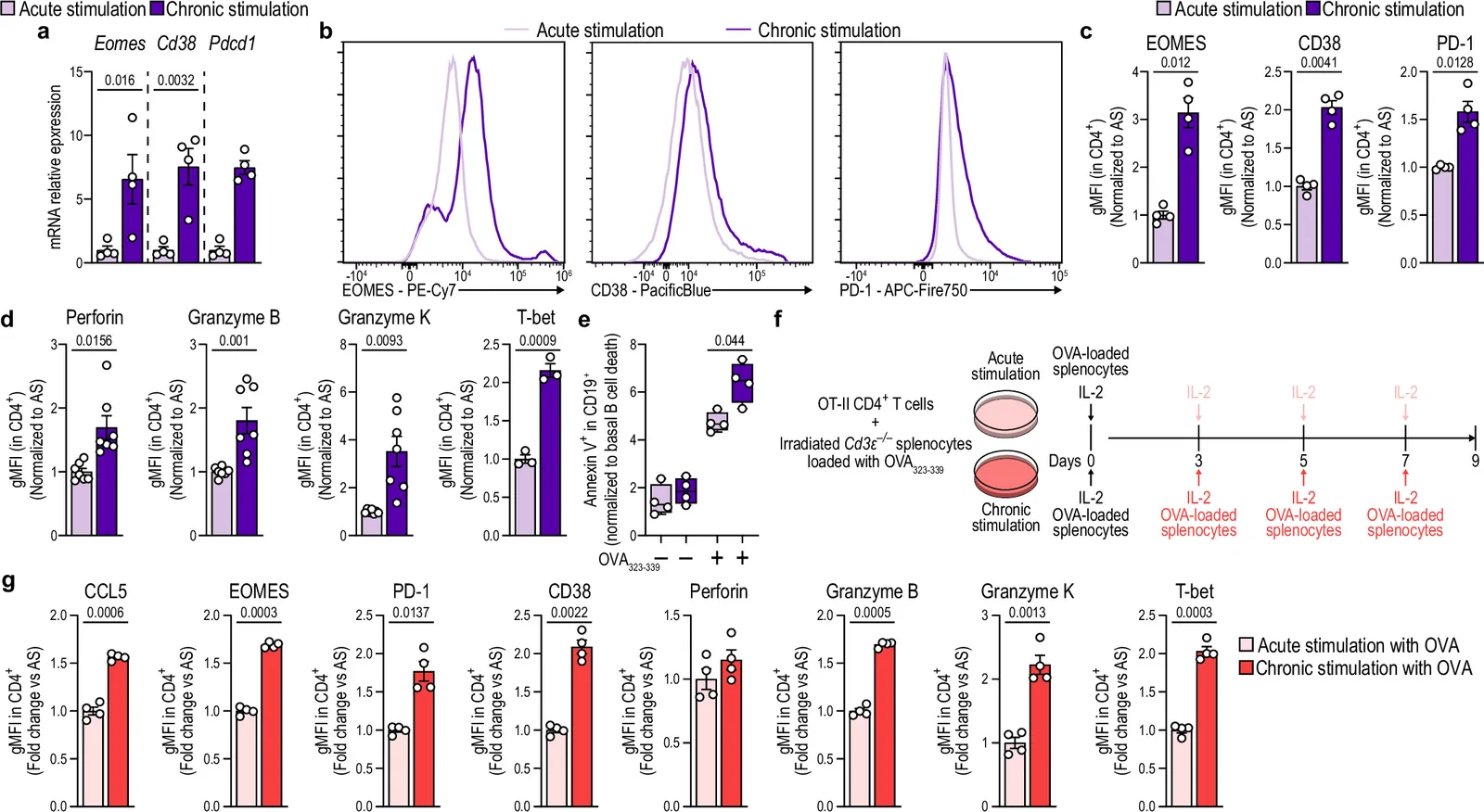
Prolonged TCR stimulation induces the expression of CD4^+^CTL markers in vitro. **a-c**, mRNA (**a**) and protein (**b,c**) levels of EOMES, CD38 and PD-1 in T cells acutely (AS) or chronically stimulated (CS) in vitro with anti-CD3/anti-CD28 determined by RT-qPCR analysis and flow cytometry, respectively (n = 4 independent cultures). **d**, gMFI of Perforin, GzmB, GzmK and T-bet in CD4^+^ cells AS or CS with anti-CD3/anti-CD28 (n = 3 (T-bet) or 7 (rest) independent cultures). **e**, Percentage of Annexin V^+^ B cells in cytotoxicity assays of co-cultures of OT-II cells under chronic anti-CD3/anti-CD28 and purified B cells from mice (n = 4 independent cultures). **f**, Schematic diagram depicting the protocol followed to AS and CS isolated CD4^+^ T cells by antigenic stimulation in co-cultures of *Cd3e*^*−/−*^ splenocytes presenting the OVA_323-339_ peptide and CD4^+^ cells isolated from OT-II mice. **g**, Fold increase of gMFI of CCL5, EOMES, PD-1, CD38, Perforin, GzmB, GzmK and T-bet in OT-II CD4^+^ cells AS or CS with OVA_323-339_ peptide (n = 4 independent cultures). Data are presented as mean values ± s.e.m. Exact P values are shown above bar plots. Statistics and rest of *P* values are shown in Source data. gMFI: Geometric Mean Fluorescence Intensity. Source Data

To assess whether TCR stimulation drives CD4^+^ CTL differentiation and mitochondrial stress, we established an in vitro model using acute (AS) and chronic (CS) anti-CD3/CD28 stimulation (Fig. 3d). CS cells displayed increased messenger RNA and protein levels of CCL5 (Fig. 3e,f), CD38, PD-1 and EOMES (Extended Data Fig. 6a–c). CS cells also exhibited increased levels of T-bet, perforin, GzmB and granzyme K (GzmK) (Extended Data Fig. 6d). Cytotoxic assays on CS cells from OT-II mice co-cultured with isolated B cells loaded with OVA_323–339_ peptide–immunoglobulin M (IgM)-conjugated microbeads, confirmed the functional cytotoxic capacity of CS cells (Extended Data Fig. 6e). In addition, to address whether these features occur upon antigen recognition, we established a protocol of continuous antigenic stimulation (AgCS) by co-culturing CD4^+^ cells from OT-II mice with splenocytes obtained from T cell-deficient mice and loaded with the OVA_323–339_ peptide (Extended Data Fig. 6f). AgCS OT-II cells showed increased levels of CCL5, EOMES, PD-1, CD38 and T-bet together with cytotoxicity-associated proteins such as perforin, GzmB or GzmK (Extended Data Fig. 6g). Notably, CS cells harbored depolarized mitochondria, as evidenced by reduced TMRM and MtG staining (Fig. 3g,h), and showed impaired mitochondrial respiration (Fig. 3i,j and Source Data). These data suggest that chronic TCR stimulation drives cytotoxic differentiation and mitochondrial stress in CD4^+^ cells.

Given that mitochondrial stress triggers the production of inflammatory mediators^22^, we investigated whether it also contributes to the induction of CCL5 in CD4^+^ CTLs. Inhibition of mitochondrial complexes I or III with either rotenone or antimycin A increased CCL5 levels in CS cells (Fig. 3k). Mitochondrial stress is known to activate the cGAS–STING pathway through cytosolic release of mitochondrial DNA^23,24^. Consistent with this, we detected cytosolic DNA foci without TOMM20 co-localization in aged CD4^+^ T cells and in CS cells (Fig. 3l,m). CD4^+^ CS from *Sting*^*−/−*^ mice exhibited markedly reduced CCL5 levels despite preserved EOMES expression (Fig. 3n–p), suggesting that STING is dispensable for cytotoxic differentiation but required for CCL5 induction. Conversely, STING activation with cyclic GMP–AMP (cGAMP) increased CCL5 levels in a dose-dependent manner without altering the levels of EOMES (Fig. 3q–s and Source Data).

To test the relevance of this pathway in vivo, we adoptively transferred CD45.1^+^*Sting*^*+/+*^ and CD45.2^+^*Sting*^*−/−*^ CD4^+^ T cells (in a 1:1 ratio) into aged CD45.1.2^+^ recipients (Fig. 3t). Twenty-one days later, in the BM, we detected lower percentages of CCL5^+^ in CD45.2^+^*Sting*^*−/−*^ compared to CD45.1^+^*Sting*^*+/+*^CD4^+^ T cells (Fig. 3u), whereas EOMES^+^ cell frequencies were comparable (Fig. 3v).

Collectively, these results indicate that CD4^+^ CTLs exhibiting mitochondrial stress accumulate in the aged BM, showing increased CCL5 expression in a STING-dependent manner.

**Extended Data Fig. 7:**
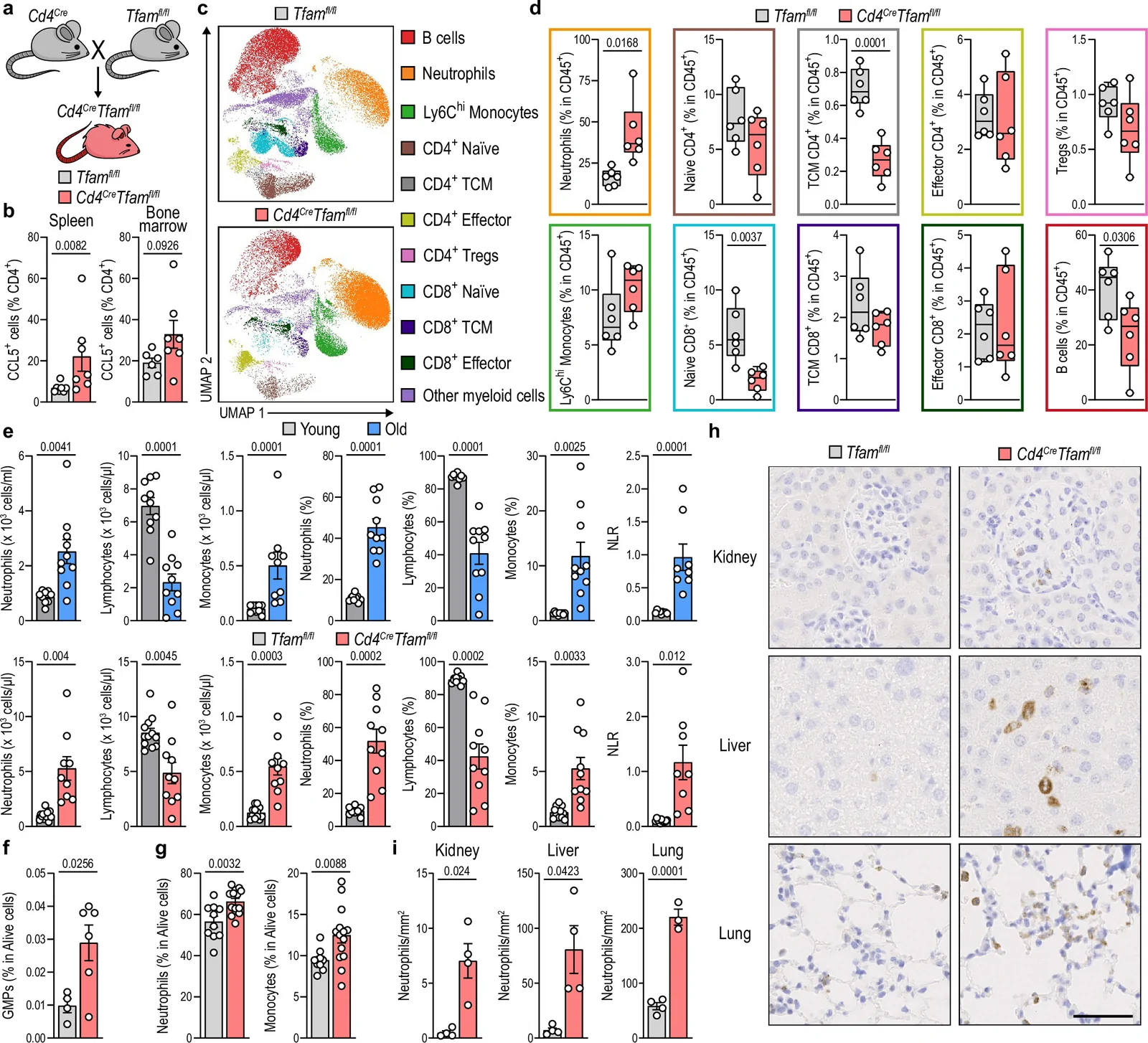
Genetic induction of a mitochondrial dysfunction in T cells enhances myelopoiesis and neutrophil output in mice. **a**, Diagram showing the genetic strategy followed to generate *CD4*^*Cre*^ *Tfam*^*fl/fl*^ mice. **b**, Percentage of splenic CCL5^+^CD4^+^ cells in *Tfam*^*fl/fl*^ and *CD4*^*Cre*^*Tfam*^*fl/fl*^ mice (n = 6 *Tfam*^*fl/fl*^ and 7 *CD4*^*Cre*^*Tfam*^*fl/fl*^ mice). **c**, Representative UMAP with Cluster-X overlay showing the distribution of the eleven clusters of immune cells identified by multiparametric spectral flow cytometry in the circulation of *Tfam*^*fl/fl*^ (8 months old, mo) and *CD4*^*Cre*^*Tfam*^*fl/fl*^ (8 mo) mice. **d**, Boxplots comparing the percentage of the different depicted populations in *Tfam*^*fl/fl*^ and *CD4*^*Cre*^*Tfam*^*fl/fl*^ mice (n = 6 *Tfam*^*fl/fl*^ and 6 *CD4*^*Cre*^*Tfam*^*fl/fl*^ mice). **e**, Haemogram analysis of blood from young (3mo) and old (24 mo) mice (**up**) and *Tfam*^*fl/fl*^ and *CD4*^*Cre*^*Tfam*^*fl/fl*^ mice (6mo) mice (**down**) (young vs old; n = 9 (NLR, neutrophils/μL, % neutrophils, % lymphocytes) or 10 (rest) young and 8 (NLR) or 9 (monocytes/μL) or 10 (rest) old mice. *Tfam*^*fl/fl*^ and *CD4*^*Cre*^*Tfam*^*fl/fl*^; n = 11 (NLR, % neutrophils, % lymphocytes) or 12 (rest) *Tfam*^*fl/fl*^ and 8 (NLR) or 9 (neutrophils/μL) or 10 (rest) *CD4*^*Cre*^*Tfam*^*fl/fl*^ mice). **f**, Percentage of GMPs in the BM of *Tfam*^*fl/fl*^ and *CD4*^*Cre*^*Tfam*^*fl/fl*^ mice (n = 4 *Tfam*^*fl/fl*^ and 6 *CD4*^*Cre*^*Tfam*^*fl/fl*^ mice). **g**, Percentage of neutrophils and Ly6C^hi^ monocytes in the BM of *Tfam*^*fl/fl*^ and *CD4*^*Cre*^*Tfam*^*fl/fl*^ mice (n = 9 (monocytes) or 10 (neutrophils) *Tfam*^*fl/fl*^ and 14 *CD4*^*Cre*^*Tfam*^*fl/fl*^ mice). **h**, Immunohistochemistry for MPO in kidney, liver and lung sections from *Tfam*^*fl/fl*^ and *CD4*^*Cre*^*Tfam*^*fl/fl*^ mice. Scale bar: 50 μm. **I**, Quantification of MPO-positive neutrophils per area (n = 4 *Tfam*^*fl/fl*^ and 4 *CD4*^*Cre*^*Tfam*^*fl/fl*^ mice). Data are presented as mean values ± s.e.m. Box-and-whisker plots show the median, the maximum, the minimum and the 25th and 75th percentiles. Exact *P* values are shown above bar plots. Statistics and rest of *P* values are shown in Source data. Source Data

In light of the accumulation of depolarized mitochondria in CD4^+^ CTLs, we investigated whether a deteriorated mitochondrial function in CD4^+^ T cells was sufficient to induce CTL conversion and myelopoiesis skewing. To this end, we generated mice with a T cell-specific deletion of the mitochondrial transcription factor A (*Tfam*) gene by crossing *Tfam*^*fl/fl*^ mice with mice expressing the Cre recombinase under the *Cd4* promoter (*Cd4*^*Cre*^) (Extended Data Fig. 7a). T cells from *Cd4*^*Cre*^*Tfam*^*fl/fl*^ mice display an activated phenotype^25,26^, inducing the expression of inflammaging-associated cytokines that trigger paracrine senescence and age-associated multimorbidity^26^. At 8 months old, *Cd4*^*Cre*^*Tfam*^*fl/fl*^ mice presented an accumulation of CD4^+^CCL5^+^ cells comparable to that observed in 24-month-old mice (Fig. 2c and Extended Data Fig. 7b). Analysis of circulating immune cell populations showed a remarkable accumulation of neutrophils together with a decrease in T and B cells (Extended Data Fig. 7c,d). This was confirmed by hemogram analysis and correlated with a concomitantly increased NLR, mirroring observations in old mice (Extended Data Fig. 7e). Supporting the idea that enhanced myelopoiesis contributes to the increased levels of circulatory neutrophils, we detected an increased percentage of GMPs in the BM of *Cd4*^*Cre*^*Tfam*^*fl/fl*^ mice (Extended Data Fig. 7f). *Cd4*^*Cre*^*Tfam*^*fl/fl*^ mice also presented increased numbers of splenic neutrophils and monocytes (Extended Data Fig. 7g) together with infiltrating myeloperoxidase-positive (MPO) neutrophils in the kidney, liver and lung (Extended Data Fig. 7h,i). Taken together, these findings suggest that genetic induction of mitochondrial dysfunction in T cells is sufficient to promote CD4^+^ CTL differentiation and induce myelopoiesis, recapitulating the phenotype seen in naturally aged mice.

### Aged CD4^+^ CTLs induce myelopoiesis through CCL5–CCR5 signaling

**Fig. 4:**
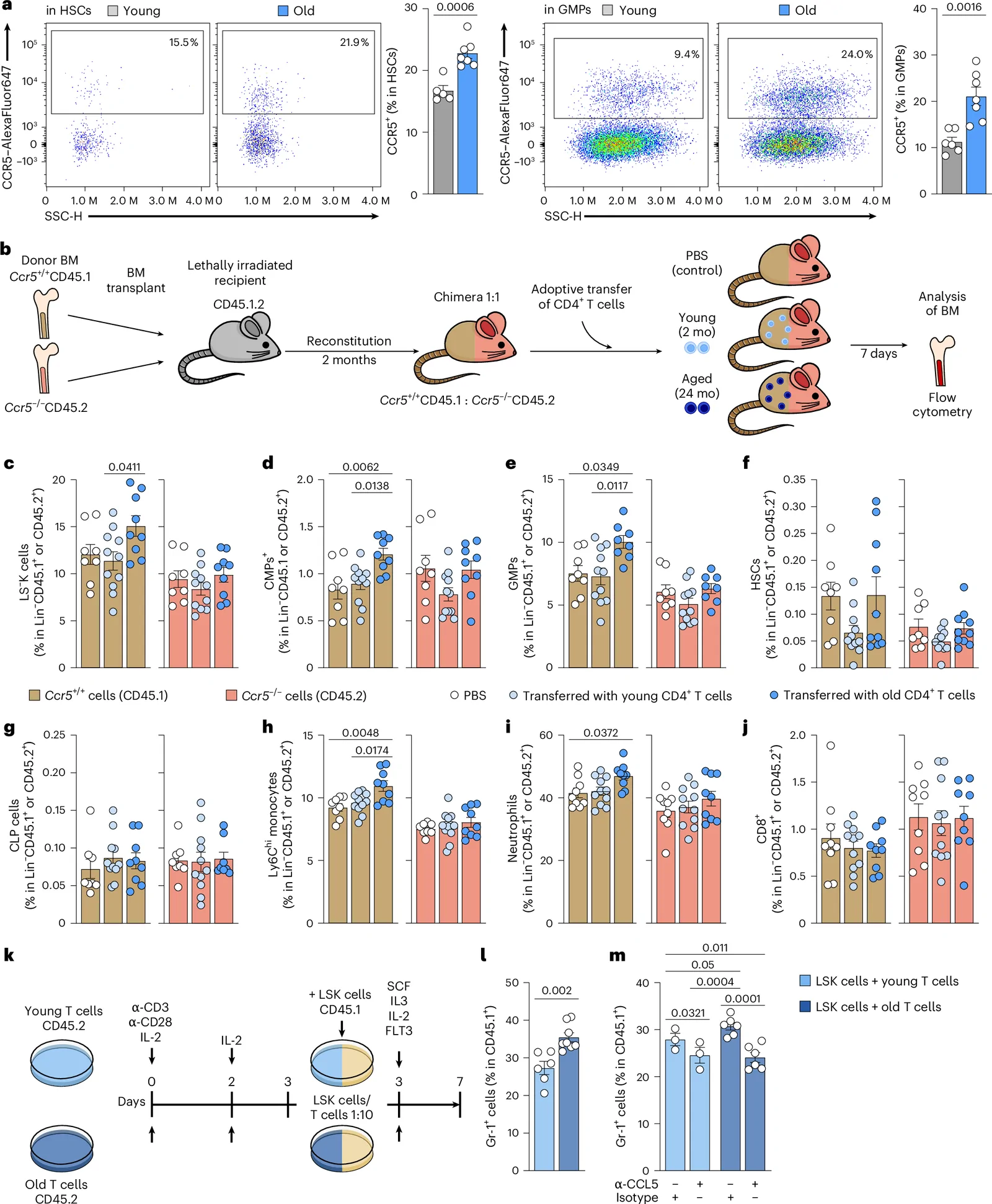
Aging-associated CD4^+^ T cells induce myelopoiesis via CCL5–CCR5. **a**, Representative dot plots showing the identification of CCR5^+^ HSCs and GMPs, and quantification of the percentage of CCR5^+^ HSCs and CCR5^+^ GMPs in the BM of young (3 mo) and old (24 mo) mice (*n* = 5 young (HSCs) or *n* = 6 young (GMPs) and *n* = 7 old mice). **b**, Schematic diagram depicting the strategy followed to generate mixed BM chimeric mice. CCR5^+/+^ (CD45.1^+^) and CCR5^*−*/*−*^ (CD45.2^+^) derived BM cells were mixed 1:1 and injected intravenously into lethally irradiated young (5–6 mo) CD45.1.2^+^ recipients. Two months after transplantation, either PBS or CD4^+^CD45.2^+^ isolated from young (3 mo) or old (24 mo) mice were adoptively transferred into the mixed chimeric recipients. **c**–**g**, Quantification of the percentage of LS^*−*^K (**c**), CMPs (**d**), GMPs (**e**), HSCs (**f**) and CLPs (**g**) in Lin^−^CCR5^+/+^CD45.1^+^ (left) or Lin^*−*^CCR5^*−*/*−*^CD45.2^+^ (right) cells from the BM of mixed chimeric recipients treated with PBS or transferred with CD4^+^ cells from young or old mice as indicated (*n* = 8 PBS, *n* = 11 young-transferred and *n* = 9 old-transferred recipient chimeras). **h**–**j**, Quantification of the percentage of Ly6C^hi^ monocytes (**h**), neutrophils (**i**) and CD8^+^ cells (**j**) in CCR5^+/+^CD45.1^+^ (left) or CCR5^*−*/*−*^CD45.2^+^ (right) cells from the BM of mixed chimeric recipients treated with PBS or transferred with CD4^+^ cells from young or old mice as indicated (*n* = 8 PBS (monocytes), *n* = 9 PBS (rest), *n* = 11 young-transferred and *n* = 9 old-transferred recipient chimeras). **k**, Schematic representation showing the in vitro differentiation of LSK cells isolated from CD45.1^+^ mice co-cultured with CD4^+^ cells isolated from young (3 mo) or old (24 mo) mice in a 1:10 ratio (1 T cell to 10 LSK cells). **l**, Quantification of the percentage of Gr-1^+^ progeny cells in LSK cells after 7 days of co-culture (each dot represents an independent culture polling LSK cells from two or three mice). **m**, Quantification of Gr-1^+^ cell percentage in LSK cells after 7 days of co-culture either exposed to CCL5 blocking or isotype antibodies as indicated (each dot represents an independent culture polling LSK cells from two or three mice). Data are presented as mean values ± s.e.m. Exact *P* values are shown above bar plots. Statistics and remaining *P* values are shown in the Source Data. Source Data

Given that signaling through CCL5 and CCR5 has been involved in the induction of myeloid skewing in mouse models of multiple sclerosis^11,27^, we studied the contribution of this pathway to T cell-induced myelopoiesis during aging. Notably, we initially observed that HSCs and GMPs from old mice exhibit an upregulation of CCR5, the main CCL5 receptor, leading to increased percentages of CCR5^+^ HSCs and GMPs in the BM of old mice (Fig. 4a). To explore the relevance of signaling through CCL5–CCR5 in T cell-induced myelopoiesis during aging, we generated mixed BM chimeric mice by injecting 50% of CD45.1*Ccr5*^*+/+*^ and 50% of CD45.2*Ccr5*^*−/−*^ BM cells into irradiated CD45.1.2 recipients. Two months later, we compared chimeric mice treated with phosphate-buffered saline (PBS) or adoptively transferred with CD4^+^ cells from either young or aged donor mice, and we evaluated the potential of the transferred cells to induce myelopoiesis in both, *Ccr5*^*+/+*^ and *Ccr5*^*−/−*^, progenitors one week later (Fig. 4b). Flow cytometry analysis revealed that CD45.1^+^*Ccr5*^*+/+*^ cells responded to CD4^+^ cells from old mice, but not to young CD4^+^ cells or PBS, by increasing the percentage of LS^−^K cells, CMPs and GMPs (Fig. 4c–e and Source Data) in the BM. By contrast, HSCs (Fig. 4f) or CLPs (Fig. 4g) remained constant, altogether suggesting myeloid bias upon aged CD4^+^ cell transfer. Of note, upon transfer of CD4^+^ cells from old donors, CD45.2^+^*Ccr5*^*−/−*^ cells maintained basal levels of LS^−^K cells, CMPs, GMPs (Fig. 4c–e), HSCs (Fig. 4f and Source Data) and CLPs (Fig. 4g and Source Data). Accordingly, we observed increased percentages of CD45.1^+^*Ccr5*^*+/+*^, but not of CD45.2^+^*Ccr5*^*−/−*^, Ly6C^hi^ monocytes and neutrophils upon transfer of CD4^+^ cells from old mice in the BM (Fig. 4h,i and Source Data). Supporting a selective effect on the myeloid lineage, we did not find alterations in the percentage of either CD45.1^+^*Ccr5*^*+/+*^ or CD45.2^+^*Ccr5*^*−/−*^CD8^+^ cells upon transfer of CD4^+^ cells from old mice (Fig. 4j and Source Data). Taken together, these data suggest that the induction of myelopoiesis by aged CD4^+^ cells requires CCR5 signaling.

Finally, to address whether CD4^+^ cells can directly induce myelopoiesis, we co-cultured CD45.1^+^ Lin^−^Sca-1^+^c-Kit^+^ (LSK) cells with CD45.2^+^CD4^+^ T cells isolated from either young or old mice (Fig. 4k) and analyzed the percentage of CD45.1^+^GR-1^+^ cells, a myeloid cell marker. Direct exposure to CD4^+^ T cells from aged mice increased differentiation of LSK cells toward the myeloid lineage, confirming observations in mice (Fig. 4l). Furthermore, this was reverted by addition of an anti-CCL5 blocking antibody in the culture medium (Fig. 4m and Source Data). These results suggest that CD4^+^ cells from old mice directly induce myelopoiesis by secreting CCL5.

In aggregate, these findings suggest that CCL5–CCR5 signaling in HSCs and downstream progenitors is involved in aged T cell-induced myelopoiesis in mice.

### Inhibition of CCR5 reduces myelopoiesis, decreases the NLR and improves health status in aged mice

**Fig. 5:**
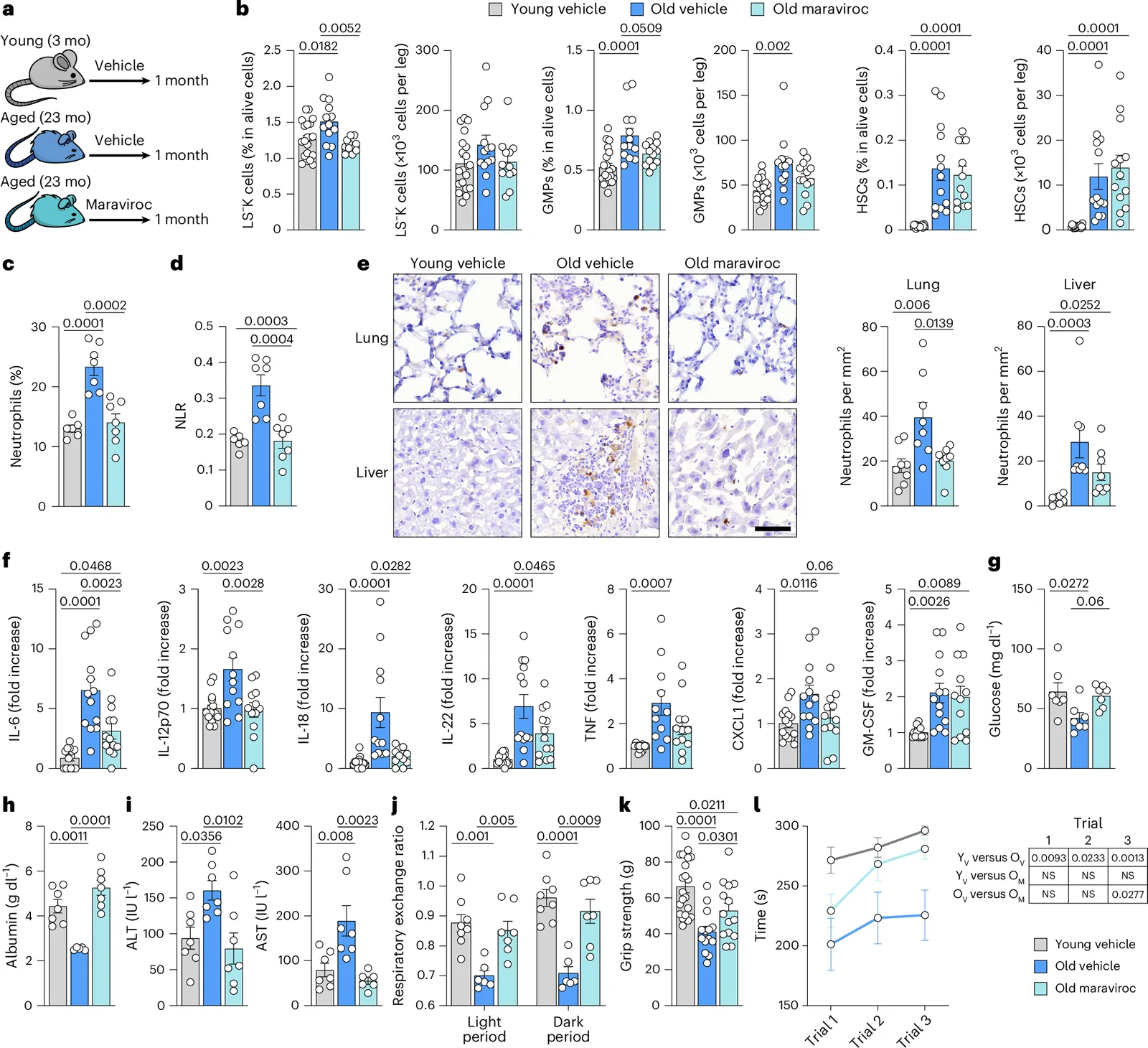
Pharmacological targeting of CCR5 reverses myeloid skewing and functional decline in old mice. **a**, Schematic diagram detailing the treatment with maraviroc. Old mice were treated daily with either vehicle or maraviroc for one month. Young mice were treated with vehicle in parallel. **b**, Percentage and numbers of GMPs, LS^−^K cells and HSCs in the BM of the three experimental groups (*n* = 20 (% LS^−^K cells, GMPs and HSCs) or *n* = 19 young vehicle (total cells per leg LS^−^K cells, GMPs and HSCs); *n* = 12 old vehicle (cells per leg GMPs) or *n* = 13 old vehicle (rest); and *n* = 11 old maraviroc (% LS^−^K cells), *n* = 12 old maraviroc (% HSCs or total cells per leg of LS^*−*^K cells) or *n* = 13 old maraviroc (% GMPs and total cells per leg of GMPs and HSCs) treated mice). **c**, Percentage of blood neutrophils in the experimental groups measured by blood hematology (*n* = 6 young, *n* = 7 old vehicle-treated and *n* = 7 old maraviroc-treated male mice). **d**, NLR in blood from the experimental groups (*n* = 6 young, *n* = 7 old vehicle-treated and *n* = 7 old maraviroc-treated male mice). **e**, MPO immunohistochemistry and quantification of MPO^+^ cells per mm^2^ in liver and lung sections from all experimental groups (*n* = 7 young (liver) or *n* = 8 young (lung), *n* = 8 old vehicle-treated and *n* = 8 old maraviroc-treated male mice). Scale bar: 50 μm. **f**, Serum levels of the indicated cytokines and chemokines detected by multiplex in the three experimental groups (*n* = 14 (IL-6, tumor necrosis factor (TNF)) or *n* = 15 (IL-12p70, IL-18, IL-22, CXCL1, GM-CSF) young mice; *n* = 11 (TNF), *n* = 12 (IL-12p70, IL-18) or *n* = 13 (IL-6, IL-22, CXCL1, GM-CSF) old vehicle-treated mice; and *n* = 11 (IL-18), *n* = 12 (IL-12p70, TNF, CXL1, GM-CSF) or *n* = 13 (IL-6, IL-22) old maraviroc-treated mice). **g**, Serum glucose levels in nonfasted mice from the experimental groups (*n* = 7 young vehicle, *n* = 7 old vehicle and *n* = 7 old maraviroc-treated male mice). **h**, Serum albumin levels in nonfasted mice from the experimental groups (*n* = 7 young vehicle, *n* = 5 old vehicle and *n* = 7 old maraviroc-treated male mice per group). **i**, Serum ALT and AST levels in nonfasted mice from the experimental groups (*n* = 7 young vehicle, *n* = 7 old vehicle and *n* = 6 (AST) or *n* = 7 (ALT) old maraviroc-treated mice). **j**, Respiratory exchange ratio (VCO_2_/VO_2_) assessed in metabolic cages during the light and dark phases among the specified experimental groups (*n* = 8 young vehicle, *n* = 6 old vehicle and *n* = 7 old maraviroc-treated mice). **k**, Quantification of forelimb strength in the three experimental groups (*n* = 21 young vehicle, *n* = 14 old vehicle and *n* = 14 old maraviroc-treated mice). **l**, Quantification of the latency to fall in the rotarod test in the three experimental groups (*n* = 13 young vehicle, *n* = 12 old vehicle and *n* = 8 old maraviroc-treated mice). Numbers in the table reflect *P* values of all statistical comparisons. Data are presented as mean ± s.e.m. Exact *P* values are shown above bar plots. Statistics and remaining *P* values are shown in the Source Data. NS, not significant; O_M_, old maraviroc; O_V_, old vehicle; Y_V_, young vehicle. Source Data

Recent studies demonstrated the potential of supressing myelopoiesis to improve age-associated immune system deterioration^2^. To investigate whether interfering with CCR5 signaling could prevent age-associated changes in hematopoiesis, we treated old mice for one month with maraviroc, a Food and Drug Administration (FDA)-approved CCR5 antagonist^28^ (Fig. 5a). Treatment with maraviroc decreased the percentage of GMPs and LS^−^K cells, with no effects on HSCs (Fig. 5b and Extended Data Fig. 2), confirming the reduction of myelopoiesis by CCR5 inhibition. Accordingly, treatment with maraviroc decreased the percentage of circulating neutrophils (Fig. 5c), leading to a concomitant normalization of the NLR (Fig. 5d) in old mice. These results suggest that CCR5 inhibition turns hematopoiesis into a more youthful state.

**Extended Data Fig. 8:**
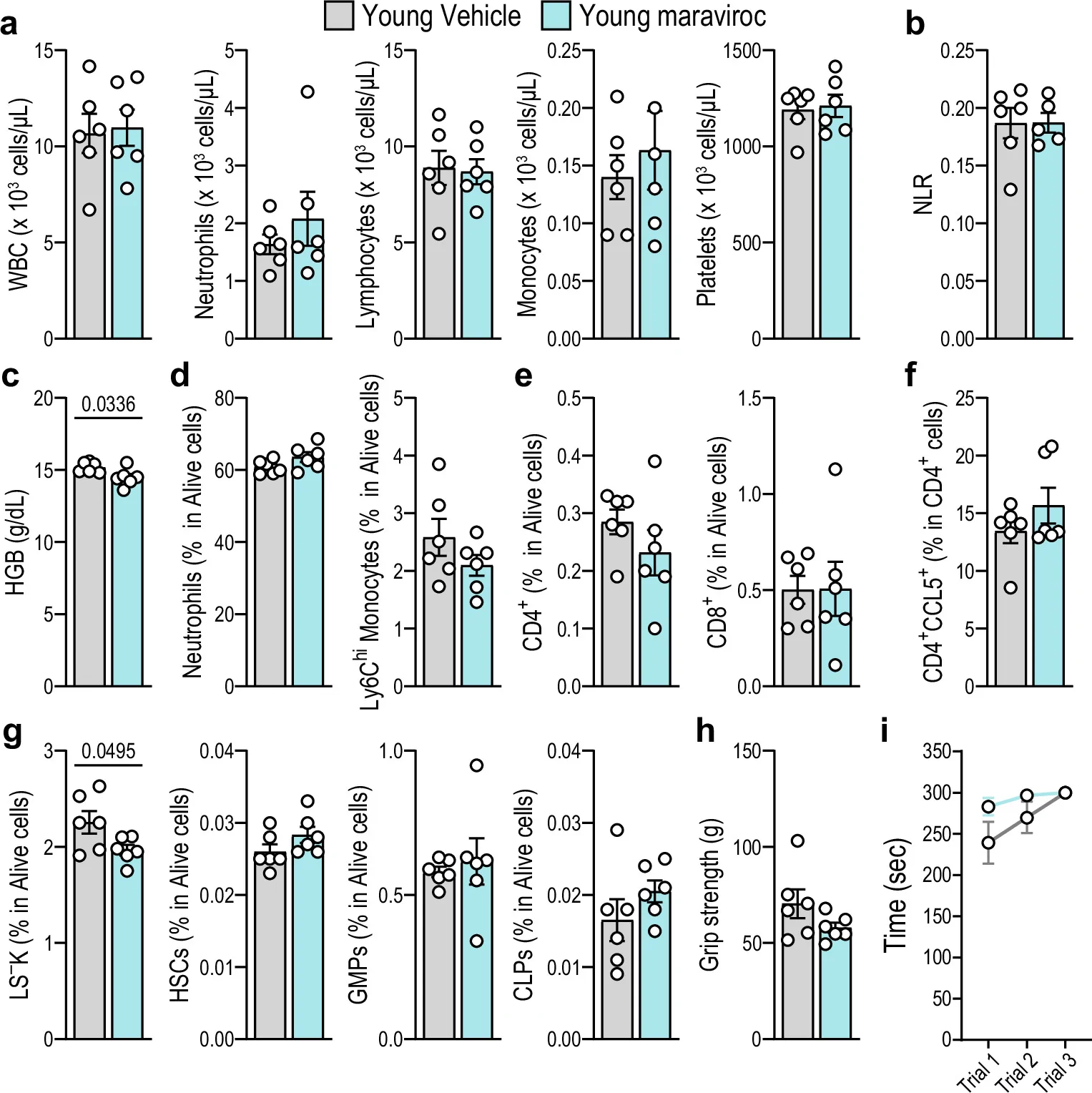
Pharmacological inhibition of CCR5 in young mice. **a**, Circulating levels of white blood cells (WBC), neutrophils, lymphocytes, monocytes and platelets in young mice treated with vehicle or maraviroc for one month (n = 6 young vehicle and 5 (monocytes) or 6 (rest) young maraviroc treated mice). **b**,**c**, NLR (**b**) and haemoglobin levels (**c**) in the same set of mice (n = 6 young vehicle and 5 (NLR) or 6 (HGB) young maraviroc treated mice). **d**,**e**, levels of neutrophils, Ly6C^hi^ monocytes (**d**) and CD4^+^, CD8^+^ cells (**e**) in the bone marrow (n = 6 young vehicle and 6 young maraviroc treated mice). **f**, Percentage of CCL5^+^CD4^+^ cells in the bone marrow from the same set of mice (n = 6 young vehicle and 6 young maraviroc treated mice). **g**, Levels of LS^−^K, HSCs, GMPs, and CLPs in the bone marrow (n = 6 young vehicle and 6 young maraviroc treated mice). **h**, Quantification of forelimbs strength in the same set of mice (n = 6 young vehicle and 6 young maraviroc treated mice). **i**, Quantification of the latency to fall in the rotarod test (n = 6 young vehicle and 6 young maraviroc treated mice). Data are presented as mean values ± s.e.m. Exact *P* values are shown above bar plots. Statistics and rest of *P* values are shown in Source data. Source Data

We next studied whether treatment with maraviroc improves health outcomes in old mice. Old mice treated with maraviroc exhibited reduced levels of infiltrating neutrophils in lung and liver (Fig. 5e). Furthermore, maraviroc lowered the serum levels of multiple inflammaging-associated cytokines, without affecting granulocyte–macrophage colony-stimulating factor (GM-CSF) (Fig. 5f), as well as other aging markers such as nonfasting glucose (Fig. 5g), albumin (Fig. 5h), alanine aminotransferase (ALT) and aspartate aminotransferase (AST) (Fig. 5i). Remarkably, this correlated with normalization of the different physiological parameters affected in old mice including metabolic alterations such as the respiratory exchange ratio (Fig. 5j), loss of muscle strength (Fig. 5k) and impaired locomotor coordination (Fig. 5l). Notably, treatment with maraviroc did not have significant effects on young mice (Extended Data Fig. 8a–i). Taken together, these results suggest that CCR5 inhibition by maraviroc can suppress age-associated myelopoiesis induction in mice and improve health outcomes in old mice.

## Discussion

CD4^+^ CTLs are emerging as age-associated T cells because their numbers correlate with aging^14,17^. However, their origin and contribution to aging are still debated. Previous findings reported the presence of these cells mainly in conditions characterized by continuous TCR stimulation such as viral infections^29^. We found that young T cells exposed to an aged environment differentiate into CD4^+^ CTLs, requiring TCR activation. It is tempting to speculate that an altered repertoire of presented antigens could be the origin of CD4^+^ CTL differentiation in an aged environment. Regarding their function, CD4^+^ CTLs are responsible for eliminating senescent cells in the skin^30^ and may be involved in the control of tumor growth^31,32^. Despite these functions, massive accumulation of CD4^+^ CTLs in the BM during aging, due to either enhanced migration or delayed clearance, would result in an increase in myelopoiesis and neutrophilia.

A skewing of hematopoiesis toward the production of myeloid cells is an aging feature with a major impact on immune system function, tissue homeostasis and healthspan. Even though a high NLR is associated with bad prognoses in most age-associated diseases^4–7^, the mechanisms driving altered hematopoiesis during aging remain incompletely understood. Previous findings have established that remodeling of the BM stroma induces the skewing of hematopoiesis in aged mice. Accordingly, inflammation triggered by β2 or β3 adrenergic receptors induces hematopoiesis skewing^1,33^, at least in part, by increasing interleukin 1β (IL-1β) levels in aged mice^34^. Indeed, similar mechanisms are exploited by tumors to corrupt myeloid cells of the BM stroma in both young and aged mice^35,36^. Likewise, decreased levels of trophic factors such as insulin-like growth factor 1 (ref. ^37^) or an altered cellular composition of the BM niche, including a Notch-dependent reduction of blood vessels^38^ or an accumulation of adipocytes^39^, alter the fate of hematopoietic precursors. Our findings suggest that infiltration of CD4^+^ CTLs, into the BM during aging will be a critical factor contributing to the remodeling of the BM niche. These cells, characterized by an overexpression of CCL5, induce myelopoiesis by signaling to CCR5 in hematopoietic precursors. These observations are in line with previous findings reporting enhanced myelopoiesis and neutrophil generation by BM infiltrating autoreactive CCL5^+^ T cells in multiple sclerosis^11^. Whether CCL5^+^ T cells in multiple sclerosis are bona fide CD4^+^ CTLs, and whether the mechanisms controlling CCL5 expression in aged BM T cells are shared across autoimmune diseases, remains unresolved. Notably, these findings open new avenues to explore whether immune dysfunction during aging and autoimmune diseases may arise from common underlying mechanisms.

We observed that CD4^+^ CTLs exhibit pronounced mitochondrial dysfunction accompanied by cytosolic DNA release. In vitro, activation of the cGAS–STING pathway triggered CCL5 expression, whereas STING deficiency normalized CCL5 levels in CS cells. In vivo, STING deficiency reduced, but did not completely abolish the induction of CCL5 following exposure to an aged environment. Regulation of CCL5 expression is complex and this partial effect suggests that additional pathways may contribute to CCL5 upregulation in CD4^+^ CTLs. In this sense, NF-κB is a well-known activator of CCL5 in different cell types^40^. Moreover, NF-κB can be activated by STING^41^ or, in T cells, directly by TCR activation^42^. Thus, CCL5 expression may be triggered, independently of mitochondrial stress, by chronic TCR activation in CD4^+^ CTLs. In parallel, we found that CD4^+^ CTLs overexpress CD38, a NADase that triggers mitochondrial stress in T cells^43^. Accordingly, inhibition of CD38 enhances mitochondrial fitness and metabolic resilience in chimeric antigen receptor T cells from age mice, improving their anti-tumor effectiveness^44^. Future studies will be required to dissect the contribution of STING-independent pathways to the induction of mitochondrial stress and CCL5 in CD4^+^ CTLs and to explore whether strategies that improve mitochondrial health can reduce CCL5 levels in these cells.

The accumulation of stressed mitochondria in these cells does not preclude the activation of compensatory mechanisms that may allow them to cope with it, such as increased mitochondrial biogenesis, metabolic rewiring that reduces dependence on mitochondrial function, enhanced mitophagy or upregulation of pro-survival pathways. Indeed, these cells have been reported to expand in supercentenarians^17^ and to retain functional cytotoxic capacity, including the ability to eliminate senescent cells^30,45^. It is therefore plausible that these potentially beneficial functions favor the activation of pathways that promote the survival and persistence of these cells in aged individuals, even at the cost of deleterious side effects.

Remarkably, recent evidence suggests that proinflammatory neutrophils can induce paracrine senescence^46^, trigger age-related lung damage^47^, worsen myocardial infarction^48^ and foster skin inflammation^49^. Importantly, we found that enhanced myelopoiesis in aged mice is prevented by pharmacological inhibition of CCR5, correlating with an improvement in multiple aging-associated conditions. Although a potential contribution from cells other than neutrophils cannot be excluded, our findings suggest that pharmacological inhibition of CCR5 may exert systemic geroprotective effects. Supporting these findings, human individuals carrying homozygous loss-of-function mutations in CCR5 (CCR5-Delta32) present better outcomes after stroke^50^ and during multiple sclerosis^27^. Future studies should investigate whether this improvement is mediated by a decreased induction of myelopoiesis, which contributes to aggravating stroke and autoimmune neuroinflammation in mice^11,51^. Supporting this hypothesis, rebalancing hematopoiesis by depleting myeloid-biased HSCs with a cocktail of antibodies rejuvenates immune function in aged mice^2^. All these findings, together with previous reports demonstrating that immune cells are direct executors of systemic aging^26,52^, suggest that beneficial effects of rebalancing hematopoiesis during aging may extend beyond immune function rejuvenation.

Previous studies identified CCL5 as a driver of myelopoiesis in aged mice^53^. Although our findings highlight a role for CD4^+^ CTLs, they do not rule out the contribution of additional cellular sources of CCL5. Indeed, CCL5 release by monocytes has been reported in aged humans^54^, while CD8^+^ T cells and natural killer cells have also been shown to produce CCL5 in different contexts^55,56^. Therefore, whether CCL5 production by these or other cell populations may amplify the effects of CD4^+^ CTLs in driving age-associated myelopoiesis remains to be determined.

In all, these findings place T cells as inducers of the disbalance in hematopoiesis seen during aging. Given that myeloid-biased hematopoiesis is a hallmark of human aging^3^ and that clonal hematopoiesis of indeterminate potential, a common age-related phenomenon, underlies several age-associated pathologies^57–59^ it is plausible that T cells play a broader role in these processes. Because systemic inflammation and a high NLR predict mortality across diverse age-related diseases^4–7^, and considering that maraviroc is already FDA-approved for human immunodeficiency virus treatment^28^, our findings may have broad therapeutic implications for the treatment of age-related pathologies. Taken together, these findings set the stage for the development of strategies to modulate CD4^+^ CTLs function, avoid their recruitment into the BM, and modify their interplay with hematopoietic precursors to improve age-associated outcomes.

## Methods

All surgical and experimental procedures were approved by the ethics committee of the Consejo Superior de Investigaciones Científicas (CSIC) and authorized by Comunidad de Madrid (PROEX 52.1/23 and PROEX 144.1/24).

### Animal procedures

All mice were bred and aged in the specific-pathogen-free facility of Centro de Biología Molecular Severo Ochoa (CBMSO, Madrid, Spain) in accordance with European Union recommendations and institutional guidelines. C57BL/6J HccRsd mice were purchased from Envigo or bred at the CBMSO animal facilities. *Tfam*^*fl*/*fl*^ mice were kindly provided by N.G. Larsson^60^, and *CD4*^*Cre+*/*wt*^ mice were purchased from the Jackson Laboratory. Double heterozygotes (*Tfam*^*+*/*fl*^, *CD4*^*Cre+*/*wt*^) were obtained and backcrossed to the *Tfam*^*fl*/*fl*^ strain to generate cell-specific knockouts (*CD4*^*Cre*^*Tfam*^*fl*/*fl*^). OT-II mice were kindly provided by N. Martínez-Martín (CBM Severo Ochoa). *Ccr5*^*−*/*−*^ mice were provided by S. Mañes (CNB). *Cd3e*^*−/−*^ mice were kindly donated by B. Alarcón (CBM Severo Ochoa). *Rag1*^*−/−*^ (1547488)^13^, C57BL/6J CD45.1.2 and C57BL/6J CD45.1 mice were provided by C. Cobaleda (CBM Severo Ochoa). Sting-deficient mice (Sting_emKO) were generated and provided by M. Pasparakis (University of Cologne, Germany) and maintained in a C57BL/6N background. The rest of the mice used in this study were maintained in a C57BL/6J background. Most experiments were performed in female mice unless specified otherwise. In general, mice were used at different ages: young (less than 4 months old) and old (22–25 months old) unless specified otherwise.

#### Adoptive transfer of immune cells

For the adoptive transfer of CD4^+^ cells, the spleen and the axillary, mesenteric and inguinal lymph nodes were harvested from either young or old C57BL/6 CD45.1, C57BL/6 CD45.2 or OT-II (CD45.2) donor mice as specified in the figures. Organs were mashed and filtered through a 70-μm cell strainer and erythrocytes were removed in erythrocyte lysis buffer for 5 min. CD4^+^ cells were purified using the MojoSort mouse CD4 T cell isolation kit (BioLegend, cat. no. 480006) according to the manufacturer’s instructions. CD4^+^ cells were resuspended in 0.9% NaCl and 6 million cells were injected intravenously into every young and old C57BL/6 CD45.1, CD45.2 or CD45.1.2 recipient, as specified in the figure captions. The purity of CD4^+^ isolated cells was routinely tested by flow cytometry, typically ranging from 85% to 95% of total cells.

#### BM transplantation

For BM transplantation, recipient mice were lethally irradiated with two doses of 5.5 Gy and intravenously injected with 5 × 10^6^ cells. Mice were administered 1 mg ml^−1^ of neomycin in their drinking water for five weeks starting one week before transplantation. Recipient mice were allowed to reconstitute immune cell populations for two months and reconstitution was routinely assessed by flow cytometry before any further intervention.

#### Treatment with maraviroc

Maraviroc (MedChem Express) was dissolved in 10% dimethylsulfoxide (Thermo Scientific, 20688), 40% polyethylene glycol 300 (Selleckchem, S6704), 5% Tween 80 (Selleckchem, S6702) and 45% saline, and the resultant solution was sonicated. Young (3 months old) and old (23 months old) C57BL/6J HccRsd mice were assigned to one of three groups: young mice were treated with a daily intraperitoneal injection of vehicle for 1 month; and old mice were treated with a daily intraperitoneal injection of either vehicle or 35 mg kg^−1^ maraviroc for 1 month.

#### Rotarod test

Motor coordination was assessed by performing the rotarod test in an accelerating rotarod apparatus (Ugo Basile). Mice were trained for 1 day at a constant speed: twice at 4 rpm for 1 min and twice with acceleration from 4 to 8 rpm for 1 min. On the second day, the rotarod was set to progressively accelerate from 4 to 40 rpm for 5 min. Mice were tested three times. During the accelerating trials, mice were video-recorded and the latency to fall from the rod was measured.

#### Forelimbs strength analysis

Limb grip strength was measured as tension force using a digital force transducer (Grip Strength Meter, Bioseb). Ten measurements were performed for each animal, with a 10-s rest period between measurements, and the average of all measurements for each mouse was compared.

#### Metabolic cages

Metabolic parameters were measured using PhenoMaster TSE-Systems. Mice were singly housed and acclimatized for 1 day before data monitoring. All parameters were measured continuously and simultaneously for 1 day.

### Tissue processing for flow cytometry

Mice were euthanized with CO_2_ followed by transcardiac perfusion with ice-cold PBS. The indicated tissues were extracted and processed as specified.

#### Spleen

Spleens were mashed and filtered through a 70-μm cell strainer. Red blood cells were removed in erythrocyte lysis buffer (ammonium chloride 0.15 M, sodium bicarbonate 0.01 M, EDTA 0.1 mM) for 5 min.

#### Blood

Blood was extracted either from the facial vein or the heart in living or euthanized mice, respectively. Red blood cells were removed in erythrocyte lysis buffer for 7 min. Cells were washed and stained.

#### Peyer’s patches or lymph nodes

Peyer’s patches and lymph nodes were harvested from the intestine and mashed into a 70-μm cell strainer. Cell suspension was centrifuged at 400*g* for 5 min at 4 °C. Cell pellets were resuspended in 2% FBS RPMI.

#### White adipose tissue

Gonadal white adipose tissue was digested into prewarmed 2 mg ml^−1^ bovine serum albumin (BSA) 2% FBS RPMI supplemented with 2 mg ml^−1^ collagenase type II (Sigma, cat. no. C6885) and placed under shaking at 180 rpm for 40 min at 37 °C. Digested tissues were vertically rested to separate fat from the aqueous phases, which were obtained using an 18G syringe needle. Cell suspensions were filtered through a 70-μm cell strainer and washed with 2% FBS RPMI. Erythrocytes were removed in lysis buffer for 5 min at 4 °C and washed with 1 mM EDTA PBS.

#### Liver

Livers were harvested and cut into prewarmed 25 mM Hepes 10% FBS RPMI supplemented with 0.4 mg ml^−1^ collagenase type VIII (Sigma, cat. no. C2139) under shaking at 180 rpm for 45 min at 37 °C. Digested tissue was filtered through a 70-μm cell strainer and centrifuged at 350*g* for 5 min at 4 °C. Red blood cells were removed in erythrocyte lysis buffer for 5 min. For leukocyte enrichment, supernatants were centrifuged in a 40%–70% Percoll gradient (Sigma, cat. no. GE17-0891-01) at 1,250*g* for 30 min at RT with acceleration on 6 and without brake. Isolated cells were washed with PBS and resuspended in 2% FBS RPMI.

#### Bone marrow

Femurs and tibias were collected. Cells from the BM were obtained by centrifuging the bones at 6,000*g* for 1 min. Red blood cells were removed in erythrocyte lysis buffer for 3 min.

#### Colon

Colons were processed as previously described^61^.

### Flow cytometry

To distinguish between live and dead cells, samples were first stained with the Zombie NIR Fixable Viability Kit (BioLegend, cat. no. 423106), the Zombie Yellow Fixable Viability Kit (BioLegend, cat. no. 423104) or the Ghost Dye Violet 540 (Tonbo Biosciences, 13-0879) for 20 min at 4 °C. Cells were washed with FACS staining buffer (PBS supplemented with 2% fetal bovine serum and 1 mM EDTA) and incubated with Fc receptor blocker purified rat anti-mouse anti-CD16/CD32 (BD Biosciences, 553142) for 20 min at 4 °C. Cells were then incubated with primary antibodies for 20 min at 4 °C and were washed twice with a FACS staining buffer. In experiments analyzing hematopoiesis, the blocking step was not performed as CD16/32 is a defining surface marker for GMPs. In those experiments, after the viability staining, we first incubated the cells with anti-CD16/32-BV510 (clone 93, BioLegend) followed by the rest of the surface marker staining. Antibodies used for surface antigen staining are detailed in Supplementary Table 1. For intracellular staining, cells were fixed and permeabilized using the FoxP3/Transcription Factor Staining Kit (eBioscience) for 20 min at room temperature in darkness. Cells were then stained with antibodies detailed in Supplementary Table 1.

Flow cytometry experiments were performed with either a four-laser (violet, blue, yellow-green, red) or five-laser (ultraviolet, violet, blue, yellow–green, red) Aurora flow cytometer (Cytek Biosciences). Data were analyzed with the FlowJo v10.5.3 software (BD Biosciences). Gating strategies were set on the basis of fluorescence minus one controls and unstained samples. All the samples in the experiment excluded dead cells, clumps and debris.

#### Analysis of mitochondrial membrane potential

Analysis of mitochondrial mass and mitochondrial membrane potential was performed by flow cytometry in cells labeled for 20 min in a 37 °C and 5% CO_2_ incubator before extracellular staining, with 50 nM MtG FM (Invitrogen, cat. no. M7514) and 25 nM TMRM (Invitrogen, cat. no. T668) in Hanks’ Balanced Salt Solution supplemented with calcium and magnesium with 150 mM KCl. To avoid extrusion of the probes through membrane efflux pumps, 50 mM Verapamil hydrochloride (Merck, cat. no. V4629) was added during the incubation of mitochondrial probes.

### Dimensional reduction and clustering analysis of flow cytometry data

Dimensional reduction and clustering analysis of flow cytometry data was performed using OMIQ (Dotmatics). First, nonlymphocyte cells, doublets and dead cells were excluded based on viability staining and forward scatter and/or side scatter parameters. Then, 35,000 CD4^+^ cells from each sample were subsampled for further analysis. For dimensional reduction, the UMAP algorithm was applied with the following parameters: Neighbors = 15, Minimum Distance = 0.4, Components = 2, Learning Rate = 1, Epochs = 200. For unbiased clustering, the Cluster-X algorithm was applied with Alpha = 0.001. The mean fluorescence intensity of each marker was projected on the UMAP plots and used to infer the identity of the clusters. Similar clusters were combined.

### Hematology

The ADVIA 2120i Hematology System (Siemens Healthineers) was used to quantitatively measure blood variables. For ADVIA measurements, 50 μl of whole blood was resuspended in 150 μl of RPMI–EDTA–K2 medium. The instrument was calibrated on the day of testing as per the manufacturer’s instructions.

### Immunohistochemistry and immunofluorescence

Organs obtained from intracardiac perfused animals were fixed in 10% neutral buffered formalin for 48 h and dehydrated in 70% ethanol until processing. Dehydrated organs were embedded in paraffin sections and processed with a microtome to generate 5-μm sections. For immunostaining, the deparaffinized sections were rehydrated and boiled to retrieve antigens (10 mM citrate buffer, 0.05% Triton X-100, pH 6). Sections were then blocked for 45 min in 10% goat serum, 5% horse serum, 0.05% Triton X-100 and 2% BSA in PBS. Endogenous peroxidase and biotin were blocked with 1% hydrogen peroxide–methanol for 10 min and a biotin blocking kit (Vector Laboratories), respectively. Sections were incubated with a goat polyclonal anti-MPO antibody (AF3667, RD Systems) and color was developed with 3,3’-diaminobenzidine (Vector Laboratories). Sections were counterstained with hematoxylin and mounted in distyrene, plasticizer and xylene (Fluka). Slides were scanned with a NanoZoomer-RS scanner (Hamamatsu). Briefly, for each animal, the number of MPO-positive cells was quantified in digitized images in which an area covering at least one-half of a section of the whole organ was previously defined. The density of cells per area was then estimated by using Zen 2.3 software (Zeiss).

### Analysis of mouse serum samples

All the plasma analysis was done by the Mouse Metabolism & Phenotyping core in Baylor College of Medicine at Houston. Glucose, albumin, ALT and AST were measured in a VS2 Vetscn2 biochemical analyzer according to manufacture instructions.

### Luminex detection of proinflammatory cytokines

Serum cytokines were detected with magnetic bead technology (Invitrogen, Cytokine & Chemokine 26-Plex Mouse ProcartaPlex PanelLuminex) following the manufacturer’s instructions.

### RNA extraction and quantitative polymerase chain reaction with reverse transcription analysis

#### RNA extraction and reverse transcription

Cultured T cells were homogenized in TRIzol reagent (Thermo Fisher Scientific). An aqueous (RNA-containing) phase was generated using 1:5 bromo-chloro–propane, mixed 1:2 with 70% isopropanol and centrifuged at 12,000*g* to precipitate RNA. Samples were treated with DNaseI (Qiagen). RNA concentration and integrity were determined by a NanoDrop One spectrophotometer (Thermo Scientific). Total RNA with an absorbance (*A*_260_/*A*_280_) ratio ranging from 1.8 to 2.2 was converted to complementary DNA using the Maxima First Strand cDNA Synthesis Kit for quantitative PCR (qPCR) with reverse transcription using dsDNase (Thermo Fisher Scientific).

#### Quantitative PCR

qPCR primers (Invitrogen-ThermoFisher) were designed using Primer-BLAST (NCBI; sequences are detailed below). A total of 10 ng of cDNA was used for qPCR in a total volume of 10 µl with GoTaq qPCR Master Mix (Promega) and specific primers, on a Bio-Rad CFX Opus 384 (Bio-Rad Laboratories). The amplification conditions were determined by the primers to present amplification efficiency close to 100% and a single peak in melt-curve analyses. Each real-time PCR reaction was performed in triplicate. *Gapdh* and *Actb* mRNAs, encoding glyceraldehyde 3-phospate dehydrogenase and β-actin housekeeping proteins, respectively, were included to monitor differences in RNA abundance. The log(fold change) in mRNA expression was calculated from ΔΔCt values relative to control samples (T cells with acute stimulation and maintained with IL-2). Oligonucleotide sequences are detailed in Supplementary Table 2.

### In vitro culturing of CD4^+^ cells

CD4^+^ T cells were purified by magnetic negative selection (Stemcell, cat. no. 19852) from the spleen and lymph nodes of 2–5-month-old mice. A density of 1 × 10^6^ CD4^+^ cells per ml were plated in complete RPMI (10% FBS, 2 mM glutamine, 50 nM β-mercaptoethanol, 25 nM HEPES and 0.5% penicillin–streptomycin) and were activated for 72 h in 20-µl anti-CD3 anti-CD28 dynabeads (ThermoFisher, cat. no. 11452D) and 20 ng ml^−1^ IL-2 (Preprotech, cat. no. 212-12) per million CD4^+^ cells. Dynabeads were removed and CD4^+^ cells were replated with the same amount of dynabeads and IL-2 (CS) or IL-2 without dynabeads (AS). Dynabeads were magnetically removed and media with each condition was refreshed every other day until the end of the experiment, with nonresting periods in the CS condition. In indicated experiments, rotenone (0.4 µM), antimycin A (0.4 µM) or cGAMP (3 µM or 10 µM) were added at day 3 and cells were analyzed at day 7.

For AgCS conditions, CD4^+^ T cells were isolated from the spleen and lymph nodes of OT-II mice by magnetic negative selection (BioLegend, cat. no. 480006). Isolated CD4^+^ T cells were co-cultured with splenocytes from *Cd3e*^*−*/*−*^ mice, which had previously been irradiated at 23 Gy and incubated for 2 h with 10 μM OVA_323–339_ peptide (Medchem Express, cat. no. HY-P0286). Co-cultures were established at a 1:1 ratio in complete RPMI medium supplemented with 10% FBS and 20 ng ml^−1^ IL-2 (Preprotech, cat. no. 212-12) and maintained for 72 h at 37 °C in a 5% CO_2_ incubator. After the initial 72 h activation, cells were washed and maintained in either complete RPMI supplemented with 10% FBS and 20 ng ml^−1^ IL-2 (antigenic acute stimulation) or restimulated with fresh *Cd3e*^*−*/*−*^ splenocytes pulsed with OVA_323–339_ peptide (AgCS). Refreshing or restimulation was performed every 2–3 days until day 7 with nonresting periods between stimulations in the AgCS conditions.

### In vitro co-culturing of CD4^+^ cells with LSK cells

Young (3–6 months old) and old (22–25 months old) C57BL/6J mice were euthanized using CO_2_, and spleens were collected. CD4^+^ T cell isolation was performed using the EasySep Mouse CD4^+^ T Cell Isolation Kit (cat. no. 19852), following the manufacturer’s instructions. The purity of the isolated cells was above 90%. Isolated CD4^+^ T cells were activated using α-CD3 and α-CD28 antibodies in complete RPMI medium supplemented with IL-2 and seeded at 5 million per ml. After 48 h of activation, the medium was replaced with fresh RPMI containing IL-2 and activated T cells seeded at 1 million per ml. Cells were cultured for an additional 24 h before starting the co-culture.

C57BL/6J Ly5.1 (CD45.1) mice were euthanized, and hips, femurs and tibias were collected. Muscles were removed, and bones were flushed by centrifugation at 10,000 *g* for 10 s. The flushed BM was filtered through a 70-µm cell strainer, and hematopoietic progenitor enrichment was immediately performed using the EasySep Mouse Hematopoietic Progenitor Cell Isolation Kit (cat. no. 19856), following the manufacturer’s instructions. Following isolation, cells were labeled with the BD Mouse Biotin Lineage Panel (cat. no. 559971). Subsequently, cells were stained with PE Streptavidin, APC–Ly-6A/E (Sca-1, clone D7) and PE-Cy7–CD117 (c-Kit, clone 2B8). Finally, cells were labeled with 4′,6-diamidino-2-phenylindole (DAPI), and live Lin⁻Sca-1^+^c-Kit^+^ (LSK) cells were sorted in a BD FACSAria Cell Sorter. LSK cell purity was assessed after cell sorting and the purity was above 98 % in all trails.

LSK cells were then seeded at 666 cells per well in a 96-well U-bottom plate, in triplicate for each biological replicate of LSK and T cells. CD4^+^ T cells were added at a 1:10 ratio (6,666 cells per well) in Stemline II medium (Sigma), supplemented with 100 ng ml^−1^ murine SCF (R&D), 2 ng ml^−1^ of murine Flt3 (R&D), 20 ng ml^−1^ murine IL-3 (R&D) and murine IL-2 (Thermo Scientific, 212-12). This ratio was adjusted based on ratios typically observed in vivo in aged mice. After 96 h of co-culture, cells were isolated and stained with FITC–CD45.1 (clone A20), Pacific Blue–CD45.2 (clone 104), Gr-1 (clone RB6-8C5), PE–CD3 (clone 17A2), PE-Cy7–B220 (clone RA3-6B2), and propidium iodide for viability assessment.

For experiments involving CCL5 blockade media was supplemented with 5 μg ml^−1^ of an anti-CCL5 blocking antibody (BioLegend, cat. no. 947003) or same concentration of a purified rat IgG2a, λ isotype control antibody (BioLegend, cat. no. 402301).

### Cytotoxicity assay

To evaluate the cytotoxicity of in vitro differentiated CD4^+^ CTLs, B cells presenting OVA_323–339_ peptide were co-cultured with CS in in vitro stimulated CD4^+^ cells from OT-II mice. Cytotoxicity was assessed based on B cell viability. Briefly, OVA–peptide–IgM-conjugated microbeads were prepared the day after the cytotoxicity assay. First, 5 μl of streptavidin-coated microspheres 0.1 μm in diameter (Bangs Laboratories, cat. no. CP01000) were washed with 200 μl of FACS buffer (PBS supplemented with EDTA 1 mM and 2% FBS), centrifuged at maximum speed for 5 min, resuspended in 20 μl of FACS buffer and sonicated for 5 min. Then 20 μl of these sonicated beads were mixed with 2.5 μg of biotin rat anti-mouse IgM (BD Bioscience, cat. no. 553436), 2.5 μg of biotin–OVA_323–339_ peptide (SB-Peptide, cat. no. SB075) and 250 μl of FACS buffer and incubated for 1 h at 37 °C in a water bath. The OVA_323–339_ peptide–IgM-conjugated microbeads were then washed twice with FACS, centrifuged at maximum speed between washes, and resuspended in 1.25 ml of complete RPMI supplemented with 10% FBS and stored in the refrigerator.

On the day of the cytotoxicity assay, B cells were isolated from spleens of young (3–5-month-old) mice using a negative selection B Cell Isolation Kit (Miltenyi Biotec, cat. no. 130-090-862) according to the manufacturer’s instructions. For the OVA_323–339_ peptide–IgM-conjugated microbeads, internalization by B cells, 130 μl of the prepared beads per 1 million B cells were added to a suspension of 1 × 10^7^ B cells per ml. The mixture was spun down for 8 s at maximum speed and incubated for 30 min at 37 °C in a water bath. Half of the isolated B cells were kept untreated as controls.

Splenic CD4^+^ T cells from OT-II mice, either AS or CS for 6 days, were co-cultured at a 1:1 ratio with B cells, with or without OVA_323–339_, for 6 h at 37 °C with 5% of CO_2_, at a final density of 1 × 10^6^ cells per ml in complete RPMI supplemented with 10% FBS. B cells with OVA_323–339_ were cultured at the same density without T cells as viability controls. After co-incubation, cells were prepared for viability readout by spectral flow cytometry staining of Annexin V.

### Seahorse analysis of oxygen consumption

CD4^+^ T cells were purified from the spleen and lymph nodes of 2-month-old C57BL/6J mice and were either acutely or chronically stimulated as described in the section ‘In vitro culturing of CD4^+^ cells’. The oxygen consumption rate of wells containing 200,000 cells was measured in a Seahorse XFe96 extracellular flux analyzer (Agilent) using the Seahorse XF T Cell Metabolic Profiling kit (Agilent) according to the manufacturer’s instructions.

### Immunocytofluorescence

CD4^+^ T cells purified from young (2-month-old) or old (24-month-old) mice by negative selection were allowed to attach to poly-L-lysine coated cover slips for 4 h and fixed in 3% paraformaldehyde for 10 min. Cells were incubated with a blocking buffer containing 5% BSA in PBS with 0.1% Tween 20 for 1 h followed by permeabilization with 0.3% Triton X-100 in PBS for 10 min at room temperature. Cells were incubated with an anti-DNA mouse monoclonal antibody (Progen, clone AC-30-10, cat. no. 690014) and an anti-TOMM20 rabbit polyclonal antibody (Abcam, cat. no. 19403) overnight at 4 °C. Nuclei were counterstained with DAPI for 10 min. Stimulated emission depletion images were obtained in a confocal microscope Leica Stellaris 8 (Leica Microsystems).

### scRNA-seq analysis

Published data used in this study were retrieved from Single Cell Portal SCP490^14^; Gene Expression Omnibus, accession number GSE174440^51^; Genome Sequence Archive, accession number CRA0046603^16^; and single-cell data obtained upon request^62^.

The scRNA-seq data from Extended Data Fig. 7 were reanalyzed with the Seurat package^63^ v4.2.0 in R v4.1.3. Variable genes were identified with the FindVariablesFeatures function across the range of expression values and used to perform a principal component analyses with the RunPCA function. Clustering was performed with the FindClusters function with the Leiden algorithm and the first 20 principal components. Clusters identification was done with the FindMarkers function in each subset with a minimum log fold change of 0.25 and a *P* value <0.05.

The scRNA-seq data from the BM of young and old mice at steady-state in Extended Data Fig. 2 were reanalyzed with the Seurat package v5.1.0 in R v4.4.1. Cells expressing <200 or >6,000 unique genes and a mitochondrial gene expression percentage >0.05 were excluded from the study. The top 2,000 variable genes were used to harmonize and integrate the samples following the Seurat analysis workflow. Neutrophils were identified by considering the expression of *Ly6G*, *Csf3r* and *Ngp* genes, and a new complete Seurat analysis was performed on this selected population. Five populations were detected at resolution 0.3 using the FindClusters function. The differential gene expression analysis was performed using the FindAllMarkers function. Further comparison was then conducted between clusters 0 and 1. The UMAPs were generated using the DimPlot, FeaturePlot_scCustom and Plot_Density_Custom functions from the packages Seurat, v5.1.0, ggplot2, v3.4.4, scCustomize^64^, v2.1.2 and Nebulosa5^65^, v1.14.0.

The scRNA-seq data in BM samples from young and old mice in Extended Data Fig. 6 were reanalyzed with Seurat package v5.3.0 in R v4.5.1. Cells with more than 750 unique molecule identifiers, 300 unique genes, a logarithmic ratio superior to 0.85 between the number of unique genes and unique molecule identifiers, and mitochondrial gene expression <0.20 were included in the study. The data were normalized and scaled and variable genes were identified with the FindVariablesFeatures function across the range of expression values and used to perform a principal component analyses with the RunPCA function. Clustering was performed with the FindClusters function with the Louvain algorithm and the first 20 principal components. The clusters were identified using the comparison with other scRNA-seq data with the use of the Clustifyr v1.20.0 and clustifyrdatahub v1.18.0 packages^66^. A subset of the cells identified as CD4^+^ T cells were made and reanalyzed. Clusters identification and differential gene expression was done with the FindMarkers function in each subset with a minimum log(fold change) of 0.25 and an adjusted *P* value <0.05 and the Clustifyr package.

Gene Set Enrichment Analysis^67^ and Over Representation Analysis were performed using the clusterProfiler^68^ package, v4.8.3, in the Gene Ontology, Kyoto Encyclopedia of Genes and Genomes, WikiPathways, Reactome and MSigDB databases. Data visualization was performed using the GSEA.barplot function in the PPInfer package v1.26.0 and the pheatmap function in the pheatmap package v1.0.12.

### Statistics and reproducibility

Statistical analysis was performed with GraphPad Prism (version 9). Data are expressed as mean ± s.e.m. Outliers were excluded by the ROUT method (5%). For all comparisons, normality of distribution was assesed using the Shapiro–Wilk normality test. Statistically significant *P* values are indicated above the corresponding bars in the figures. Statistical tests for each comparison are detailed in the Source Data.

Unless otherwise specified, *n* represents the number of individual biological replicates and is represented in the graphs as one dot per sample. Flow cytometry plots are representative of at least three replicates. No statistical method was used to predetermine sample size, but a minimum of three samples were used per experimental group and condition. Data collection and analysis were not performed blind to the conditions of the experiments. No method of randomization was used to assign experimental groups.

### Reporting summary

Further information on research design is available in the Nature Portfolio Reporting Summary linked to this article.

## Supplementary information

Reporting Summary

Peer Review File

Supplementary Table 1 Antibodies for intracellular and extracellular staining by flow cytometry.

Supplementary Table 2 Oligonucleotide sequences for RT–PCR analysis.

## Source data

Source Data Fig. 1 Statistical source data.

Source Data Fig. 2 Statistical source data.

Source Data Fig. 3 Statistical source data.

Source Data Fig. 4 Statistical source data.

Source Data Fig. 5 Statistical source data.

Source Data Extended Data Fig. 1 Statistical source data.

Source Data Extended Data Fig. 3 Statistical source data.

Source Data Extended Data Fig. 4 Statistical source data.

Source Data Extended Data Fig. 5 Statistical source data.

Source Data Extended Data Fig. 6 Statistical source data.

Source Data Extended Data Fig. 7 Statistical source data.

Source Data Extended Data Fig. 8 Statistical source data.

## Data Availability

All data supporting the findings of this study are available in the source data, and from the corresponding author upon request. The scRNA-seq datasets reanalyzed are publicly available and accordingly cited in the corresponding methods section. Source data are provided with this paper.
