# Supplementary material for: Cytotoxic CD4+ T cells induce age-associated myelopoiesis through CCL5–CCR5 signaling
Source: Nat Aging. 2026 Aug 26;6(10):2110–25. doi: 10.1038/s43587-026-01209-9 (PMC13661277; doi:10.1038/s43587-026-01209-9)
Supplement: Supplementary file 1 [file 43587_2026_1209_MOESM1_ESM.pdf]

nature portfolio

Corresponding author(s): María Mittelbrunn
Enrique Gabandé-Rodríguez

Last updated by author(s): May 28, 2026

Reporting Summary

Nature Portfolio wishes to improve the reproducibility of the work that we publish. This form provides structure for consistency and transparency in reporting. For further information on Nature Portfolio policies, see our Editorial Policies and the Editorial Policy Checklist.

Statistics

For all statistical analyses, confirm that the following items are present in the figure legend, table legend, main text, or Methods section.

n/a	Confirmed	
☐	☒	The exact sample size (*n*) for each experimental group/condition, given as a discrete number and unit of measurement
☐	☒	A statement on whether measurements were taken from distinct samples or whether the same sample was measured repeatedly
☐	☒	The statistical test(s) used AND whether they are one- or two-sided<br>*Only common tests should be described solely by name; describe more complex techniques in the Methods section.*
☒	☐	A description of all covariates tested
☐	☒	A description of any assumptions or corrections, such as tests of normality and adjustment for multiple comparisons
☐	☒	A full description of the statistical parameters including central tendency (e.g. means) or other basic estimates (e.g. regression coefficient) AND variation (e.g. standard deviation) or associated estimates of uncertainty (e.g. confidence intervals)
☐	☒	For null hypothesis testing, the test statistic (e.g. *F*, *t*, *r*) with confidence intervals, effect sizes, degrees of freedom and *P* value noted<br>*Give P values as exact values whenever suitable.*
☒	☐	For Bayesian analysis, information on the choice of priors and Markov chain Monte Carlo settings
☒	☐	For hierarchical and complex designs, identification of the appropriate level for tests and full reporting of outcomes
☐	☒	Estimates of effect sizes (e.g. Cohen's *d*, Pearson's *r*), indicating how they were calculated

Our web collection on statistics for biologists contains articles on many of the points above.

Software and code

Policy information about availability of computer code

Data collection	The scRNAseq data used in Extended Data Figure 6 and 7 were collected from "Aging promotes reorganization of the CD4 T cell landscape toward extreme regulatory and effector phenotypes" Elyahu et al. 2019, Science Advances (doi: 10.1126/sciadv.aaw8330) and from "Heterochronic parabiosis induces stem cell revitalization and systemic rejuvenation across aged tissues.Ma et al. 2022, Cell Stem Cell (https://doi.org/10.1016/j.stem.2022.04.017).<br>The sRNAseq data sed in Extended Data Figure 2 were collected from "Age-induced alterations of granulopoiesis generate atypical neutrophils that aggravate stroke pathology" Gullota et al. 2019, NatureImmunology (DOI: 10.1038/s41590-023-01505-1) and from "Age-Related Alterations in Peripheral Immune Landscape with Magnified Impact on Post-Stroke Brain." Lu et al. 2023, Research (Wash D C) (doi.org/10.34133/research.0287) upon request to authors.
Data analysis	Published data used in this study were retrieved from Single Cell Portal SCP490 24; Gene Expression Omnibus (GEO); accession number GSE174440 19, Genome Squence Archive (GSA); accession number CRA0046603 26 and single cell data obtained upon requestion from Lu et al 20.<br>The scRNAseq data from ED Fig.7 were reanalysed with the Seurat package 66 v4.2.0 in R v4.1.3. Variable genes were identified with the FindVariablesFeatures function across the range of expression values and used to perform a principal component analyses (PCA) with the RunPCA function. Clustering was performed with the FindClusters function with the Leiden algorithm and the first 20 principal components. Clusters identification was done with the FindMarkers function in each subset with a minimum log fold change of 0.25 and a P-value <0.05.<br>The scRNAseq data from the BM of young and old mice at steady-state in Extended Data Fig.2 were reanalysed with the Seurat package v5.1.0 in R v4.4.1. Cells expressing <200 or > 6,000 unique genes and a mitochondrial gene expression percentage >0.05 were excluded from the study. The top 2,000 variable genes were used to harmonize and integrate the samples following the Seurat analysis workflow. The neutrophils were identified by considering the expression of Ly6G, Csf3r and Ngp genes, and a new complete Seurat analysis was performed on this selected population. Five populations were detected at resolution 0.3 using the FindClusters function. The differential gene expression

analysis was performed using the FindAllMarkers function. Then, a further comparison was conducted between the clusters 0 and 1. The UMAPs were generated using the DimPlot, FeaturePlot_scCustom and Plot_Density_Custom functions from the packages Seurat, v5.1.0, ggplot2, v3.4.4, scCustomize 67, v2.1.2, and Nebulosa5 68, v1.14.0.
The scRNAseq data in BM samples from young and old mice in Extended Fig.6, were reanalyzed with the Seurat package v5.3.0 in R v4.5.1. Cells with more than 750 unique molecule identifiers, 300 unique genes, a logarithmic ratio superior to 0.85 between the number of unique genes and unique molecule identifiers and a mitochondrial gene expression <0.20 were included in the study. The data were normalized and scaled and variable genes were identified with the FindVariablesFeatures function across the range of expression values and used to perform a principal component analyses (PCA) with the RunPCA function. Clustering was performed with the FindClusters function with the Louvain algorithm and the first 20 principal components. The clusters were identified using the comparison with other scRNAseq data with the use of the Clustifyr v1.20.0 and clustifyrdatahub v1.18.0 packages 69. A subset of the cells identified as CD4+ T cells were made and reanalyzed. Clusters identification and differential gene expression was done with the FindMarkers function in each subset with a minimum log fold change of 0.25 and an adjusted P-value <0.05 and the Clustifyr package.
Gene Set Enrichment Analysis (GSEA) 70 and Over Representation analysis (ORA) were performed using the clusterProfiler 71 package, v4.8.3, in GO, KEGG, WikiPathways, Reactome and MSigDB databases. The data visualization was performed using the GSEA.barplot function in the PPInfer package v1.26.0 and the pheatmap function in the pheatmap package v1.0.12.

For manuscripts utilizing custom algorithms or software that are central to the research but not yet described in published literature, software must be made available to editors and reviewers. We strongly encourage code deposition in a community repository (e.g. GitHub). See the Nature Portfolio guidelines for submitting code & software for further information.

Data

Policy information about availability of data

All manuscripts must include a data availability statement. This statement should provide the following information, where applicable:

- Accession codes, unique identifiers, or web links for publicly available datasets
- A description of any restrictions on data availability
- For clinical datasets or third party data, please ensure that the statement adheres to our policy

The scRNAseq data used in Extended Data Figure 6 and 7 were collected from "Aging promotes reorganization of the CD4 T cell landscape toward extreme regulatory and effector phenotypes" Elyahu et al. 2019, Science Advances (doi: 10.1126/sciadv.aaw8330) and from "Heterochronic parabiosis induces stem cell revitalization and systemic rejuvenation across aged tissues.Ma et al. 2022, Cell Stem Cell (https://doi.org/10.1016/j.stem.2022.04.017).
The sRNAseq data sed in Extended Data Figure 2 were collected from "Age-induced alterations of granulopoiesis generate atypical neutrophils that aggravate stroke pathology" Gullota et al. 2019, NatureImmunology (DOI: 10.1038/s41590-023-01505-1) and from "Age-Related Alterations in Peripheral Immune Landscape with Magnified Impact on Post-Stroke Brain." Lu et al. 2023, Research (Wash D C) (doi.org/10.34133/research.0287) upon request to authors.

All data supporting the findings of this study are available as source data, and from the corresponding author upon request. The scRNA-seq datasets reanalyzed are publicly available and accordingly cited in the corresponding methods section.

Research involving human participants, their data, or biological material

Policy information about studies with human participants or human data. See also policy information about sex, gender (identity/presentation), and sexual orientation and race, ethnicity and racism.

Reporting on sex and gender	NA
Reporting on race, ethnicity, or other socially relevant groupings	NA
Population characteristics	NA
Recruitment	NA
Ethics oversight	NA

Note that full information on the approval of the study protocol must also be provided in the manuscript.

Field-specific reporting

Please select the one below that is the best fit for your research. If you are not sure, read the appropriate sections before making your selection.

☒ Life sciences ☐ Behavioural & social sciences ☐ Ecological, evolutionary & environmental sciences

For a reference copy of the document with all sections, see nature.com/documents/nr-reporting-summary-flat.pdf

Life sciences study design

All studies must disclose on these points even when the disclosure is negative.

Sample size	No statistical method was used to predetermine sample size, but a minimum of four samples (or 3 independent cultures) were used per

Sample size	experimental group and condition. Four samples were considered as the minimun to reduce the number of animals and have enough information for statistical tests. In addition, some experiments were performed with more mice because they were initially caged together. For all experiments the exact n is detailed in the legend to each figure.
Data exclusions	Mice with signs of unhealthy aging (dermatitis, tumours) were removed before performing the experiments. Outliers were identified by the ROUT method (5%) and removed.
Replication	Representative plots and graphs summarize results of at least two independent experiments
Randomization	Mice were grouped by their date of birth for this study.
Blinding	Data collection and analysis were not performed blind to the conditions of the experiments.

Reporting for specific materials, systems and methods

We require information from authors about some types of materials, experimental systems and methods used in many studies. Here, indicate whether each material, system or method listed is relevant to your study. If you are not sure if a list item applies to your research, read the appropriate section before selecting a response.

Materials & experimental systems

n/a	Involved in the study	
☐	☒	Antibodies
☒	☐	Eukaryotic cell lines
☒	☐	Palaeontology and archaeology
☐	☒	Animals and other organisms
☐	☒	Clinical data
☒	☐	Dual use research of concern
☒	☐	Plants

Methods

n/a	Involved in the study	
☒	☐	ChIP-seq
☐	☒	Flow cytometry
☒	☐	MRI-based neuroimaging

Antibodies

Antibodies used

CD8 BUV615 53-6.7 Thermo Fisher Scientific
CD127 (IL-7R) BUV737 SB/199 BD Horizon
Ly6G BV421 1A8 Biolegend
CD186 (CXCR6) BV421 SA051D1 Biolegend
Streptavidin BV421 405225
Biolegend
Lineage BV421 133311 Biolegend
CD38 Pacific Blue 90 Biolegend
TIM3 BV480 5D12/TIM-3 BD Biosciences
CD19 V450 1D3 Tonbo Biosciences
CD45.2 V500 104 Tonbo Biosciences
CD16/32 BV510 93 Biolegend
CD44 BV570 IM7 Biolegend
CD66a (CEACAM1a) BV650 MAB-CC1 Biolegend
CD223 (LAG3) BV650 C9B7W Biolegend
CD62L BV711 MEL-14 Biolegend
Ly6C BV785 HK1.4 Biolegend
B220 FITC RA3-6B2 Tonbo Biosciences
CD34 FITC RAM34 BD Biosciences
CD62L FITC MEL-14 Tonbo Biosciences
FoxP3 FITC Fjk-16s Invitrogen
Ly6C PerCP Cy5.5 HK1.4 Biolegend
CD44 PerCP Cy5.5 IM7 Tonbo Biosciences
CD45.2 PerCP Cy5.5 104 Biolegend
PD1 PerCP eFluor710 J43 Thermo Fisher Scientific
CD135 (FLT3) PerCP eFluor710 A2F10 Thermo Fisher Scientific
CD8 PE 53-6.7 Tonbo Biosciences
CD117 (cKit) PE ACK2 Tonbo Biosciences
NKG2D PE-Dazzle594 CX5 Biolegend
CD25 PE Cy5 PC61 Biolegend
CD195 (CCR5) Alexa Fluor 647 HEK/1/85a Biolegend
Ly6G PE-Cy7 1A8 Biolegend
CD101 PE-Cy7 Moushi101 Thermo Fisher Scientific
SLAM (CD150) PE-Cy7 TC15-12F12.2 Biolegend
CD54 (ICAM-1) PE-Cy7 YN1/1.7.4 Biolegend
Annexin V PE-CF594 RUO BD Biosciences

CX3CR1 APC SA011F11 Biolegend
CD48 APC HM48-1 Biolegend
Sca-1 APC-Cy7 D7 Biolegend
CD45 APC-Cy7 30-F11 Biolegend
CD45.1 APC-Cy7 A20 Tonbo Biosciences
CD279 (PD-1) APC-Fire750 29F.1A12 Biolegend
CD19 APC-efluor780 eBio1D3 ThermoScientific
CD4 APC Fire810 Gk1.5 Biolegend
Blimp-1 BV421 5-E7 BD Biosciences
FoxP3 FITC Fjk-16s Invitrogen
Granzyme A Alexa Fluor 488 3G8.5 Santa Cruz
T-bet Real Blue 780 04-46 BD Biosciences
CCL5 PE 2E9/CCL5 Biolegend
Granzyme K PE GM6C3/SC-56125 Santa Cruz
CCL5 PE-Cy7 2E9/CCL5 Biolegend
EOMES PE-Cy7 Dan11mag Invitrogen
Granzyme B PE-Cy7 NGZB eBiosciences
Perforin APC S16009A Biolegend
T-bet APC 4B10 Biolegend
TOX eFluor660 TRX10 Invitrogen
Granzyme B Alexa Fluor 700 GB11 BD Biosciences
BHLHE40 Alexa Fluor 700 #NB100-1800AF700 Novus Biologicals

Validation

All the antibodies used for flow cytometry were purchased to suppliers. The antibodies were validated by the supplier and previously published papers.

Animals and other research organisms

Policy information about studies involving animals; ARRIVE guidelines recommended for reporting animal research, and Sex and Gender in Research

Laboratory animals

Most of the experiments were performed with females due to the possibility to recage them. In the few experiments in which males were used this has been specified in the figure legend. Ages of the mice are indicated in each figure.

Wild animals

The study did not involve wild animals.

Reporting on sex

Findings apply to both sexes of mice.

Field-collected samples

The study did not involve field-collected samples.

Ethics oversight

All the procedures with animals were previously evaluated and approved (PROEX 287/16 and PROEX 52.1/23) by the Ethics commitee on animal experimentation of the CBMSO, the authorized commitee of the Spanish National Research Council or the Universidad Autónoma de Madrid and the regional government (Comunidad de Madrid)

Note that full information on the approval of the study protocol must also be provided in the manuscript.

Clinical data

Policy information about clinical studies

All manuscripts should comply with the ICMJE guidelines for publication of clinical research and a completed CONSORT checklist must be included with all submissions.

Clinical trial registration

Provide the trial registration number from ClinicalTrials.gov or an equivalent agency.

Study protocol

Note where the full trial protocol can be accessed OR if not available, explain why.

Data collection

Describe the settings and locales of data collection, noting the time periods of recruitment and data collection.

Outcomes

Describe how you pre-defined primary and secondary outcome measures and how you assessed these measures.

Plants

Seed stocks

Report on the source of all seed stocks or other plant material used. If applicable, state the seed stock centre and catalogue number. If plant specimens were collected from the field, describe the collection location, date and sampling procedures.

Novel plant genotypes

Describe the methods by which all novel plant genotypes were produced. This includes those generated by transgenic approaches, gene editing, chemical/radiation-based mutagenesis and hybridization. For transgenic lines, describe the transformation method, the number of independent lines analyzed and the generation upon which experiments were performed. For gene-edited lines, describe the editor used, the endogenous sequence targeted for editing, the targeting guide RNA sequence (if applicable) and how the editor was applied.

Authentication

Describe any authentication procedures for each seed stock used or novel genotype generated. Describe any experiments used to assess the effect of a mutation and, where applicable, how potential secondary effects (e.g. second site T-DNA insertions, mosiacism, off-target gene editing) were examined.

Flow Cytometry

Plots

Confirm that:

☒ The axis labels state the marker and fluorochrome used (e.g. CD4-FITC).

☒ The axis scales are clearly visible. Include numbers along axes only for bottom left plot of group (a 'group' is an analysis of identical markers).

☒ All plots are contour plots with outliers or pseudocolor plots.

☒ A numerical value for number of cells or percentage (with statistics) is provided.

Methodology

Sample preparation

Mice were euthanized with CO_2 followed by transcardiac perfusion with ice-cold PBS. The indicated tissues were extracted and processed as specified:

Spleen

Spleen was mashed and filtered through a 70-µm cell strainer. Red blood cells were removed in erythrocyte lysis buffer (ammonium chloride 0.15 M, sodium bicarbonate 0.01 M, EDTA 0.1 mM) for 5 min.

Blood

Blood was extracted either from the facial vein or the heart in living or euthanized mice, respectively. Red blood cells were removed in erythrocyte lysis buffer for 7 min. Cells were washed and stained.

Peyer’s Patches or lymph nodes

Peyer’s patches and lymph nodes were harvested from the intestine and mashed into a 70-µm cell strainer. Cell suspension was centrifuged at 400 x g for 5 min at 4ºC. Cell pellets were resuspended in 2% FBS RPMI.

White adipose tissue

Gonadal white adipose tissue was digested into pre-warmed 2 mg/ml BSA 2% FBS RPMI supplemented with 2 mg/ml collagenase type II (Sigma, C6885) and placed under shaking at 180 rpm for 40 min at 37ºC. Digested tissues were vertically rested to separate fat from the aqueous phases, which were obtained using an 18G syringe needle. Cell suspensions were filtered through a 70-µm cell strainer and washed with 2% FBS RPMI. Erythrocytes were removed in lysis buffer for 5 min at 4ºC and washed with 1 mM EDTA PBS.

Liver

Liver was harvested and cut into pre-warmed 25 mM Hepes 10% FBS RPMI supplemented with 0.4 mg/ml collagenase type VIII (Sigma, C2139) under shaking at 180 rpm for 45 min at 37ºC. Digested tissue was filtered through a 70-µm cell strainer and centrifuged at 350 x g for 5 min at 4 ºC. Red blood cells were removed in erythrocyte lysis buffer for 5 min. For leucocyte enrichment, supernatants were centrifuged in a 40%/70% Percoll gradient (Sigma, GE17-0891-01) at 1250 x g for 30 min at RT with acceleration on 6 and without brake. Isolated cells were washed with PBS and resuspended in 2% FBS RPMI.

Bone marrow

Femurs and tibias were collected. Cells from the bone marrow were obtained by centrifuging the bones at 6000 g for 1 min. Red blood cells were removed in erythrocyte lysis buffer for 3 min.

Colon

Colons were processed as previously described 65.

Instrument

All flow cytometry experiments were performed with a 4-laser (Violet, blue, yellow-green, red) or a 5-laser (Ultraviolet, violet, blue, yellow-green, red) Aurora flow cytometers (Cytek Biosciences).

Software

Data were analyzed with the FlowJo v10.5.3 software (BD Biosciences).
Dimensional reduction and clustering analysis of flow cytometry data was done using OMIQ (Dotmatics).

Cell population abundance

There were no sortings in this study.

Gating strategy

The gating strategy was:
1. Lymphocytes in FSC-A / SSC-A plot.
2. Single cells as a diagonal line in FSC-H / FSC-A plot.
3. Alive cells in a FSC-A / Viability marker.
4. CD4+ cells in CD4 / CD8 plot.

Boundaries between positive and negative populations were stablished above 10^3 and when the two populations were clearly defined.

For other gating strategies, details have been specified in the main text or included in Extended Data Fig. 4 and 7

☒ Tick this box to confirm that a figure exemplifying the gating strategy is provided in the Supplementary Information.
