# Supplementary material for: Cytotoxic CD4+ T cells induce age-associated myelopoiesis through CCL5–CCR5 signaling
Source: Nat Aging. 2026 Aug 26;6(10):2110–25. doi: 10.1038/s43587-026-01209-9 (PMC13661277; doi:10.1038/s43587-026-01209-9)
Supplement: Supplementary file 2 [file 43587_2026_1209_MOESM2_ESM.pdf]

nature portfolio

Peer Review File

Cytotoxic CD4+ T cells induce age-associated myelopoiesis through CCL5/CCR5 signalling

Corresponding Author: Dr Maria Mittelbrunn

This manuscript has been previously reviewed at another journal. This document only contains information relating to versions considered at Nature Aging.

Version 0:

Reviewer comments:

Reviewer #1

(Remarks to the Author)
In their manuscript, Gabandé-Rodríguez E et al describe a previously unrecognised mechanism driving enhanced myelopoesis during ageing, through cytotoxic T cells recirculating to the bone marrow (BM) and overproducing CCL5 - this mechanism skews
haematopoiesis towards myelopoiesis and granulopoiesis, and it can be blocked through the CCL5 receptor CCR5, which can rejuvenate some features in aged mice. These are very exciting findings that are overall well supported. The authors build on previous observations by other groups (doi 10.1126/sciadv.aaw8330) suggesting cytotoxic T cells overexpressing CCL5 accumulate during ageing, to investigate their possible role in declined function of the haematopoietic system in the elderly.

Several firsts in this manuscript comprise 1) the identification of differentiation pathways of $CD4^+$ cytotoxic T lymphocytes (CTLs) and the regulatory mechanisms governing CCL5 expression in $CD4^+$ CTLs; 2) the demonstration of the functional role of $CD4^+$ CTLs in promoting myelopoiesis and the identification of key molecular drivers; 3) the suggestion that the CCL5/CCR5 axis represents a modifiable pathway with potential to reverse age-related
immune alterations. These are highly interesting findings with potential therapeutic implications, given the FDA approval or CCR5 inhibitor. The data, using a variety of unbiased independent approaches, supports the main conclusions.

As a minor point, author suggest the central role of neutrophils in this context. However, neutrophils are heterogenous, and it would be useful to know which neutrophils (young/aged, pro-inflammatory, senescent, effector, etc) are regulated by this axis and how.

To be conservative on the authors' interpretation, some limitations should be acknowledged, and particularly causality demonstrated through CCL5 deletion in T cells, which is beyond the scope of reasonable revisions, but would provide definitive proof of the core message of this study. This does not detract the overall relevance and novelty of this study, but would acknowledge certain limitations.

Reviewer #2

(Remarks to the Author)
The revised manuscript "Cytotoxic CD4+ T cells induce age-associated myelopoiesis through CCL5/CCR5 signalling" lays out a more clear story of how increased Cd4+ T cells with age acts on HSCs to induce myelopoiesis through the Ccl5-Ccr5 axis, with mechanistic details including STING and mitochondrial stress. The addition of acknowledgements on the known involvement of T-cell-originating Ccl5 in myeloid bias in disease conditions puts the novelty of the findings in regards to aging into better perspective.

Notwithstanding this, there are issues that remain. It is confusing to see in the answer to reviewers in Fig. r2-4 that Ccl5 is produced by both T cells and NK cells (which is known in the literature) while the heading of page 4 is "Exclusive release of CCL5 by CD4+ T cells". This means that Ccl5 from T cells, while sufficient to induce myelopoiesis, may not be the only way to myelopoiesis through the Ccl5-Ccr5 axis. It could also come from NK cells, however, NK cells are not mentioned in the

manuscript. The pharmaceutical intervention that acts on Ccr5 wouldn't differentiate between the sources of Ccl5, and doesn't directly assess T-cell contribution to myelopoiesis. Moreover, this opens the larger question of other known producers of Ccl5 in the bone marrow. In fact, a 2012 paper from the Goodell lab (Ergen et al Blood 2012 PMCID: PMC3311273) was the first to report that Ccl5 acts on HSCs to induce myeloid skewing in aging. While identifying T-cells as major producers of Ccl5, a greatest source of Ccl5 was identified as the BM niche within the context of aging. The Ergen et al paper is not cited in this current manuscript, and it should be, as well as putting these data into larger context of what is already known. While the authors do not claim that T-cells are the only producers of Ccl5, there should be an acknowledgement that it is known that a) other cells produce Ccl5 and this production is or may be increased with age and b) increase of Ccl5 in the BM has already been shown to induce myeloid skewing in aging.

Lastly, the human data (Fig. 2e and Extended Data Fig. 4e) is not convincing. In Fig. 2e, the variance explained by age alone is very small, and there appear to be influential outliers that pull the association in a positive direction. In Extended Data Fig. 4e, the R2 value is mistakenly written as negative. The relationship between age and CD4+ CD57+ cells in humans is weak, a point that is not acknowledged in the manuscript, and I would strongly suggest removing these data from the manuscript completely.

Reviewer #3

(Remarks to the Author)
The authors have addressed my previous concerns. I have no additional comments.

Open Access This Peer Review File is licensed under a Creative Commons Attribution 4.0 International License, which permits use, sharing, adaptation, distribution and reproduction in any medium or format, as long as you give appropriate credit to the original author(s) and the source, provide a link to the Creative Commons license, and indicate if changes were made.
In cases where reviewers are anonymous, credit should be given to 'Anonymous Referee' and the source.
The images or other third party material in this Peer Review File are included in the article's Creative Commons license, unless indicated otherwise in a credit line to the material. If material is not included in the article's Creative Commons license and your intended use is not permitted by statutory regulation or exceeds the permitted use, you will need to obtain permission directly from the copyright holder.
To view a copy of this license, visit https://creativecommons.org/licenses/by/4.0/

Referees' comments:

Referee #1 (Remarks to the Author):

The revised manuscript by Gabandé-Rodríguez E *et al* provides additional evidence fully supporting a previously unrecognised mechanism driving enhanced myelopoesis during ageing, through cytotoxic T cells recirculating to the bone marrow (BM) and overproducing CCL5 - this mechanism skews haematopoiesis towards myelopoiesis and granulopoiesis, and it can be blocked through the CCL5 receptor CCR5, which can rejuvenate some features in aged mice. These are very exciting findings that are well supported by the new data. Several firsts in this manuscript comprise the discovery that cytotoxic T cells promote myelopoiesis during ageing, the identification of CCL5 as a targetable mediator of this regulation and the finding that the differentiation of haematopoietic stem cells (HSCs) expressing the CCL5 receptor CCR5 is regulated by CCL5-producing cytotoxic T cells. These are highly interesting findings with potential therapeutic implications, given the FDA approval of CCR5 inhibitor. The data, using a variety of unbiased independent approaches, clearly supports the main conclusions.

The new data suggesting cell-extrinsic increase of CXCR4hi CD62Llo neutrophils during ageing is very interesting. These new data and results highlighted in grey and added to the revised manuscript support the overall message and should be kept within the manuscript, it my opinion. However, it should be clarified whether this phenotype is corrected by CCR5i, or not. A previous study has shown increased levels of CXCR4hi CD62Llo neutrophils in age-related myeloproliferative disorders, dependently on GM-CSF and JAK-STAT5 signalling (doi: 10.1182/blood.2024027455). Figure 5g shows increased levels of GM-CSF in aged mice, and this is not corrected by CCR5i maraviroc. Were levels of CXCR4hi CD62Llo neutrophils corrected by maraviroc or not? If not, what could regulate their levels during ageing? This could be discussed in the context of the aforementioned study.

We thank the reviewer for the time spent in the long process of revision of this manuscript and for appreciating the novelty and relevance of our findings. Following criticisms by the other reviewers and in order to simplify the manuscript we have chosen to submit a revised version of the manuscript not including this data. However, to answer reviewer comments on this, we followed two alternative approaches.

First, we performed BM transplantation assays from CCR5$^{+/+}$ and CCR5$^{-/-}$ into young or aged recipients. Alternatively, we treated young and old mice with Maraviroc and analysed the levels of CXCR4^{hi}CD62L^{lo} neutrophils in the blood. As shown in ***Fig.R1-1a,b****, both experiments confirmed that CCR5 inhibition did not decreased the percentage of CXCR4^{hi}CD62Ll^{o} neutrophils in the blood.*

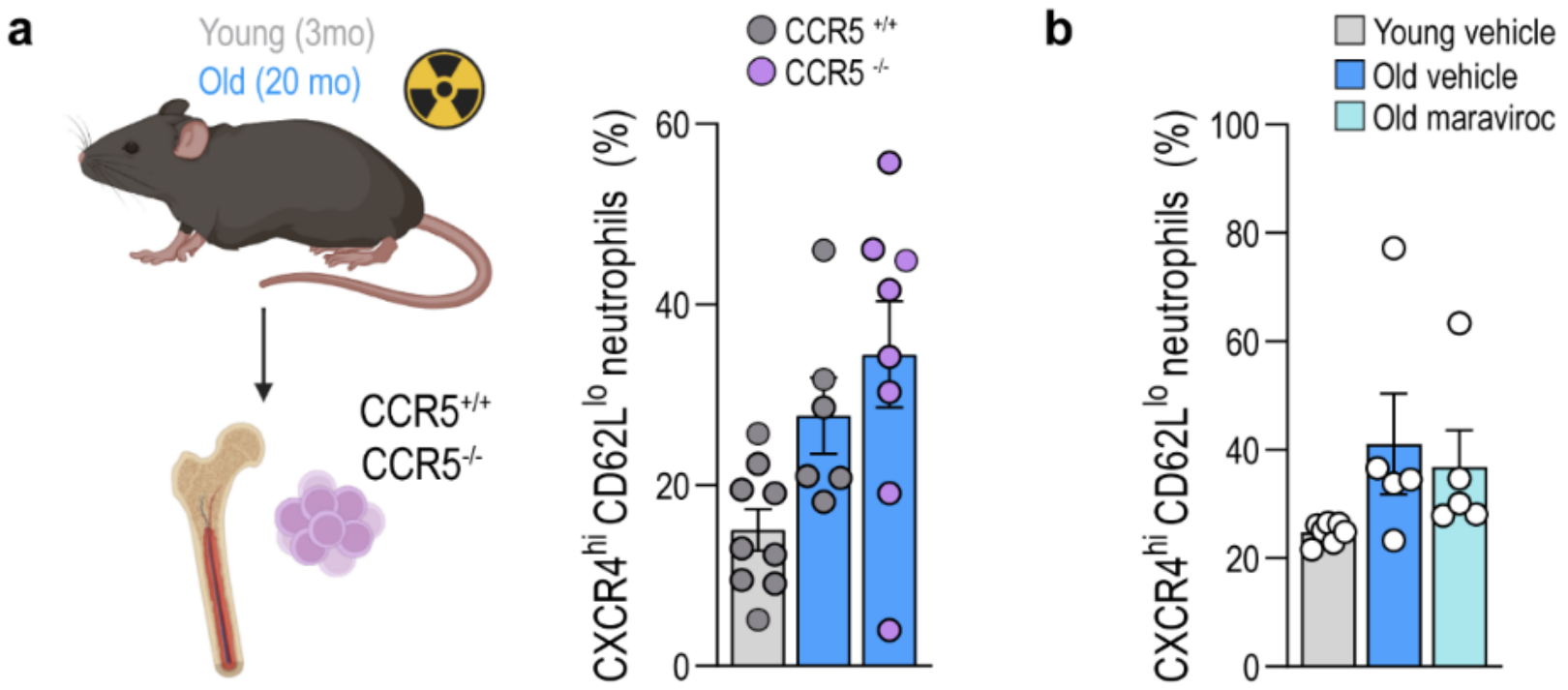

Fig. R1-1*. **a**, Schematic diagram depicting the bone marrow transplantation strategy of CCR5$^{+/+}$ or CCR5$^{-/-}$ bone marrow progenitors into lethally irradiated young (3 mo) or old (20 mo) recipient mice (**right**) and quantification of the percentage of blood CXCR4^{hi} CD62L^{lo} neutrophils 8 weeks post transplantation (**left**) (n= 9 young CCR5$^{+/+}$, 6 Old CCR5$^{+/+}$, 8 old CCR5$^{-/-}$). **b**, quantification of the percentage of blood CXCR4^{hi} CD62L^{lo} neutrophils in young (3 mo) or old (23 mo) recipient mice treated with vehicle or maraviroc for one month as indicated (n= 8 young vehicle, 5 old vehicle, 8 old maraviroc).*

*During the previous revision at Nature, in response to this reviewer, we performed experiments that discarded impaired BM homing of CXCR4^{hi}CD62Ll^{o} CD45.1 transferred neutrophils in aged mice. There are different potential reasons explaining why CXCR4^{hi}CD62L^{lo} neutrophils accumulate in aged mice. The first possibility, as indicated by the reviewer, is that higher GM-CSF levels in old mice would trigger the acquisition of these markers in neutrophils, which is supported by the equally increased GM-CSF levels upon Maraviroc treatment. Another non-mutually exclusive possibility, is that the intestinal microbiome might control the generation of CXCR4^{hi}CD62L^{lo} neutrophils (PMID: 26374999). Further studies should address whether this is the case in aged mice. Noteworthy, while the percentage of CXCR4^{hi}CD62L^{lo} neutrophils is not altered by maraviroc, this treatment induced a reduction of total circulating neutrophils (**Fig.5c**), which ultimately would derive in decreased CXCR4^{hi}CD62L^{lo} neutrophil counts.*

Referee #2 (Remarks to the Author):

I appreciate the efforts made by Mittelbrunn and colleagues to address the previous concerns raised by all reviewers. While there are arguable differences from previous published work (Shi et al Cell 2022), it is this Reviewers' opinion that the manuscript describes a cellular process/response with insufficient conceptual novelty to warrant publication in a broad multidisciplinary high-impact journal. Furthermore, the addition of human data is limited and does not appear to be statistically robust in showing accumulation of CD4+ CTLs in the context of human aging. Lastly, the claim of precise molecular mechanism, that mitochondrial dysfunction in CD4$^+$ T cells activates cGAS–STING signaling to induce CCL5 expression, is insufficiently demonstrated by experimental data. The authors have not convincingly demonstrated that CCL5 production exclusively from CD4+ T cells is the mechanism by which myelopoiesis is induced from hematopoietic progenitor cells.

We thank the reviewer for appreciating our effort in complementing the manuscript and for the long time spent in the evaluation of this manuscript.

Regarding the human data, the graph shows a modest but statistically significant correlation between the accumulation of cytotoxic CD4$^+$, but not CD8$^+$, T cells in the BM during ageing. We believe these findings provide novel information that has not been previously reported and therefore add further value and insight to the manuscript.

We provided a significant number of evidences that prove that mitochondrial dysfunction, at least in part, contributes to CCL5 production:

***1-** CD4$^+$ CTLs from aged mice and chronically stimulated CD4$^+$ T cells (which we extensively demonstrated that resemble CD4$^+$ CTLs in **Fig. 3** and **Extended Data Fig. 6**) exhibit typical signs of mitochondrial dysfunction such as decreased mitochondrial membrane potential, decreased basal and maximal OCR, increased mtROS levels and decreased expression of mitochondrial transcripts (**Fig. 3** and **Extended Data Fig.5**).*

***2-** CD4$^+$ T cells transferred from young mice into aged recipients show increased CCL5 levels (**Fig. 2**) and decreased mitochondrial membrane potential (**Fig. R2-1**).*

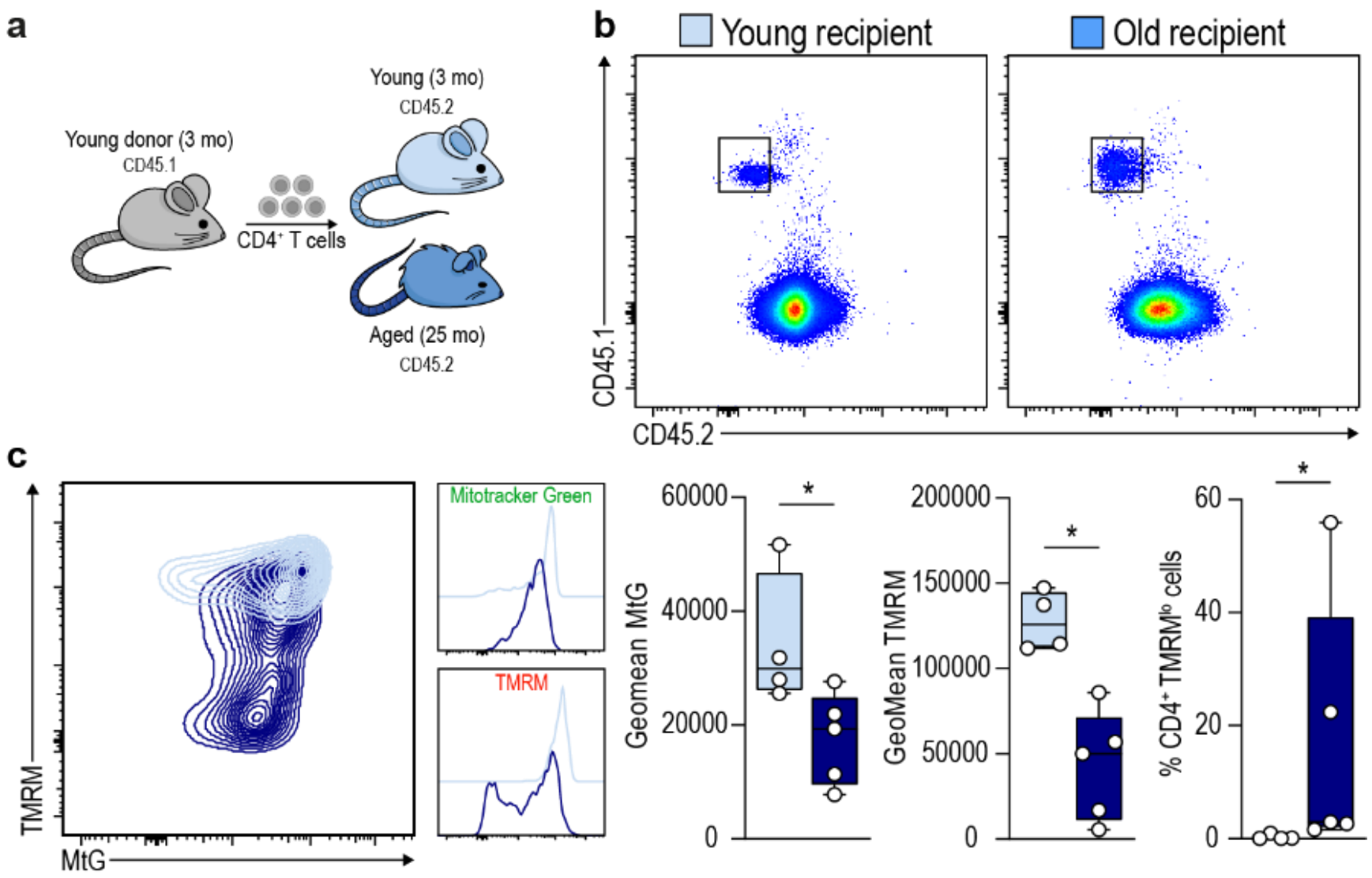

*Fig. R2-1. **a**, Schematic diagram depicting the adoptive transfer of CD45.1$^+$ T cells isolated from 3 months old (mo) mice into either young (3 mo) or old (24 mo) CD45.2$^+$ recipients. **b**, representative dot plots of transferred CD45.1$^+$ CD4$^+$ cells in the spleen of either young or old recipients. **c**, representative contour plot and histograms of TMRM and MtG (left) and quantification of the levels of MtG, TMRM and the percentage of CD45.1$^+$CD4$^+$TMRM^{lo} cells transferred into either young or old recipients (n = 4 young and 5 old mice).*

***3-** Pharmacological inhibition of the mitochondrial electron transport chain complexes at different levels (Rotenone for complex I and Antimycin for complex III) increases CCL5 levels in chronically stimulated CD4$^+$ T cells (**Fig. 3k**).*

***4-** Cytosolic DNA, which is an indicator of mitochondrial DNA release following mitochondrial damage (PMID: 25642965, PMID: 29472455), accumulates in CD4$^+$ T cells from aged mice and in chronically stimulated CD4$^+$ T cells (**Fig. 3l and m**).*

*5- Pharmacological activation of cGAS, which is activated as a consequence of mitochondrial damage (PMID: 25642965), increases CCL5 levels in chronically stimulated CD4$^+$ T cells (**Fig. 3q**).*

*6- Deficiency of Sting restores CCL5 levels in chronically stimulated CD4$^+$ T cells (**Fig. 3n**).*

*7- Upon co-adoptive transfer into same aged recipients, Sting deficient CD4$^+$ T cells display lower levels of CCL5 than control cells (**Fig. 3u**). Of note EOMES levels, the master regulator of CD4$^+$ CTLs, were not altered, suggesting that this is not due to impaired CD4$^+$ CTL differentiation (**Fig. 3v**).*

*8- Mice with a genetic induction of mitochondrial dysfunction in T cells exhibit increased levels of CD4$^+$ CTLs (**Extended Data Fig. 7**).*

*9- In vitro and in vivo treatment with drugs boosting mitochondrial function in T cells decrease the levels of CCL5 in continuously stimulated cells and in aged mice treated for 2 months These data were included in the third rebuttal letter in response to Reviewer 4 (**Fig. R4-7 of the previous revision round at Nature**).*

*10- To further demonstrate experimentally that mitochondrial dysfunction induces CCL5 expression in CD4$^+$ T cells, we included new data complementing **Extended Data Fig. 7**. Specifically, we analysed blood from mice with T cell–specific mitochondrial dysfunction caused by deletion of the ubiquinol-cytochrome c reductase gene (Uqcrq), which encodes complex III subunit VI. Uqcrq deficiency impairs complex III function, leading to reduced OCR and increased mtROS levels (PMID: 30626970; PMID: 40670617). At 8 months of age, CD4^{Cre}Uqcrq$^{fl/fl}$ mice showed increased CCL5 levels compared with control Uqcrq$^{fl/fl}$ littermates (**Fig.R2-2**). These findings further support that mitochondrial dysfunction in T cells can induce CCL5 expression.*

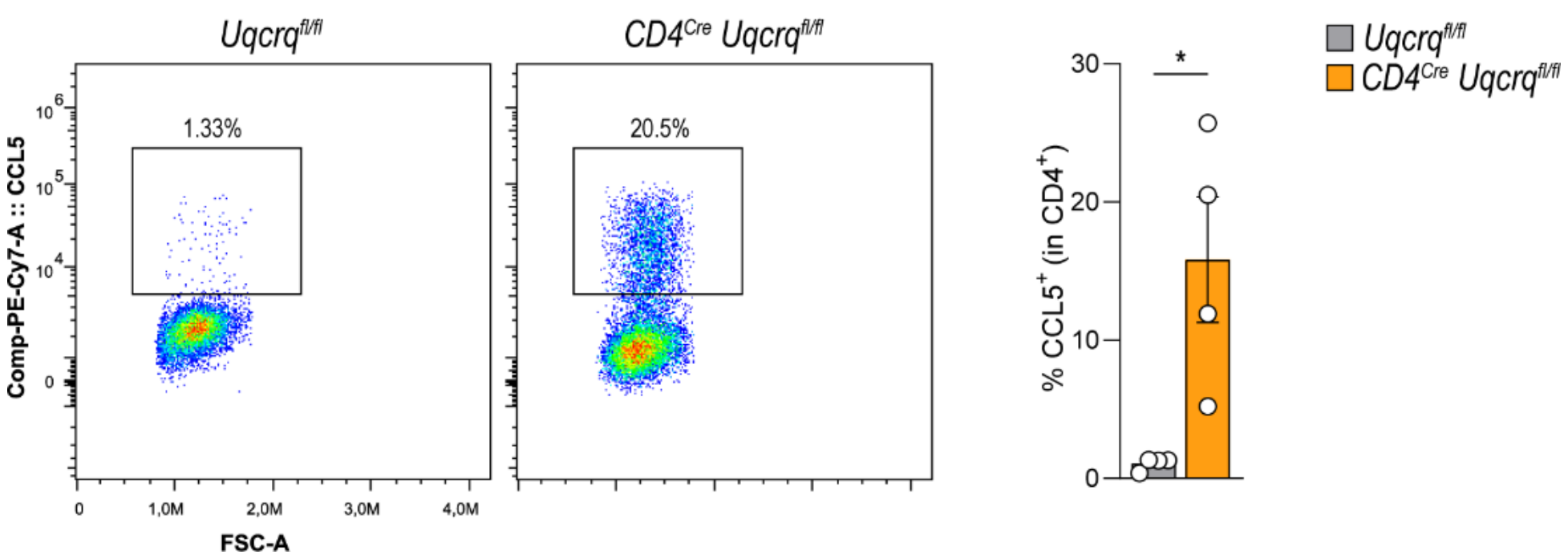

***Fig. R2-2**. **a,** Representative dot plot and quantification of the percentage of CCL5$^+$ cells in CD4$^+$ T cells from blood from 8 months old Uqcrq $^{fl/fl}$ and CD4^{Cre} Uqcrq $^{fl/fl}$ mice (n=4 Uqcrq $^{fl/fl}$ and 4 CD4^{Cre} Uqcrq $^{fl/fl}$ mice).*

We would like to emphasize that the accumulation of dysfunctional mitochondria in these cells does not preclude the activation of compensatory mechanisms that may allow them to cope with this deficiency, such as increased mitochondrial biogenesis, metabolic rewiring that reduces dependence on mitochondrial function, enhanced mitophagy, or upregulation of pro-survival pathways. Indeed, these cells have been reported to expand in supercentenarians (PMID: 31719197) and to retain functional cytotoxic capacity, including the ability to eliminate senescent cells (PMID: 37001502, PMID: 41057611). It is therefore plausible that these potentially beneficial functions favour the activation of pathways that promote the survival and persistence of these cells in aged individuals, even at the cost of deleterious side effects, such as those proposed in our manuscript. These possibilities have been discussed in the Discussion section.

<u>*Exclusive release of CCL5 by $CD4^+$ T cells*</u>

*Regarding the specific production of CCL5 by $CD4^+$ CTLs, in the previous round of revision we included re-analysis of CCL5 expression in publicly available scRNAseq datasets performed in either immune or mesenchymal cells from murine bone marrow (BM) samples from the Mouse Cell Atlas (PMID: 29474909) and the ImmGen (PMID: 18800157) databases. This analysis showed that CCL5 is mainly expressed by T cells, while other immune and non-immune BM cells express almost no CCL5 (**Fig. R2-4**).*

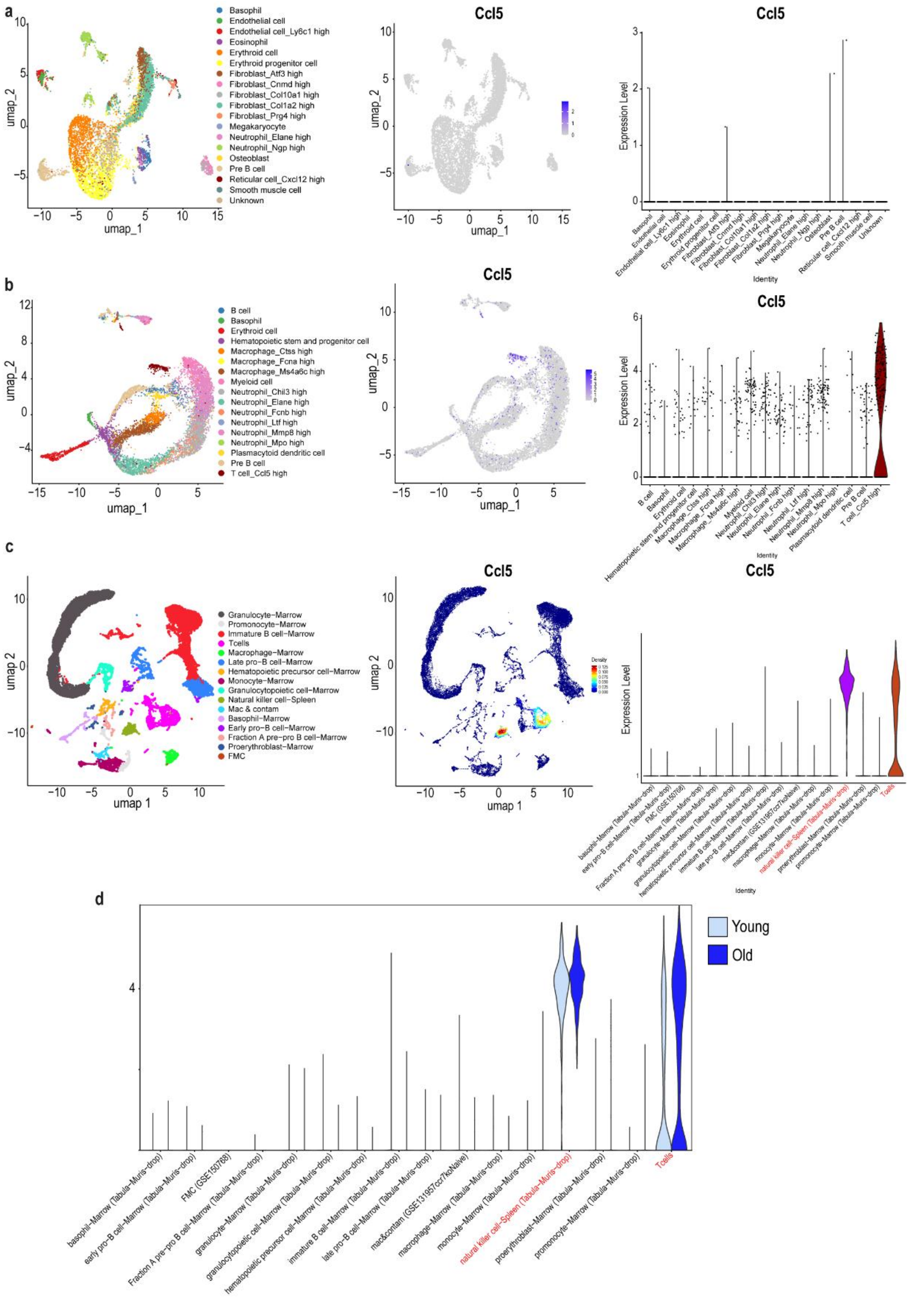

__Fig. R2-4__ __a__, UMAP clustering (left), map of expression of CCL5 (middle) and quantification (right) of data from scRNAseq of mesenchymal cells of the murine BM obtained from the Mouse Cell Atlas database. __b__, UMAP clustering (left), map of expression of CCL5 (middle) and quantification (rigth) of data from scRNAseq of immune cells of the murine BM obtained from the Mouse Cell Atlas database (PMID: 29474909). __c__, UMAP clustering (left), map of expression of CCL5 (middle) and quantification (right) of immune cells of the BM from young and old mice (PMID: 35613617). __d,__ quantification of CCL5 expression across immune cell BM clusters from young and old mice (PMID: 35613617).

*We also provided flow cytometry analysis confirming that CCL5 levels are not increased during ageing in immune cells of the BM, particularly in B cells, Ly6c^{hi} Monocytes and neutrophils (**Fig. R2-5**).*

*Furthermore, we provided in vitro experiments showing that **co-culture of aged CD4^{+} T cells with hematopoietic precursors** induces myeloid skewing and that this can be prevented by the addition of a soluble anti-CCL5 blocking antibody (**Fig.4**).*

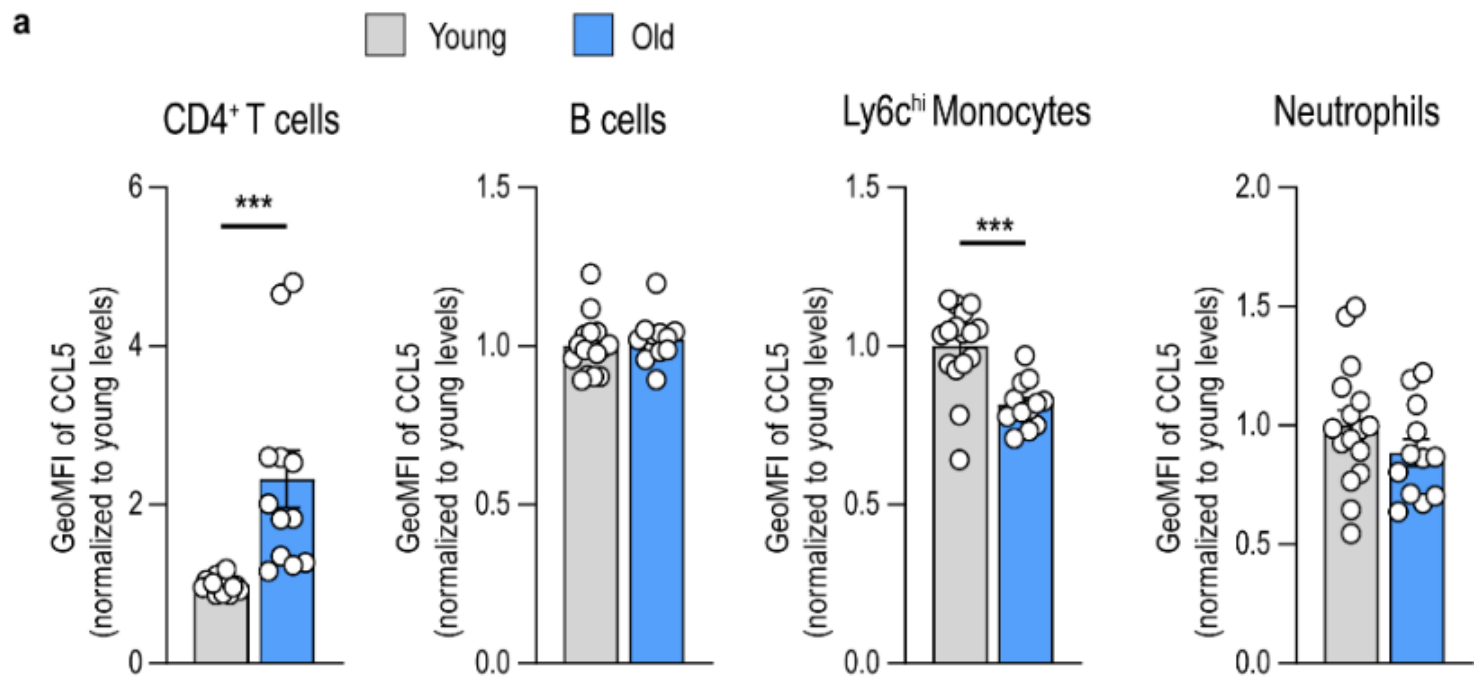

***Fig. R2-5**. **a**) CCL5 levels in blood from young and old mice (normalized to levels in young) in the indicated blood immune cell populations.*

*Finally, we have now performed a new set of experiments to rule out whether CD8^{+} cells could also contribute to myeloid skewing during aging. First, we analysed the presence of CD8^{+} T cells in the BM of aged mice finding increased numbers in aged mice (**Fig.R2-6a**). These cells also expressed high levels of CCL5 (**Fig.R2-6b**). Based on these evidences, we performed AT transfer of CD8^{+} T cells from aged mice into T cell deficient recipient mice and we analysed the outcome of haematopoiesis after 7-days (same conditions than in Fig.1). The analysis showed that neither myeloid progenitors nor HSC percentages or numbers were elevated upon transfer of aged CD8^{+} T cells (**Fig.R2-6c**). Accordingly, aged transferred CD8^{+} T cells neither increased the number of monocytes nor neutrophils in the BM (**Fig.R2-6c**) or the blood (**Fig.R2-6d**).*

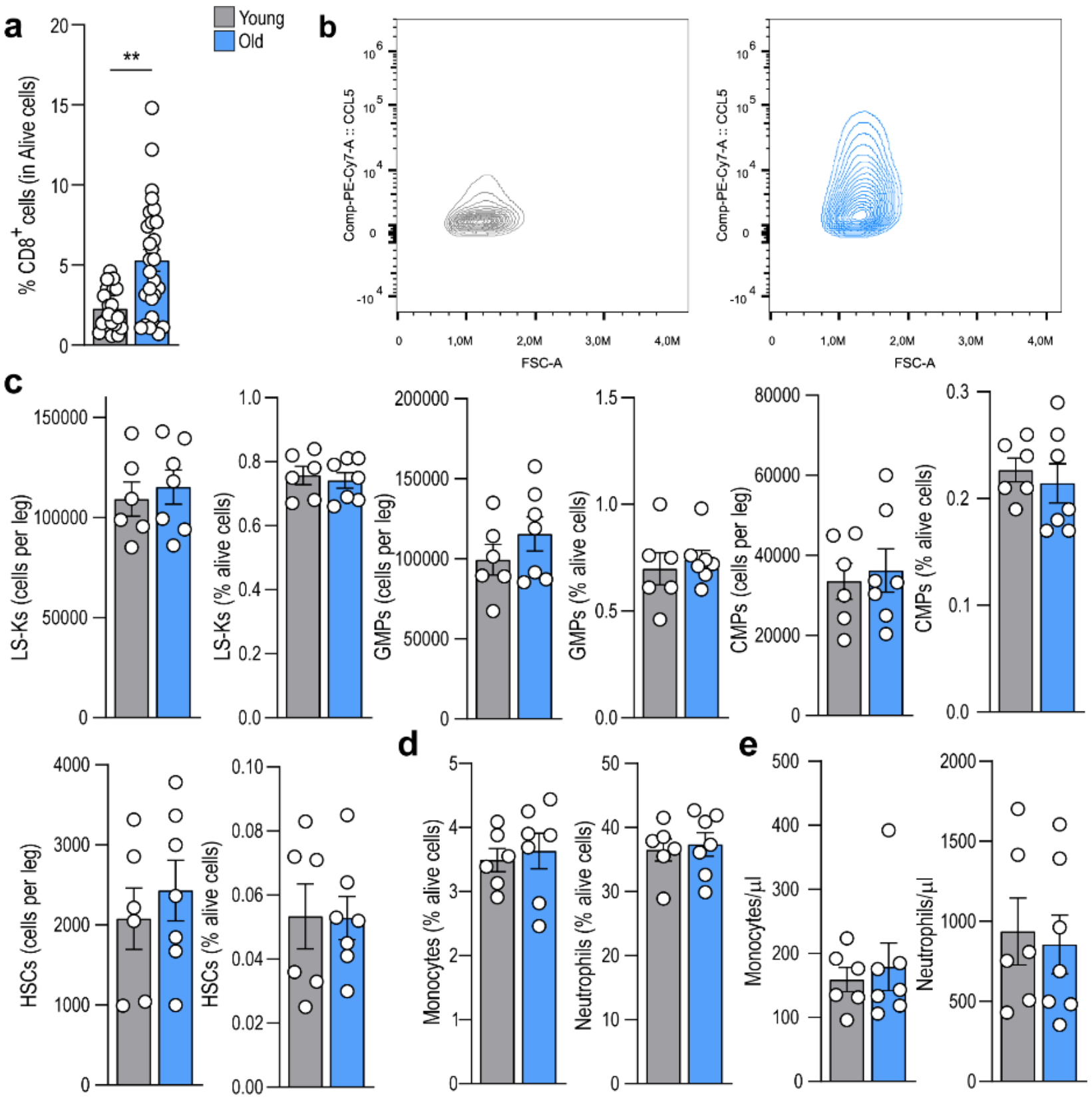

***Fig. R2-6**. **a**) Percentage of CD8^{+} cells in the BM of young (3mo) and old (24 mo) mice normalized to alive cells. **b**) representative dot plot showing increased expression of CCL5 in CD8^{+} cells in the BM of aged mice. **c**) Analysis of hematopoietic precursors in the BM of recipient mice 7 days after receiving an AT of 2.5 million CD8+ T cells isolated from either young or aged donor mice. **d**) Analysis of monocytes and neutrophils in the BM of recipient mice 7-days after the AT. **e**) Analysis of monocytes and neutrophils in the blood of recipient mice 7-days after the AT.*

Overall, these experiments suggest that aged CD8^{+} cells cannot directly induce myelopoiesis, at least in the absence of CD4^{+} cells. Addressing the effect of deleting CCL5 in every cell type that conforms the BM

microenvironment would require the generation of dozens of cell type-specific CCL5 knockout mouse lines and to study each of them at 24-months old, which we cannot afford at this stage.

Referee #3 (Remarks to the Author):

In the next round of revision, Gabandé-Rodríguez et al. provide some additional data aimed a mitigating my previous concerns. Overall, I am not convinced that their new experimental data suffice to support one of their key points:

We thank the reviewer for appreciating our effort in complementing the manuscript and for the long time spent in the evaluation of this manuscript.

previous Ad 1: The authors provide a couple of technical limitations for why no direct proof for the importance of BM T cells in regulating myelopoiesis is possible; instead, they now performed an experiment with intrafemoral transfer of T cells from the spleen and the lymph node. In this experiment they inject 2.5 million CD4 T cells, a number that seems excessive when compared to the 2 % of T cells reported in Figure 1d. The effect of this treatment seems modest especially when taking a closer look at the group of mice injected with young CD4 T cells - here, there seem to be 2 groups of 4 mice, one at a level identical to the ones injected with CD4 T cells from aged mice and a second group at a much lower level. Overall, this set of data does not rule out my concern that we might well be looking at a systemic rather than a bone marrow restricted effect - the latter being a major claim of the study.

Our data support that $CD4^+$ CTLs can promote myelopoiesis through CCL5 within the bone marrow. The preferential infiltration of T cells into the BM, together with the observation that co-culture of T cells with LSK cells induces myelopoiesis, led us to conclude that BM-resident T cells drive this process. Trying to assess previous concerns of the reviewer, we performed interfemoral injection of $CD4^+$ T cells, which resulted in a significant induction of myelopoiesis. However, as noted by the reviewer, this approach has inherent limitations.

*While our results demonstrate that T cell–mediated induction of myelopoiesis depends on CCL5 (**Fig. 4**) rather than on systemic inflammation, and the simplest explanation is that $CD4^+$ CTLs that accumulate in the bone marrow are the ones mediating bone marrow changes in HSCs, we cannot formally exclude that CCL5 produced by $CD4^+$ CTLs located outside the BM may also contribute to this effect. To ensure the accuracy of our claims we have changed the title of the manuscript and avoided directly linking the accumulation of these cells in the BM to a local induction of myelopoiesis throughout the text.*

previous Ad Fig 1: I am somewhat concerned that the authors provided a set of data in the last round of revision in response one of my questions when they now claim aspects of sample size may limit the robustness of these data. They claim that small changes in proportions may be different on absolute counts and refer to Extended Data Fig. 8e - yet, this panel does not show neutrophil counts; they also claim a phenotypic resemblance when comparing scRNAseq and FACS data - here they refer to Extended Data Fig. 1c and Fig. 2d, again both not displaying FACS data; beyond providing wrong references, the authors do not seem to be aware of the notorious mismatch of mRNA and protein expression in neutrophils; unless CITE-seq data are at hand such conclusions cannot be drawn.

*We would like to correct an error in our previous rebuttal: we mistakenly referred to Extended Data Fig. 8e instead of Extended Data Fig. 9e (now **Extended Data Fig. 7e** in the revised manuscript) when discussing neutrophil counts comparing young versus aged, and $Tfam^{fl/fl}$ versus $Tfam^{fl/fl}CD4^{Cre}$ mice.*

Regarding the removal of the data on neutrophil phenotype characterization, we believe that the manuscript became excessively long during the successive rounds of revision. We have deleted these data in an effort to focus the manuscript without compromising any of its conclusions. We would also like to stress that all experimental concerns related to this section were nevertheless addressed experimentally during the third round of revision.

Referee #4 (Remarks to the Author):

Gabande-Rodriguez et al. have performed additional experiments to reply to reviewers criticisms and to strengthen their arguments. I agree that they have answered the potential pitfalls with neutrophil expression of CCR1. They have provided other evidence generally consistent with their model and interpretations, although there remain a number of issues that would require better and more solid experimental support than given so far.

We thank the reviewer for clarifying his/her main concern and for the time devoted to the evaluation of our manuscript.

Most importantly, the central issue and the key caveat not resolved by any experiment in this revision is whether the rebalancing of hematopoiesis by blocking CCR5 (not CCL5, as I have erroneously written in my original critique) is responsible for the improvement of certain parameters of healthspan in old mice reported following maraviroc administration. To re-state the argument I have made in my previous critique– their maraviroc intervention impact not only the HSC compartment, but also the entire animal for prolonged periods of time and so they cannot possibly discriminate between the impact of CCR5 in the bone marrow and on HSC, versus its peripheral effects in end organs (e.g. Fig. 2i shows increased presence of CCL5+ CD4 T cells in a variety of organs of old recipient mice) or systemic circulation that CCL5+ T cells may impart.

In their rebuttal, the authors have agreed that they could not temporally or spatially dissociate such effects (p8). They have argued the following: "To overcome this limitation, we performed bone-marrow chimera experiments using CCR5+/+ and CCR5−/− donors (Fig. 4), which demonstrate that CCR5 expression in hematopoietic progenitors is required for the increased myelopoiesis driven by transferred aged T cells." That may be so, although some of the effects are pretty discrete (e.g. the ones in Fig. 4f-j). What matters, however, is that this experiment does not establish the beneficial effect of the hematopoietic ablation of CCR5 (or of deletion of CD4 T cells or of CCL5 on CD4 T cells) on the healthy aging, as indicated in the title of the manuscript. This remains the most critical issue where the authors do not meet the burden necessary to prove their hypothesis. I do not see how this work can achieve the sufficient priority without providing such a proof. I have asked for an orthogonal independent experimental validation of the CCR5+ blockade, which has not been provided.

We recognize the important point raised by the reviewer. Unfortunately, we find that suggested orthogonal approaches (all requiring the generation of new transgenic mouse lines and aging them for 24 months) are not currently feasible due to:

1- ***Deletion of CCR5 in hematopoietic cells*** *would not absolutely resolve the question, as all immune cells derived from these progenitors would also lack CCR5, making it impossible to determine whether the effects of maraviroc (or of CCR5 deletion) are restricted to hematopoietic progenitors or extend to mature immune cells, a point repeatedly raised during the review process.*

2- ***Deleting CCL5 specifically in CD4⁺ T cells*** *would still not formally establish whether improvements in aging phenotypes are mediated through effects on myelopoiesis or through other systemic mechanisms.*

Our results clearly demonstrate that maraviroc reduces age-associated myelopoiesis and improves health outcomes. We agree that our findings do not exclude the possibility that part of the beneficial effects on aging may involve mechanisms beyond the reduction of myelopoiesis. Nevertheless, we believe that the translational relevance of these findings justifies their inclusion in the manuscript. In this line, we acknowledge that the previous title may have overstated the mechanistic conclusions. We have therefore revised the title to more accurately reflect our observations.

There are many other issues where one could go back and forth, and where the authors would be well advised to improve and solidify their experimental evidence supporting the claims. Some are mentioned below, but this is not an exhaustive list, because these arguments are secondary in the face of the main criticism above. These criticisms are as follows:

1. The authors are vacillating between their neutrophil data being important and being a distraction. As of now, this is very confusing and the authors need to decide is this part of the compelling story or not.

Regarding the removal of the data on neutrophil phenotype characterization, we believe that the manuscript became excessively long during the successive rounds of revision, and that eliminating these data will help improve the clarity and focus of the manuscript without compromising any of its conclusions. We would also

like to stress that all experimental concerns related to this section were nevertheless addressed experimentally during the third round of revision.

2. The impact of TCR specificity remains incompletely addressed. In Fig. 2j, the authors use wt and OT-II CD4 T cells from young mice in an adoptive transfer model, and show that both induce CCL5 in the old environment (there is no control with adult recipients here, or the data on CCL5 expression before transfer); the data with PD-1 expression 14 days post transfer are all over the place, with some recipients mice expressing more PD-1 on OT-1 and others (the majority) on wt cells, with others showing no difference. What that exactly tells us about TCR engagement in this case is truly unclear. More sophisticated experiments would be needed to address this issue, which I have raised in my prior critique. The authors have performed additional in vitro stimulations (Extended data 8), but the conditions of acute vs chronic stimulation have been poorly explained. How long was chronic stimulation going for, and was there any resting between stimulations? Continuous stimulation will cause exhaustion and/or apoptosis and may or may not be what occurs in vivo.

*To analyze the basal proportions of cells in control and OT-2 mice we have followed two complimentary approaches. Firstly, we have analyzed the proportion of CD4^{+} CTLs in total splenic CD4^{+} T cells isolated from young control and OT-II mice of 3 mo, which are equivalent to those used for the adoptive transfer experiments performed in Fig. 2. Young OT-II mice display basal levels of CD4^{+} CTLs that were comparable to control mice (**Fig. R4-1a**). Secondly, data shown in Fig. 2i–k derive from two independent experiments, each including n=5 aged mice. In the first experiment, we indeed included a control group of 6 young recipients. However, we detected very few transferred cells infiltrating the bone marrow of young recipients, consistent with our observations in Fig. 2h. For this reason, we opted to present data only from aged recipients in the main figure. As requested by the reviewer, we now provide below the percentages of control and OT-II CCL5^{+} and PD-1^{+} cells in the BM of young recipients, despite the low number of detected cells (**Fig.R4-2**).*

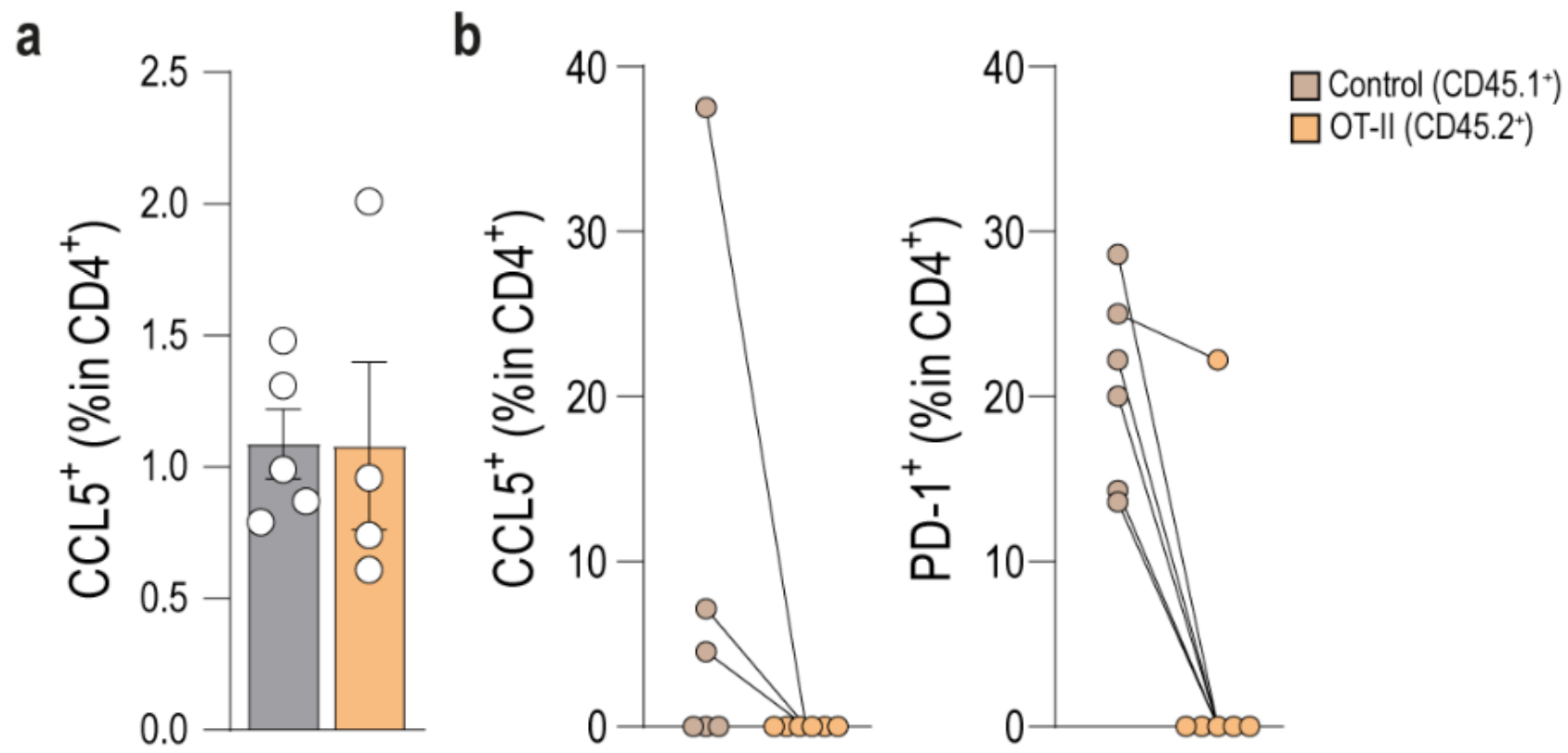

***Fig. R4-1**. **a**) Percentage of CD4^{+} CCL5^{+} cells in total CD4+ T cells purified from the spleen and lymph nodes from young (3 mo) control and OT-II mice (n = 5 control and 4 OT-II) mice). **b**) Percentage of control and OT-II CD4^{+} CCL5^{+}and CD4^{+} PD-1^{+} T cells in the BM of CD45.1.2 young recipient mice as performed in Fig. 2i (n = 6 young recipient mice).*

*Regarding previous criticisms on TCR engagement, the reviewer only raised concerns (**points 1 and 3 in revision 2**) regarding the necessity of demonstrating that a physiological stimulation induces CD4^{+} CTL differentiation as well as showing the levels of granzymes B and K, together with the expression of Blimp-1 and T-bet. All these questions were already addressed in the 3th revision (**Extended Data Fig. 6 and 8** of the present version of the manuscript). We also included schematic representations of the stimulation conditions for aCD3/aCD28 (**Fig. 3d**) and physiological stimulation (**Extended Data Fig. 6f**). As the reviewer suggests, we have now further clarified both protocols in the Materials and Methods section.*

*To address the reviewer's remaining questions; these protocols (aCD3/aCD28 and antigenic stimulations) did not include resting periods between stimulations and were maintained for 7-days. When we established the aCD3/aCD28 protocol to induce CD4^{+} CTLs in vitro, we actually compared the proposed protocol (protocol 1) with an alternative protocol that included resting periods (protocol 2) (**Fig. R4-2**). Briefly, in protocol 2, CD4^{+} T cells were initially activated with αCD3/αCD28 beads as in protocol 1. After bead removal, cells were cultured in complete RPMI supplemented with 10% FBS and IL-2 until day 5. At this point, cells*

were split into two groups: one group received repeated stimulation with αCD3/αCD28 beads for 48 h on days 5 and 12 in presence of IL-2 (CS protocol 2), whereas the control group was maintained with IL-2 alone (AS protocol 2). Thus, a total of three stimulation cycles were performed, and cultures were maintained for 18-days. Comparison of the protocols 1 and 2 at end point revealed a more efficient induction of EOMES$^+$CCL5$^+$ cells—defining CD4$^+$ CTLs (PMID: 31457092)— with protocol 1 (**Fig. R4-2**). These results led us to conclude that **sustained stimulation more effectively generates a population resembling CD4$^+$**

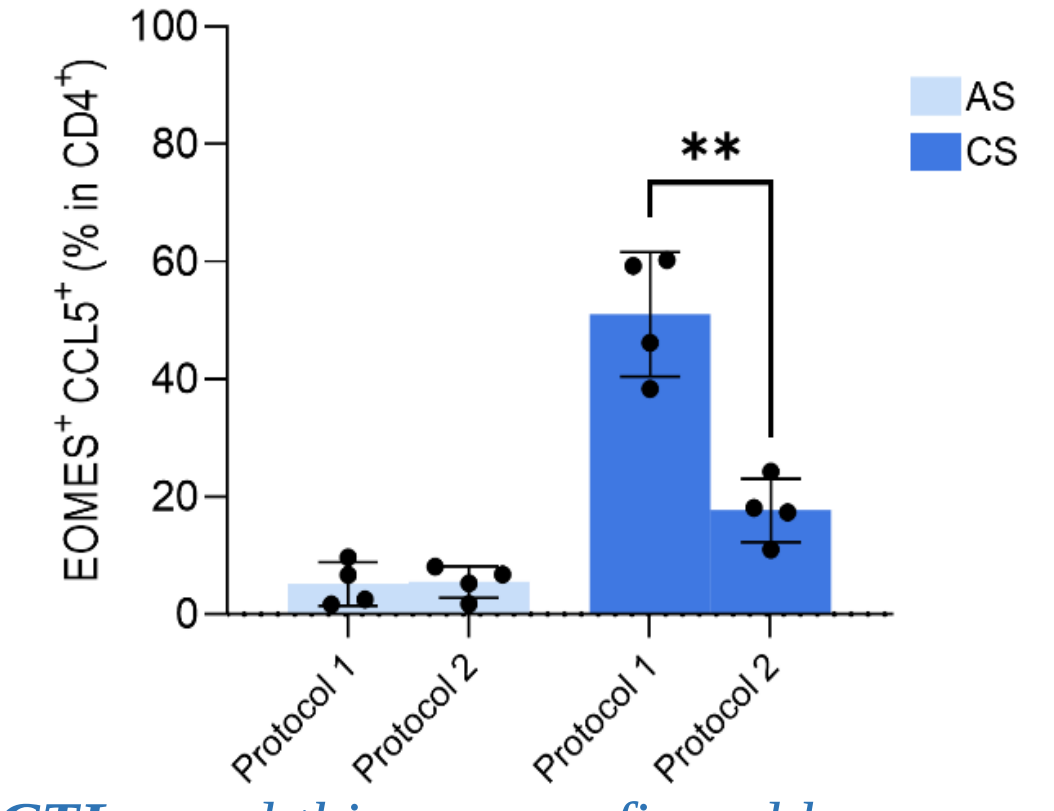

Fig. R4-3. Comparison between protocol 1 and protocol 2. Analyses were performed at the end of both protocols (day 7 for protocol 1, day 18 for protocol 2). The graph shows the frequency of CD4$^+$ EOMES$^+$ CCL5$^+$ T cells under AS and CS conditions at day 7 for protocol 1 and day 18 for protocol 2. (n=4 independent cultures).

CTLs, and this was confirmed by expression of several canonical CD4$^+$CTL markers in CS cells (**Extended Data Fig. 6**).

As noted by the reviewer, some protocols have described that continuous TCR stimulation, in combination with hypoxia, can induce exhaustion in CD8$^+$ T cells in vitro (PMID: 33398183), although this remains controversial in CD4$^+$ T cells. Indeed, autoreactive CD4$^+$ T cells can engage adaptive mechanisms that allow them to sustain chronic TCR stimulation without undergoing bona fide exhaustion, despite exhibiting features typically associated with exhaustion (PMID: 39396521). Consistent with this, we observed that CD4$^+$ CTLs in aged mice express low levels of canonical exhaustion-associated markers in vivo (**Extended Data Fig.4**), including PD-1, TOX, LAG-3, and TIM-3, in line with previous reports (PMID: 38077321, PMID: 33662046, PMID: 38698736, PMID: 32870971, PMID: 41030456). Supporting the conclusion that cells generated under our induction protocols are not functionally exhausted, we detected increased expression of T-bet—a key regulator of effector function in CD4$^+$ but not CD8$^+$ T cells (PMID: 11786644)—under both aCD3/aCD28 and physiological chronic stimulation conditions (**Extended Data Fig. 6d,g**). These cells also exhibited elevated expression of cytotoxic molecules and enhanced killing capacity (**Extended Data Fig. 6e**). To further discard exhaustion, we have now assessed cytokine production in vitro and in vivo. CS stimulated with PMA/Ionomycin for 4 h expressed increased levels of Ifng (**Fig-R4-3a**). Accordingly, CD4$^+$ CTLs stimulated with PMA/Ionomycin displayed increased levels of IFNg, and retained capacity to produce TNFa, than the rest of the clusters (**Fig-R4-3b**). These data support that both **CD4$^+$ CTLs, either in aged mice or in vitro, are not functionally exhausted**.

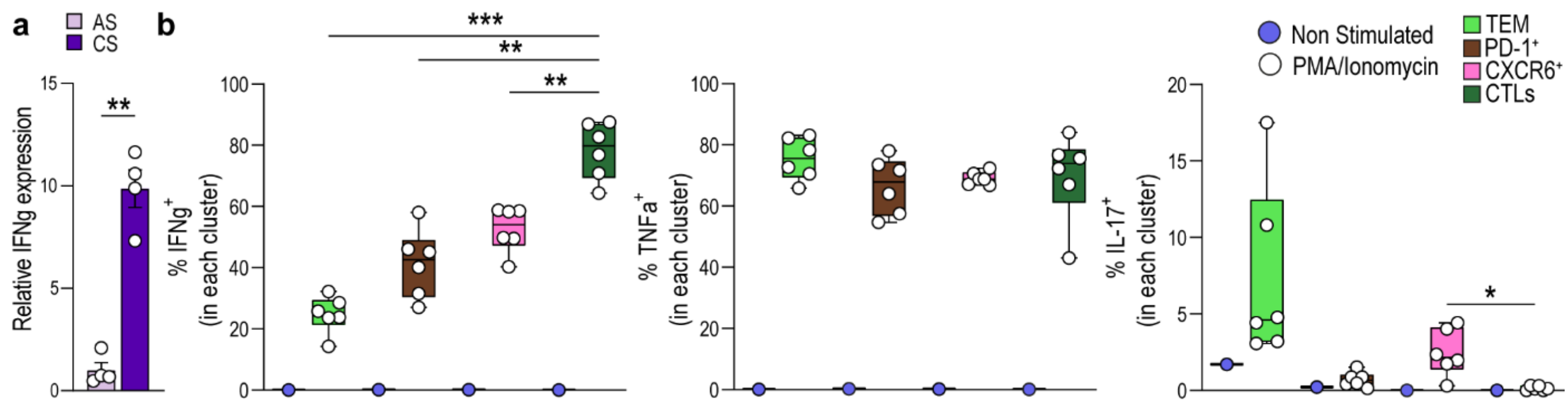

*Fig. R4-3. **a**) Relative mRNA levels of IFNg in PMA/Ionomycin stimulated AS and CS cells during 4 h (n = 4 independent cultures). **b**) Percentage of IFNg$^+$, TNFa$^+$ and IL-17$^+$ cells across indicated CD4$^+$ T cell subpopulations upon PMA/Ionomycin stimulation (n = 6 aged (24 mo) mice). Blue dots show percentage of cells positive for indicated cytokines in a non-stimulated control.*

*To further demonstrate that chronic TCR stimulation in vivo can induce a $CD4^+$ CTL phenotype, we studied an alternative approach based on a mouse model recently developed by Prof. Iannacone's lab (PMID: 40588520). In this mouse model, $CD4^+$ T cells express a transgenic TCR that exclusively recognizes the S protein of the envelope of the hepatitis B virus (HBV) (Env126 mice). Env126 mice are then bred onto a $Rag1^{-/-}$ background to prevent the development of polyclonal endogenous T cells. Env126 $CD4^+$ T cells can be activated through TCR stimulation in vivo by transferring naïve Env126 $CD4^+$ T cells into $Rag1^{-/-}$ mice that are subsequently infected with a recombinant vesicular stomatitis virus (VSV) expressing the HBV envelope glycoprotein (rVSVEnv) (PMID: 40588520). This constitutes an invaluable model to understand whether physiological continuous TCR stimulation (7-days) can induce $CD4^+$ CTL differentiation. Notably, scRNAseq analysis comparing naive and in vivo stimulated Env126 identified two clusters of effector $CD4^+$ T cells (clusters 1 and 2), which were absent in naïve cells from Env126 mice (cluster 0) (**Fig.R4-4a,b**). Clusters 1 and 2 exhibited high expression of canonical $CD4^+$ CTLs markers such as Ccl5, Eomes Nkg7, Gzma, Gzmb, Gzmc, Gzmk, Gzmm and Cxcr3, together with markers of effector function such as Tbx21 (T-bet), Tnf and Ifng. These clusters also presented upregulation of canonical exhaustion markers such as Lag-3, Tim-3 (Havcr2), PD-1 (Pdcd1) or Tigit (**Fig.R4-4c**).*

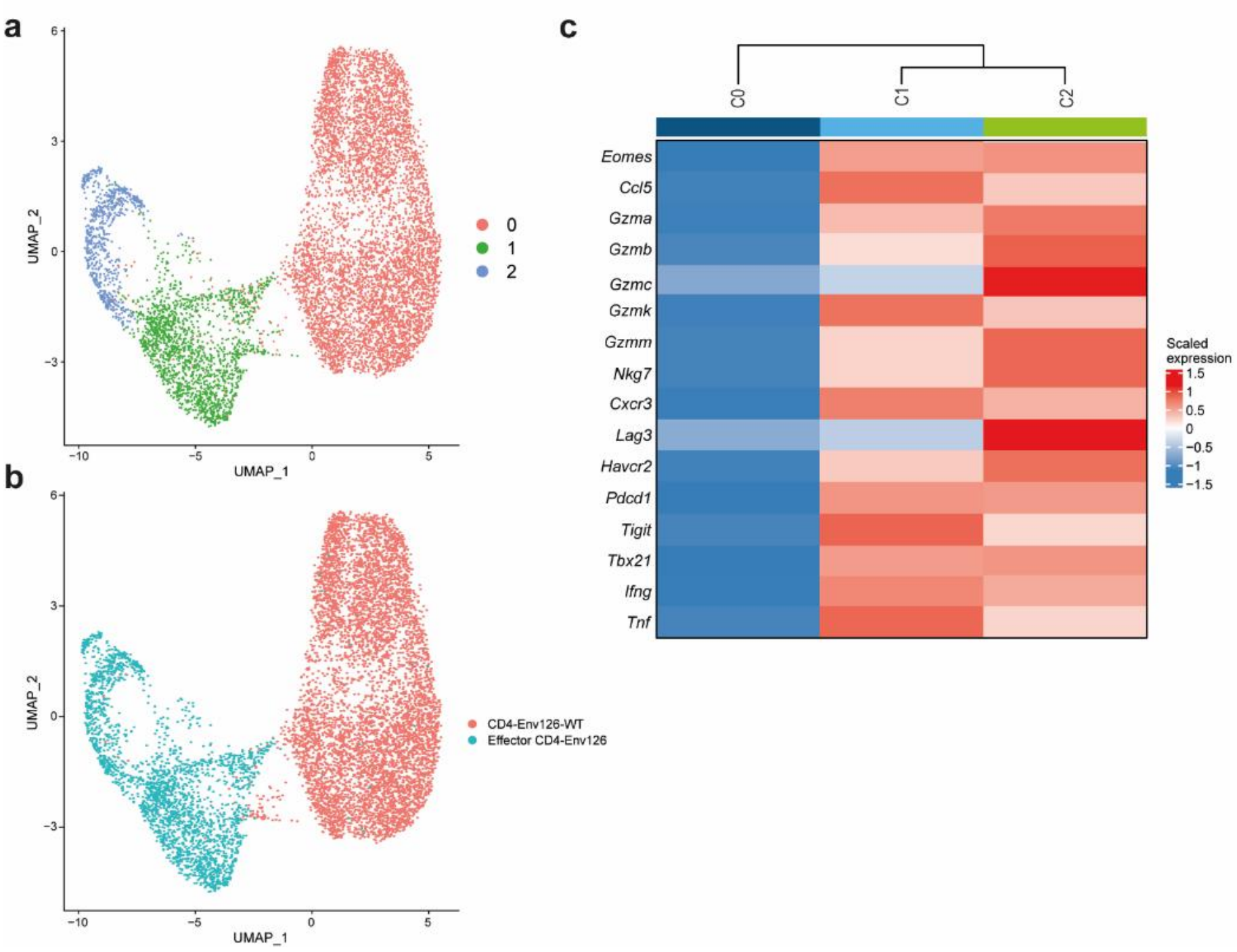

***Fig. R4-4**. **a**) UMAP showing the distribution of the clusters in Env126 T Naïve and Env126 T Effector cells. **b**) UMAP showing the differential distribution of both populations. **c**) heat map showing the expression of indicated gene transcripts in clusters 0, 1 and 2.*

Collectively, these data support that the cells identified in our manuscript within the $CCL5^+$ cluster correspond to the population consistently referred to as $CD4^+$ CTLs in the literature. Furthermore, this data supports that in vitro and in vivo continuous TCR stimulation promote $CD4^+$ CTLs clonal expansion. Critically, these cells do not exhibit functional exhaustion despite the expression of canonical exhaustion-associated markers, in line with previous reports (PMID: 38077321, PMID: 33662046, PMID: 38698736, PMID: 32870971, PMID: 41030456). Given this coexistence of cytotoxic and exhaustion-associated features, it is possible that $CD4^+$ CTLs represent an intermediate state preceding terminal exhaustion. However, this distinction does not affect the conclusions of our study, as our findings are independent of the precise differentiation state of these cells. This possibility nevertheless warrants further investigation in future studies.

3. The authors have argued that in the bone marrow most of the CCL5 expression segregates with CD4 T cells, and have presented new data in Fig. R4-2 to that effect. CD8 T cells were not analyzed or shown for some reason.

*We thank the reviewer for raising this important point. We initially did not analysed CD8⁺ T cells because they were absent in our set of experiments (AT of CD4⁺ T cells into T cell deficient mice, and in vitro co-culture of CD4 T cells with hematopoietic precursors). However, it is true that CD8⁺ T cells have been linked to haematopoiesis induction in different conditions including viral infections (PMID: 24561082 and more recently in bioRxiv PMID: 41509281). To study whether they could have a role in inducing myeloposiesis during ageing, we first analysed the presence of CD8⁺ T cells in the BM of aged mice finding increased numbers of these cells in aged mice (**Fig.R4-5a**). Aged BM CD8⁺ cells also express high levels of CCL5 (**Fig.R4-5b)**. Based on this we studied whether they also contribute to induce myelopoiesis in aged mice by performing AT experiments of CD8⁺ T cells isolated from aged mice into T cell deficient mice. We analysed the outcome of haematopoiesis after 7-days (same conditions than AT of CD4 T cells from aged mice in Fig.1 and Fig. 4) and we found that neither myeloid progenitors nor HSC percentages or numbers were altered upon transfer of aged CD8⁺ T cells (**Fig.R4-5c**). CD8⁺ T cells neither increased the number of monocytes or neutrophils in the BM (**Fig.R4-5c)** or the blood (**Fig.R4-5d**).*

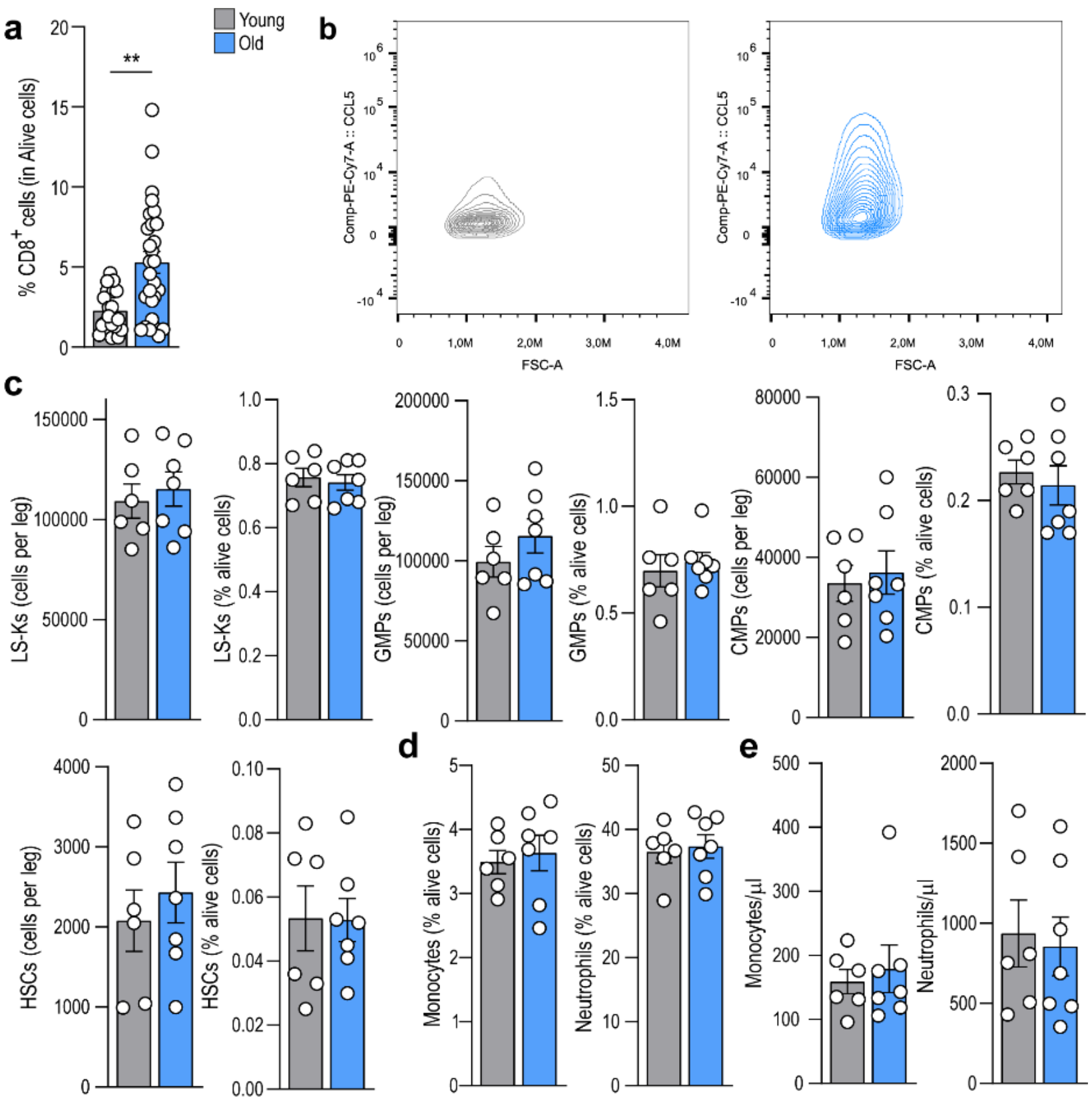

***Fig. R4-5**. **a**) Percentage of CD8⁺ cells in the BM of young (3mo) and old (24 mo) mice normalized to alive cells. **b**) Representative dot plot showing increased expression of CCL5 in CD8⁺ cells in the BM of aged mice. **c**) Analysis of hematopoietic precursors in the BM of recipient mice 7 days after receiving an AT of 2.5 million CD8+ T cells isolated from either young or aged donor mice. **d**) Analysis of monocytes and neutrophils in the BM of recipient mice 7-days after the AT. **e**) Analysis of monocytes and neutrophils in the blood of recipient mice 7-days after the AT.*

Overall, these experiments suggest that aged CD8⁺ cells cannot directly induce myelopoiesis, at least in the absence of CD4⁺ cells.

4. Prior critique point #4 –"assigning the cytotoxic phenotype" – and in particular "Are these cells functionally cytotoxic, and could they be also potentially eliminating HSC's lymphoid progeny?" The authors performed some cytotoxicity experiments, but none of them used bone marrow-derived CD4 T cells that this manuscript studies. If it is difficult to isolate these cells, in vivo cytotoxic assay can be performed with labeled target cells.

We thank the reviewer for raising this interesting point.

In our manuscript, we assigned a cytotoxic identity based on established nomenclature indicating that EOMES and CCL5 are CD4⁺ CTLs defining markers (PMID: 31457092, PMID: 41057611), and by the expression of cytotoxic marker including Nkg7, Ctsw Ctsd, granzyme K, and granzyme M (scRNA-seq; ***Extended Data Fig. 4b,c****), as well as perforin (****Fig. 2d****) and granzyme A and B (flow cytometry in aged mice;* ***Extended Data Fig. 4d****. Notably, the cytotoxic capacity of CD4⁺ CTLs cells towards different particular cell types has also been extensively reported in different tissues (PMID: 37001502, PMID: 41057611), including the bone marrow (PMID: 40106762). In addition, we demonstrated that chronically stimulated CD4⁺ T cells—either via αCD3/αCD28 stimulation or antigen presentation by B cells—recapitulate this CD4⁺ CTL signature and are capable of killing OVA peptide–presenting B cells in vitro (****Extended Data Fig. 6****).*

*To further substantiate the cytotoxic identity of these cells, we have now analyzed an independent cohort of mice and confirmed that CD4⁺ CTLs in the BM of aged animals display the highest levels of surface LAMP-1/CD107a, a surrogate marker of cytotoxic granule degranulation (PMID: 23632890), across all clusters (****Fig. R4-6****).*

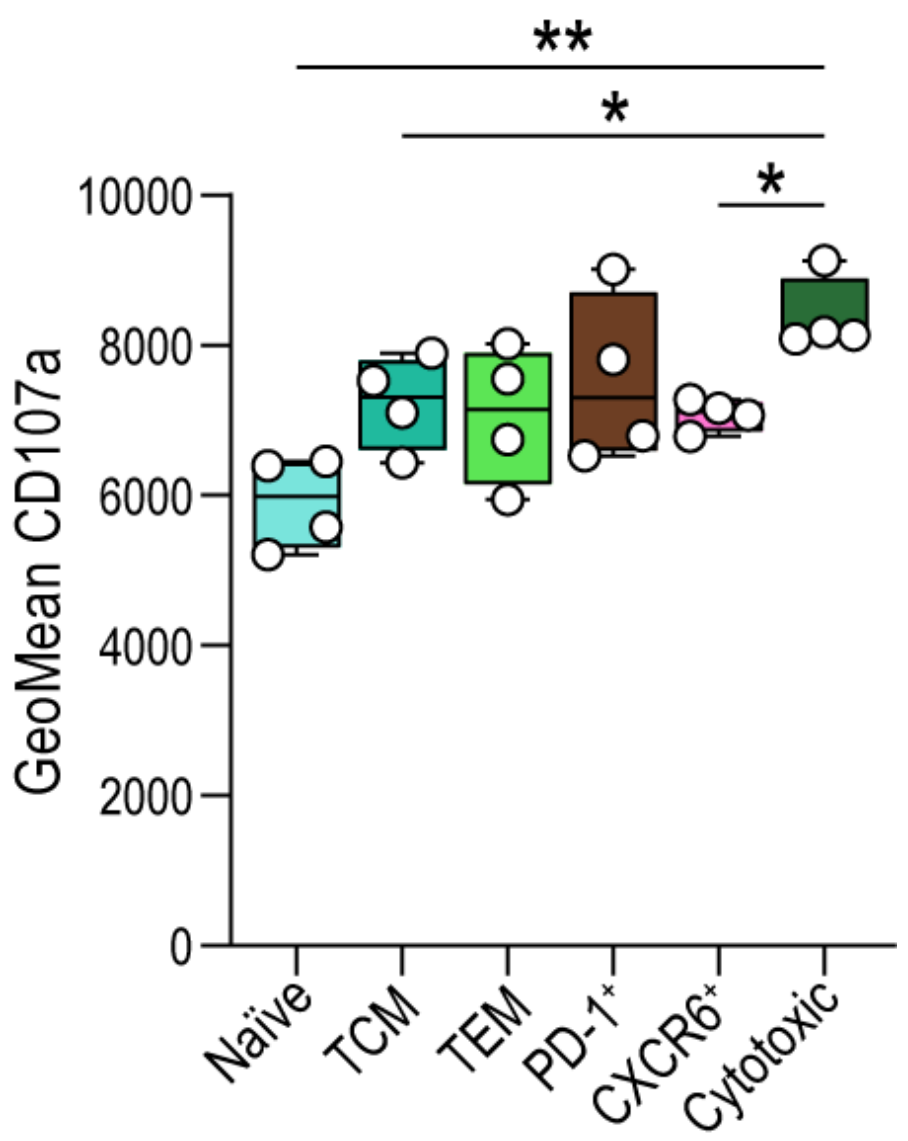

Fig. R4-6*. Geomean levels of plasma membrane bound Lamp-1/CD107a in CD4⁺ T cells classified within the indicated sub-populations (identified by manual gating) in the bone marrow of old (24 mo) mice. (n=4 mice).*

Regarding cytotoxicity against lymphoid progenitors, this is an interesting hypothesis that could explain a complementary mechanism behind increased myelopoiesis. We present some pieces of evidence against this hypothesis;

1- we did not observe a reduction in common lymphoid progenitors (CLPs) in recipient mice transferred with aged T cells compared with mice transferred with young T cells or vehicle (neither in percentages; ***Fig. 4,*** *nor in cell numbers* ***Fig.R4-7****), arguing against a cytotoxic effect as the driver of myeloid skewing. Performing the proposed in vivo cytotoxicity assay using CLPs is technically very challenging. CLPs are a low abundant population (typically ~800–1,500 cells recovered per hind limb, and even fewer in aged mice), and their identification requires staining with multiple antibodies (****Extended Data Fig. 2****), necessitating prolonged use of a spectral flow cytometry cell sorter due to their low abundance—an instrument not available to us. Moreover, their homing capacity following intravenous injection, particularly after extensive antibody labelling, is uncertain.*

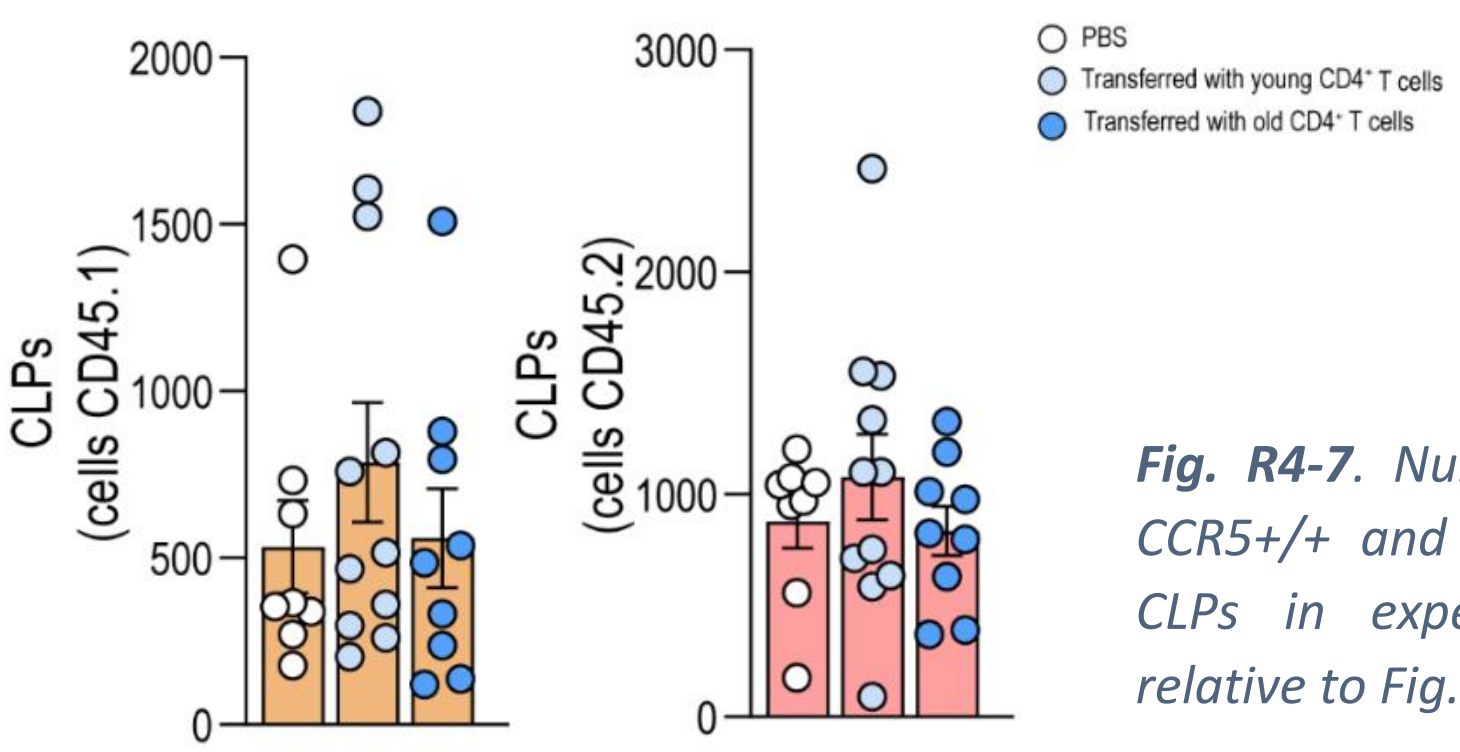

Fig. R4-7*. Number of CCR5+/+ and CCR5-/- CLPs in experiments relative to Fig. 4.*

*2- we have analyzed hematopoietic precursor populations in young control, aged control and aged T cell–deficient mice. Supporting that aged T cells induce myelopoiesis, we observed reduced numbers of myeloid progenitors (LSK⁻, CMPs and GMPs) and comparable numbers of HSCs (**Fig. R4-8**). Notably, the deficiency of T cells in aged mice did not restore the levels of lymphoid progenitors (**Fig. R4-8**). These findings are more consistent with a model in which aged T cells selectively promote differentiation toward the myeloid lineage, rather than directly mediating cytotoxic depletion of lymphoid progenitors.*

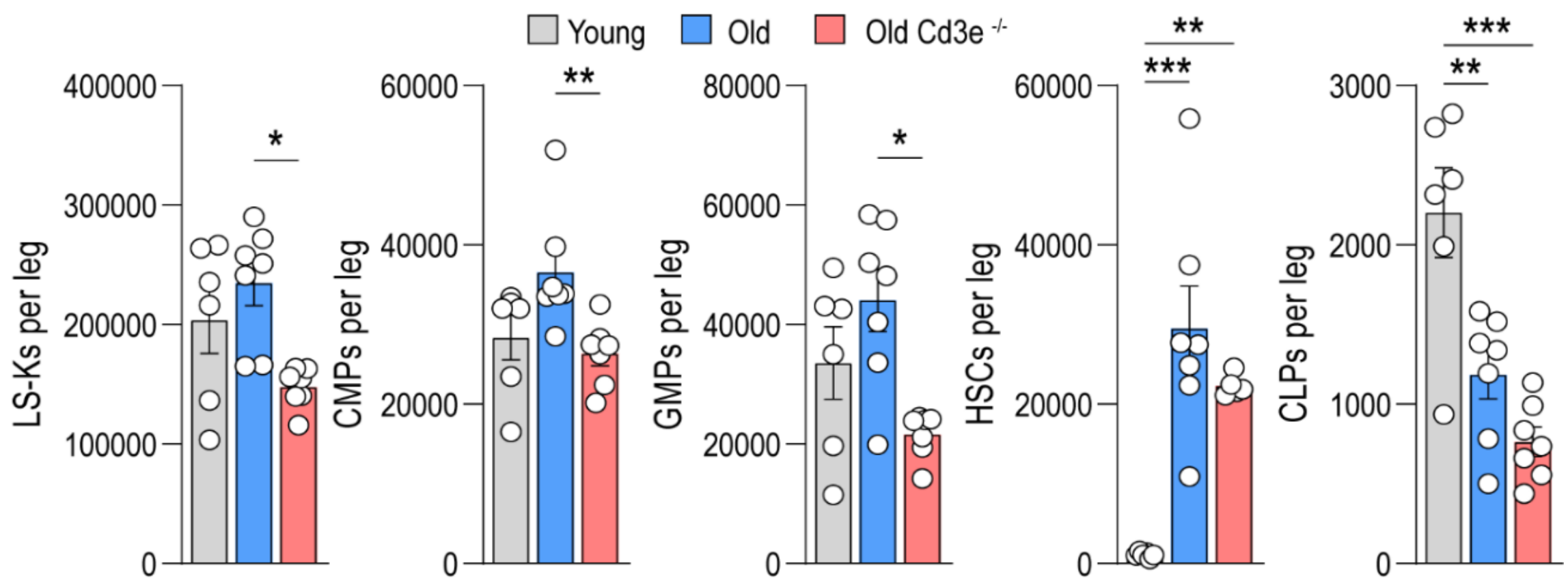

***Fig. R4-8**. Quantification of the number of indicated progenitors in the bone marrow of young (3 mo) old (24 mo) control mice and old (24 mo) T cell deficient mice (Cd3e$^{-/-}$). (n= 6 young mice, 7 old control mice, 7 old Cd3e$^{-/-}$ mice).*

*Finally, we also analyzed CCR5 expression across myeloid and lymphoid progenitors from young and aged mice, finding that CCR5 is upregulated in aged myeloid, but not lymphoid progenitors (**Fig.R4-9**), adding to several evidence provided within the manuscript (**Fig. R4-9; Fig. 4c–j** and **k–m**), further supporting a model in which a CCL5–CCR5 axis drives the selective expansion of myeloid progenitors.*

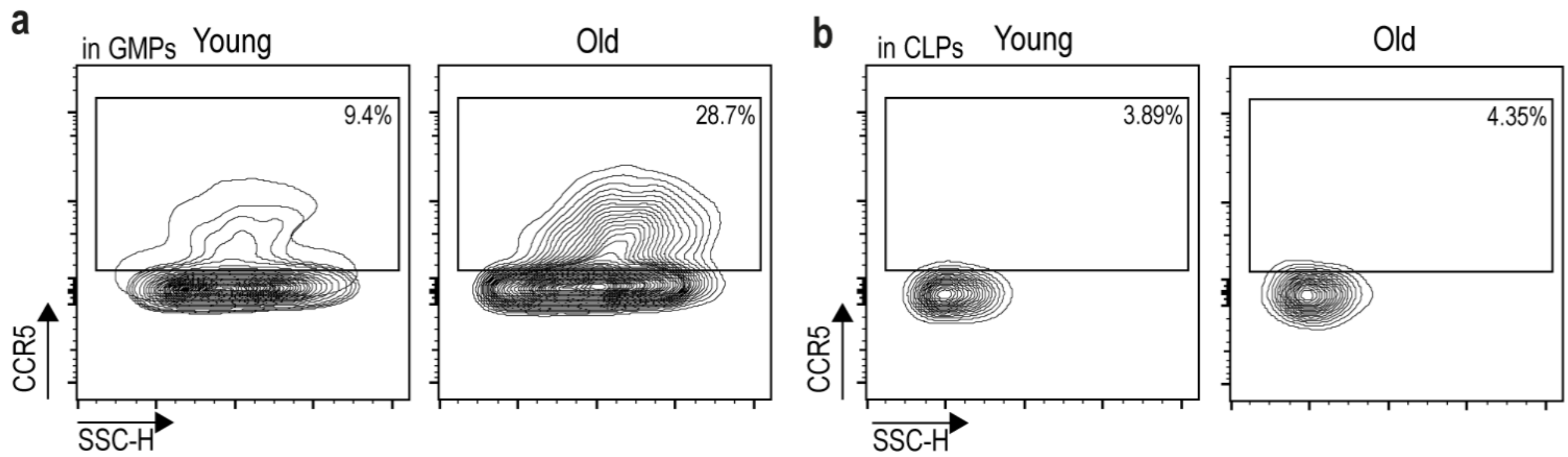

***Fig. R4-9**. **a**) Representative contour plot showing increased expression of CCR5 in GMPs in young (3 mo) and old (24 mo) mice (for quantification please see Fig. 4 of the main manuscript). **b**) Representative contour plot showing almost null expression of CCR5 in CLPs in young (3 mo) and old (24 mo) mice.*

Altogether, these results support the conclusion that aged T cells promote age-associated myeloid skewing through a CCL5–CCR5 axis, rather than by reducing the levels of lymphoid progenitors in aged mice.

Other criticisms:

•Is lymphopoiesis also reduced in the experiments 1 f.?

*Equivalent information to that seeked by the reviewer (the effect on CLPs under adoptive transfer of young/aged CD4^{+} cells) is included in experiments provided in **Fig. 4**, where we performed a fully comparable analysis including IL-7R and complete CLP gating in both CCR5$^{+/+}$ (comparable to **Fig.1j**) and CCR5$^{-/-}$ progenitors, finding no effect of aged CD4^{+} cells on the percentage of CLPs (**Fig. 4g**) or in absolute numbers (**Fig. R4-7**). The absence of effect of aged T cells on lymphopoiesis has been further substantiated by experiments on this revision (**Fig. R4-8**). Given that these datasets directly address the reviewer's point and*

that repeating the experiment would involve extensive animal use without adding new biological insight, we respectfully chose not to duplicate this set of experiments.

• Line 92, the term “faulty immune cells” is both imprecise and unscientific. What do the authors really mean here? Moreover, that sentence is long, convoluted, and difficult to follow due to poor syntax.

This sentence has been corrected in the new version of the manuscript.

• Figure 1 text description in the paper: What is a "fresh" neutrophil? This is particularly pertinent in light of the longevity of neutrophils and of the finding that transferred neutrophils can adopt “aged” phenotype upon transfer into older environment (Extended data Fig. 3).

We apologize for the misunderstanding. We again tried to follow the nomenclature established in the field. However, as noted above, we have decided not to include these data in the current version of the manuscript.
