# Supplementary material for: Cytotoxic CD4+ T cells induce age-associated myelopoiesis through CCL5–CCR5 signaling
Source: Nat Aging. 2026 Aug 26;6(10):2110–25. doi: 10.1038/s43587-026-01209-9 (PMC13661277; doi:10.1038/s43587-026-01209-9)
Supplement: Supplementary file 3 [file 43587_2026_1209_MOESM3_ESM.pdf]

Supplementary Table. 1

Extracellular antibodies

Antigen	Fluorochrome	Clone/#Catalog	Supplier	Dilution
CD8	BUV615	53-6.7	Thermo Fisher Scientific	1:500
CD127 (IL-7R)	BUV737	SB/199	BD Horizon	1:200
Ly6G	BV421	1A8	Biolegend	1:400
CD186 (CXCR6)	BV421	SA051D1	Biolegend	1:100
Streptavidin	BV421	405225	Biolegend	1:500
Lineage	BV421	133311	Biolegend	1:200
CD38	Pacific Blue	90	Biolegend	1:500
TIM3	BV480	5D12/TIM-3	BD Biosciences	1:200
CD19	V450	1D3	Tonbo Biosciences	1:400
CD45.2	V500	104	Tonbo Biosciences	1:400
CD16/32	BV510	93	Biolegend	1:100
CD44	BV570	IM7	Biolegend	1:200
CD223 (LAG3)	BV650	C9B7W	Biolegend	1:100
CD62L	BV711	MEL-14	Biolegend	1:400
Ly6C	BV785	HK1.4	Biolegend	1:400
B220	FITC	RA3-6B2	Tonbo Biosciences	1:200
CD34	FITC	RAM34	BD Biosciences	1:100
CD62L	FITC	MEL-14	Tonbo Biosciences	1:400
FoxP3	FITC	Fjk-16s	Invitrogen	1:400
Ly6C	PerCP Cy5.5	HK1.4	Biolegend	1:400
CD44	PerCP Cy5.5	IM7	Tonbo Biosciences	1:200
CD45.2	PerCP Cy5.5	104	Biolegend	1:400
PD1	PerCP eFluor710	J43	Thermo Fisher Scientific	1:200
CD135 (FLT3)	PerCP eFluor710	A2F10	Thermo Fisher Scientific	1:200
CD8	PE	53-6.7	Tonbo Biosciences	1:400
CD117 (cKit)	PE	ACK2	Tonbo Biosciences	1:200
NKG2D	PE-Dazzle594	CX5	Biolegend	1:200
CD25	PE Cy5	PC61	Biolegend	1:400
CD195 (CCR5)	Alexa Fluor 647	HEK/1/85a	Biolegend	1:100
Ly6G	PE-Cy7	1A8	Biolegend	1:400
SLAM (CD150)	PE-Cy7	TC15-12F12.2	Biolegend	1:200
Annexin V	PE-CF594	RUO	BD Biosciences	1:400
CX3CR1	APC	SA011F11	Biolegend	1:200
CD11b	APC	M1/70	Tonbo Biosciences	1:200
CD48	APC	HM48-1	Biolegend	1:200
CD11b	Red-Fluor 710	M1/70	Tonbo Biosciences	1:200
Sca-1	APC-Cy7	D7	Biolegend	1:200
CD45	APC-Cy7	30-F11	Biolegend	1:400
CD45.1	APC-Cy7	A20	Tonbo Biosciences	1:400
CD279 (PD-1)	APC-Fire750	29F.1A12	Biolegend	1:400
CD19	APC-efluor780	eBio1D3	ThermoScientific	1:200

CD4	APC Fire810	Gk1.5	Biolegend	1:1000
B220	Biotin	RA3-6B2	Tonbo Biosciences	1:200
Ter119	Biotin	TER-119	Tonbo Biosciences	1:200
Gr-1	Biotin	RB6-8C5	Tonbo Biosciences	1:200
CD11b	Biotin	M1/70	Tonbo Biosciences	1:200
CD11c	Biotin	M418	Tonbo Biosciences	1:200
CD4	Biotin	GK1.5	Tonbo Biosciences	1:200
CD8	Biotin	53-6.7	Tonbo Biosciences	1:200
CD3	Biotin	145-2C11	Tonbo Biosciences	1:200

Intracellular antibodies

Antigen	Fluorochrome	Clone/#Catalog	Supplier	Dilution
Blimp-1	BV421	5-E7	BD Biosciences	1:200
FoxP3	FITC	Fjk-16s	Invitrogen	1:150
Granzyme A	Alexa Fluor 488	3G8.5	Santa Cruz	1:100
T-bet	Real Blue 780	04-46	BD Biosciences	1:100
CCL5	PE	2E9/CCL5	Biolegend	1:400
Granzyme K	PE	GM6C3/SC-56125	Santa Cruz	1:100
CCL5	PE-Cy7	2E9/CCL5	Biolegend	1:400
EOMES	PE-Cy7	Dan11mag	Invitrogen	1:200
Granzyme B	PE-Cy7	NGZB	eBiosciences	1:100
Perforin	APC	S16009A	Biolegend	1:200
T-bet	APC	4B10	Biolegend	1:100
TOX	eFluor660	TRX10	Invitrogen	1:300
Granzyme B	Alexa Fluor 700	GB11	BD Biosciences	1:100
BHLHE40	Alexa Fluor 700	#NB100-1800AF700	Novus Biologicals	1:200
