# Supplementary material for: Cytotoxic CD4+ T cells induce age-associated myelopoiesis through CCL5–CCR5 signaling
Source: Nat Aging. 2026 Aug 26;6(10):2110–25. doi: 10.1038/s43587-026-01209-9 (PMC13661277; doi:10.1038/s43587-026-01209-9)
Supplement: Supplementary file 4 [file 43587_2026_1209_MOESM4_ESM.pdf]

Supplementary Table. 2

Oligonucleotides for qPCR		
Ccl5	Thermo Fisher Scientific	F: CCTGCTGCTTTGCCTACCTCTC<br>R: ACACACTTGGCGGTTCCTTCGA
Eomes	Thermo Fisher Scientific	F: GTCAACACTTTGCCTCAAGC<br>R: AAGACAGGTGGGCTCATTCT
CD38	Thermo Fisher Scientific	F: GGTCCAAGTGATGCTCAATGGG<br>R: AGCTCCTTCGATGTCGTGCATC
Pdcd1	Thermo Fisher Scientific	F: CGGTTTCAAGGCATGGTCATTGG<br>R: TCAGAGTGTCGTCCTTGCTTCC
Actb	Thermo Fisher Scientific	F: GATGTATGAAGGCTTTGGTC<br>R: TGTGCACTTTTATTGGTCTC
Gapdh	Thermo Fisher Scientific	F: CATCACTGCCACCCAGAAGACTG<br>R: ATGCCAGTGAGCTTCCCGTTCAG
